# Low-dose spironolactone and cardiovascular outcomes in moderate stage chronic kidney disease: a randomized controlled trial

**DOI:** 10.1038/s41591-024-03263-5

**Published:** 2024-09-30

**Authors:** F. D. Richard Hobbs, Richard J. McManus, Clare J. Taylor, Nicholas R. Jones, Joy K. Rahman, Jane Wolstenholme, Sungwook Kim, Joseph Kwon, Louise Jones, Jennifer A. Hirst, Ly-Mee Yu, Sam Mort, on behalf of the BARACK-D Investigators, F. D. Richard Hobbs, R. J. McManus, L. Jones, B. Thompson, J. K. Rahman, C. Vicary, L. Evans, E. Egden, M. Patil, Ly-Mee Yu, S. Mort, J. Wolstenholme, D. Lasserson, C. J. Taylor, N. R. Jones, BARACK-D Investigators, F. D. Richard Hobbs, R. J. McManus, J. Wolstenholme, D. Lasserson, J. Townend, C. Ferro, P. Bower, A. Farmer, D. Fitzmaurice, G. Feder, P. Little, N. Qureshi, Regional coordinating centre teams, R. J. McManus, L. Jones, B. Thompson, J. K. Rahman, D. Lasserson, C. J. Taylor, N. R. Jones, A. Farmer, F. D. Richard Hobbs, R. Perera, D. Timmins, J. Townend, C. Ferro, D. Fitzmaurice, G. Heer, R. Della, H. Duffy, F. McRonald, D. Popoola, K. Jheeta, G. Feder, S. Bryant, M. Taal, Y. Newey, D. Morgan, P. Bower, C. Gardner, V. Lee, T. Blakeman, N. Qureshi, L. Cross-Bardell, C. Brindley, P. Little, J. Barnett, K. Middleton

**Affiliations:** 1 Nuffield Department of Primary Care Health Sciences, University of Oxford, Oxford, UK; 2 Department of Applied Health Sciences, University of Birmingham, Birmingham, UK; 3 Nuffield Department of Population Health, University of Oxford, Oxford, UK; 4 Institute of Cardiovascular Sciences, University of Birmingham, Birmingham, UK; 5 Division of Population Health, University of Manchester, Manchester, UK; 6 Department of Health Sciences, University of Warwick, Coventry, UK; 7 Bristol Medical School, University of Bristol, Bristol, UK; 8 Primary Care Research Centre, University of Southampton, Southampton, UK; 9 Applied Health Research, University of Nottingham, Nottingham, UK; 10 Derby Medical School, Royal Derby Hospital, Derby, UK

**Keywords:** Outcomes research, Chronic kidney disease, Vascular diseases, Randomized controlled trials

## Abstract

Chronic kidney disease (CKD) is associated with a substantial risk of progression to end-stage renal disease and vascular events. The nonsteroidal mineralocorticoid receptor antagonist (MRA), finerenone, offers cardiorenal protection for people with CKD and diabetes, but there is uncertainty if the steroidal MRA, spironolactone, provides the same protection. In this prospective, randomized, open, blinded endpoint trial, we assessed the effectiveness of 25 mg spironolactone in addition to usual care or usual care alone for reducing cardiovascular outcomes in stage 3b CKD among an older community cohort (mean age = 74.8 years and s.d. = 8.1). We recruited 1,434 adults from English primary care, of whom 1,372 (96%) were included in the primary analysis. The primary outcome was time from randomization until the first occurrence of death, hospitalization for heart disease, stroke, heart failure, transient ischemic attack or peripheral arterial disease, or first onset of any condition listed not present at baseline. Across 3 years of follow-up, the primary endpoint occurred in 113 of 677 participants randomized to spironolactone (16.7%) and 111 of 695 participants randomized to usual care (16.0%) with no significant difference between groups (hazard ratio = 1.05, 95% confidence interval: 0.81–1.37). Two-thirds of participants randomized to spironolactone stopped treatment within 6 months, predominantly because they met prespecified safety stop criteria. The most common reason for stopping spironolactone was a decrease in the estimated glomerular filtration rate that met prespecified stop criteria (*n* = 239, 35.4%), followed by participants being withdrawn due to treatment side effects (*n* = 128, 18.9%) and hyperkalemia (*n* = 54, 8.0%). In conclusion, we found that spironolactone was frequently discontinued due to safety concerns, with no evidence that it reduced cardiovascular outcomes in people with stage 3b CKD. Spironolactone should not be used for people with stage 3b CKD without another explicit treatment indication. ClinicalTrials.gov registration: ISRCTN44522369.

## Abstract (web summary)

A randomized controlled trial involving patients with stage 3b chronic kidney disease from primary care reported that in contrast to reported cardiorenal protective effects of nonsteroidal mineralocorticoid receptor antagonists (MRA), the steroidal MRA spironolactone did not reduce mortality or cardiovascular disease hospitalization compared to usual care.

## Main

Chronic kidney disease (CKD) is an important cause of increased mortality and morbidity through increased cardiovascular events and progression to kidney failure, despite current therapies^1–3^. It is a major and under-recognized risk factor for cardiovascular disease (CVD)^4,5^. Indeed, people with CKD are at higher risk of death from CVD than they are from kidney failure^5^. Although the risks of myocardial infarction and other manifestations of coronary artery disease are increased in CKD, the pattern of CVD is atypical, with a much greater incidence of heart failure and sudden cardiac death than seen in the wider population with CVD^6,7^.

The severity of CKD can be defined based on reductions in the estimated glomerular filtration rate (eGFR), from stages 1 to 5, with stage 5 being the most severe. CKD can separately be categorized based on the presence of albumin in the urine and a raised albumin creatinine ratio (ACR). There is a graded inverse relationship between cardiovascular risk and deteriorating kidney function independent of age, sex and other risk factors, whether measured by raised serum creatinine, the eGFR or ACR^8–11^. While the cardiovascular risk of end-stage CKD is extreme, in public health terms, the burden resides in early-stage (CKD stages 1–3) disease, which is more prevalent^12^. A recent systematic review and meta-analysis reported the prevalence of CKD stages 1–3 was 15% and the prevalence of CKD stages 4 and 5 was 0.5%^13^. Stage 3 CKD is subcategorized into stages 3a (eGFR = 45–59 ml min^−1^ 1.73 m^−^^2^) and stage 3b (eGFR = 30–44 ml min^−1^ 1.73 m^−^^2^), partly because of the recognition that people with stage 3b CKD are at increased risk of cardiovascular events compared to those with Stage 3a^14,15^.

Development of therapies that reduce the risk of CVD or progressive kidney disease among people with CKD is therefore a population health priority^16^. Angiotensin-converting enzyme inhibitor (ACEi) or angiotensin receptor blocker (ARB) treatment can be used to block the renin–angiotensin system and has been shown to protect kidney function in people with CKD and albuminuria^17,18^. However, such patients remain at substantial risk of progressive CVD despite ACEi or ARB therapy^19^. Furthermore, 10–53% of patients have reported rises in aldosterone levels within the first year of therapy with an ACEi or ARB, with subsequent pro-inflammatory and pro-fibrotic effects on the circulatory system, including the heart and kidneys^19^. Mineralocorticoid receptor antagonists (MRA), such as spironolactone, may provide more effective aldosterone blockade as well as have wider beneficial effects on the cardiorenal system^19^. For example, preliminary data that informed the rationale for the Benefits of Aldosterone Receptor Antagonism in Chronic Kidney Disease (BARACK-D) trial suggested treatment with spironolactone positively impacted left ventricular function, mass and aortic stiffness in people with CKD stages 2 and 3 who were already established on treatment with an ACEi or ARB^20,21^.

After the BARACK-D trial was initiated, the Finerenone in Reducing Kidney Failure and Disease Progression in Diabetic Kidney Disease (FIDELIO-DKD) and Finerenone in Reducing Cardiovascular Mortality and Morbidity in Diabetic Kidney Disease (FIGARO-DKD) trials both tested a MRA, finerenone, in participants with CKD as well as type 2 diabetes^22,23^. Both studies showed reductions in the risk of CKD progression or cardiovascular events^22–24^. However, both trials only recruited participants with diabetes and excluded people who had nonalbuminuric CKD, meaning the majority of participants had advanced disease^22–24^. The use of MRA in people with moderate CKD, including those without albuminuria and diabetes, therefore remains uncertain.

Furthermore, finerenone is a relatively new, nonsteroidal MRA, in contrast to the older steroidal MRA, spironolactone. Important differences exist between steroidal and nonsteroidal MRA in terms of molecular and pharmacokinetic properties. Steroidal MRAs are less selective and have a relatively greater tissue distribution in the kidneys compared to the heart. Rodent models suggest that nonsteroidal MRAs may lead to more pronounced inhibition of pro-fibrotic and pro-inflammatory changes in the kidneys^25^. It is therefore important to establish if the beneficial effects of the nonsteroidal MRA, finerenone, reported in FIDELIO-DKD and FIGARO-DKD could be reproduced through treatment with the steroidal MRA, spironolactone, given that it is a cheaper medication that is already widely used in clinical practice.

BARACK-D was a phase 3, prospective, randomized, open, blinded endpoint (PROBE) trial^23^. The primary aim of the trial was to evaluate the benefits of low-dose spironolactone plus usual care versus usual care alone in participants with stage 3b CKD treated in primary care in terms of mortality and cardiovascular outcomes. Secondary outcome measures included changes in blood pressure, renal function, natriuretic peptide levels, incidence of hyperkalemia and treatment costs and benefits.

## Results

### Participant disposition

**Fig. 1:**
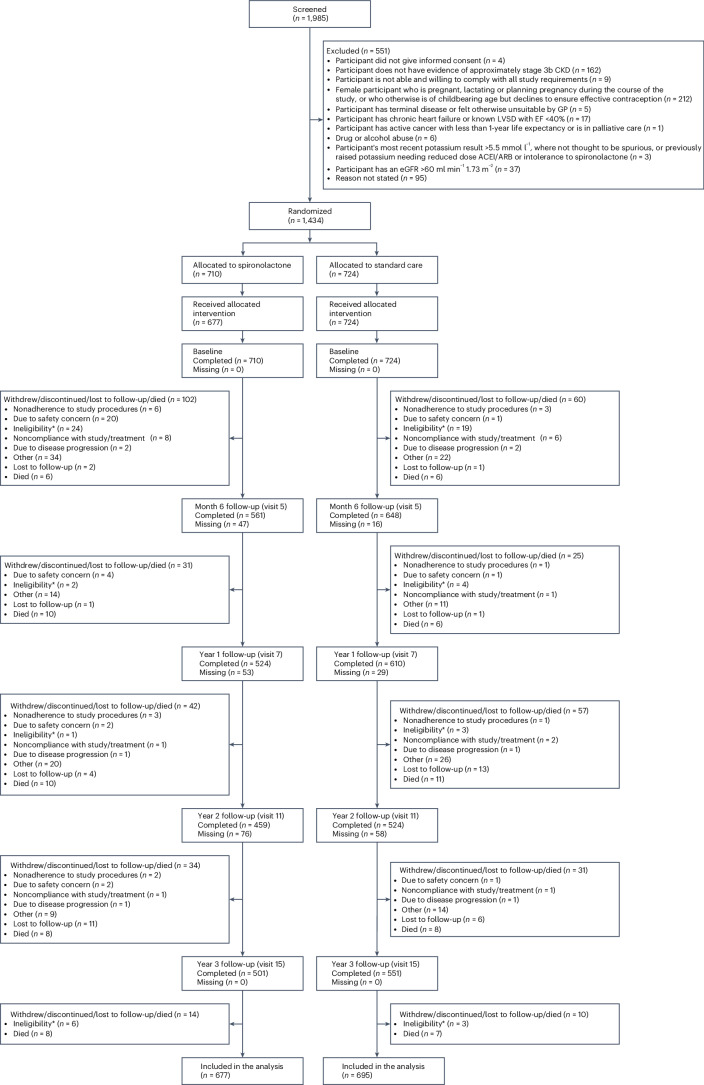
CONSORT flow diagram. The CONSORT flow diagram reports the flow of participants through the study. Note that participants who stopped taking treatment but who continued with the study follow-up remain in their allocated treatment arm. *Participants found to be ineligible after randomisation, excluded from the analysis population.

In total, 1,985 participants attended a screening visit between 6 December 2013 and 31 August 2018, with 1,434 participants randomized into the study (Fig. 1). Of those randomized, 62 participants were identified as ineligible for the study by the supervising trials unit, including 33 participants who had been randomized to spironolactone and 29 randomized to usual care, leaving 1,372 participants who were included in the statistical analyses. The trial failed to recruit to the target, achieving 45% of the planned sample size. There were ten patients in the usual care arm who were initiated on spironolactone at some point during the study by their general practitioner (GP). At the time of randomization, the mean age of participants was 74.8 years (s.d. = 8.1) with 54.5% women (Table 1). There was a high burden of comorbid CVD among the population, including hypertension (76.7%), type 2 diabetes (24.3%), ischemic heart disease (17.4%) and atrial fibrillation (12.2%). The mean eGFR at baseline was 43.5 ml min^−1^ 1.73 m^−^^2^ (s.d. = 6.9) and ACR 1.5 mg mmol^−1^ (interquartile range (IQR) = 0.6–4.3). The majority of participants were prescribed either an ACEi (40.0%) or an ARB (36.6%). Only four participants were prescribed a sodium–glucose cotransporter-2 (SGLT2) inhibitor. The study population was well-balanced between groups.

**Table 1:** Baseline characteristics of participants in the study

Characteristics	Spironolactone	Usual care	Overall
	(*N* = 677)	(*N* = 695)	(*N* = 1,372)
Age (year), mean (s.d.; *N*)	75.1 (8.0; 676)	74.5 (8.3; 695)	74.8 (8.12; 1371)
Age groups (EudraCT guidelines), *n*/*N* (%)			
18–64 years	55/676 (8.1)	64/695 (9.2)	119/1,371 (8.7)
65–84 years	567/676 (83.9)	584/695 (84.0)	1151/1,371 (84.0)
85 years and older	54/676 (8.0)	47/695 (6.8)	101/1,371 (7.4)
Additional age groups, *n*/*N* (%)			
18–54 years	9/676 (1.3)	18/695 (2.6)	27/1,371 (2.0)
55–64 years	46/676 (6.8)	46/695 (6.6)	92/1,371 (6.7)
65–74 years	264/676 (39.1)	267/695 (38.4)	531/1,371 (38.7)
75–84 years	303/676 (44.8)	317/695 (45.6)	620/1,371 (45.2)
85 years and older	54/676 (8.0)	47/695 (6.8)	101/1,371 (7.4)
Gender, *n*/*N* (%)			
Men	306/676 (45.3)	318/695 (45.8)	624/1,371 (45.5)
Women	370/676 (54.7)	377/695 (54.2)	747/1,371 (54.5)
Ethnicity, *n*/*N* (%)			
White	652/673 (96.9)	668/693 (96.4)	1,320/1,366 (96.6)
Mixed/multiple ethnic groups	2/673 (0.3)	4/693 (0.6)	6/1,366 (0.4)
Asian/Asian British	8/673 (1.2)	8/693 (1.2)	16/1,366 (1.2)
Black/African/Caribbean/Black British	8/673 (1.2)	11/693 (1.6)	19/1,366 (1.4)
Other ethnic group	3/673 (0.4)	2/693 (0.3)	5/1,366 (0.4)
Past medical history, *n*/*N* (%)			
Hypertension	508/676 (75.1)	544/695 (78.3)	1052/1,371 (76.7)
Diabetes	165/676 (24.4)	168/695 (24.2)	333/1,371 (24.3)
Impaired fasting glucose and/or glucose tolerance^a^	47/509 (9.2)	57/524 (10.9)	104/1,033 (10.1)
Ischemic heart disease	121/676 (17.9)	118/694 (17.0)	239/1,370 (17.4)
Heart failure	14/676 (2.1)	17/694 (2.4)	31/1,370 (2.3)
Atrial fibrillation	77/676 (11.4)	90/694 (13.0)	167/1,370 (12.2)
Cerebrovascular disease	60/676 (8.9)	77/694 (11.1)	137/1,370 (10.0)
Peripheral vascular disease	27/674 (4.0)	30/694 (4.3)	57/1,368 (4.2)
Childhood urinary tract infection	12/669 (1.8)	22/693 (3.2)	34/1,362 (2.5)
Adulthood urinary tract infection	211/673 (31.4)	223/694 (32.1)	434/1,367 (31.7)
Thyroid disease	105/675 (15.6)	110/693 (15.9)	215/1,368 (15.7)
Anemia	104/674 (15.4)	96/695 (13.8)	200/1,369 (14.6)
Osteopenia	27/673 (4.0)	31/693 (4.5)	58/1,366 (4.2)
Osteoporosis	45/671 (6.7)	41/693 (5.9)	86/1,364 (6.3)
Indices of multiple deprivation quintile, *n*/*N* (%)			
1 (most deprived)	68/664 (10.2)	74/683 (10.8)	142/1,347 (10.5)
2	95/664 (14.3)	95/683 (13.9)	190/1,347 (14.1)
3	122/664 (18.4)	148/683 (21.7)	270/1,347 (20.0)
4	189/664 (28.5)	178/683 (26.1)	367/1,347 (27.2)
5 (least deprived)	190/664 (28.6)	188/683 (27.5)	378/1,347 (28.1)
Current medication, *n*/*N* (%)			
β blockers	181/677 (26.7)	181/695 (26.0)	362/1,372 (26.4)
ACE inhibitors	272/677 (40.2)	277/695 (39.9)	549/1,372 (40.0)
ARBs	248/677 (36.6)	254/695 (36.5)	502/1,372 (36.6)
Statins	409/677 (60.4)	418/695 (60.1)	827/1,372 (60.3)
Antihypertensives	484/677 (71.5)	523/695 (75.3)	1,007/1,372 (73.4)
Smoking status, *n*/*N* (%)			
Never smoker	305/674 (45.3)	337/693 (48.6)	642/1,367 (47.0)
Current smoker	25/674 (3.7)	32/693 (4.6)	57/1,367 (4.2)
Former smoker	344/674 (51.0)	324/693 (46.8)	668/1,367 (48.9)
Weight (kg), mean (s.d.; *n*)	82.0 (15.6; 675)	81.6 (16.2; 695)	81.8 (15.9; 1370)
Height (cm), mean (s.d.; *n*)	165.8 (9.6; 676)	166.2 (9.7; 695)	166.0 (9.6; 1,371)
Waist circumference (cm), mean (s.d.; *n*)	100.7 (13.0; 673)	100.5 (13.2; 687)	100.6 (13.1; 1,360)
Hip circumference (cm), mean (s.d.; *n*)	109.3 (11.3; 672)	108.8 (11.8; 686)	109.0 (11.6; 1,358)
Office BP measurement (mm Hg), mean (s.d.; *n*)			
Systolic BP on the left arm	138.2 (18.2; 669)	136.8 (18.0; 689)	137.5 (18.1; 1.358)
Systolic BP on the right arm	139.0 (18.2; 673)	137.8 (18.4; 693)	138.4 (18.3; 1,366)
Diastolic BP on the left arm	77.4 (11.1; 669)	76.1 (11.3; 689)	76.7 (11.2; 1,358)
Diastolic BP on the right arm	77.3 (10.8; 673)	76.3 (11.4; 693)	76.8 (11.1; 1,366)
Laboratory and ECG test results			
Renal profile, *n*/*N* (%)			
Normal	79/673 (11.7)	77/693 (11.1)	156/1,366 (11.4)
Abnormal (not clinically significant)	531/673 (78.9)	537/693 (77.5)	1068/1,366 (78.2)
Abnormal (clinically significant)	63/673 (9.4)	79/693 (11.4)	142/1,366 (10.4)
Liver function tests, *n*/*N* (%)			
Normal	568/674 (84.3)	563/695 (81.0)	1,131/1,369 (82.6)
Abnormal (not clinically significant)	100/674 (14.8)	131/695 (18.8)	231/1,369 (16.9)
Abnormal (clinically significant)	6/674 (0.9)	1/695 (0.1)	7/1,369 (0.5)
Bone profile, *n*/*N* (%)			
Normal	595/668 (89.1)	589/688 (85.6)	1,184/1,356 (87.3)
Abnormal (not clinically significant)	70/668 (10.5)	98/688 (14.2)	168/1,356 (12.4)
Abnormal (clinically significant)	3/668 (0.4)	1/688 (0.1)	4/1,356 (0.3)
Lipids, *n*/*N* (%)			
Normal	458/669 (68.5)	459/683 (67.2)	917/1,352 (67.8)
Abnormal (not clinically significant)	194/669 (29.0)	208/683 (30.5)	402/1,352 (29.7)
Abnormal (clinically significant)	17/669 (2.5)	16/683 (2.3)	33/1,352 (2.4)
Full blood count, *n*/*N* (%)			
Normal	385/672 (57.3)	383/688 (55.7)	768/1,360 (56.5)
Abnormal (not clinically significant)	271/672 (40.3)	289/688 (42.0)	560/1,360 (41.2)
Abnormal (clinically significant)	16/672 (2.4)	16/688 (2.3)	32/1,360 (2.4)
HbA1C, *n*/*N* (%)			
Normal	456/665 (68.6)	501/688 (72.8)	957/1,353 (70.7)
Abnormal (not clinically significant)	171/665 (25.7)	149/688 (21.7)	320/1,353 (23.7)
Abnormal (clinically significant)	38/665 (5.7)	38/688 (5.5)	76/1,353 (5.6)
Fasting blood sugar, *n*/*N* (%)			
Normal	479/622 (77.0)	513/643 (79.8)	992/1,265 (78.4)
Abnormal (not clinically significant)	116/622 (18.6)	113/643 (17.6)	229/1,265 (18.1)
Abnormal (clinically significant)	27/622 (4.3)	17/643 (2.6)	44/1,265 (3.5)
B-type natriuretic peptide, *n*/*N* (%)			
Normal	439/598 (73.4)	460/608 (75.7)	899/1,206 (74.5)
Abnormal (not clinically significant)	119/598 (19.9)	111/608 (18.3)	230/1,206 (19.1)
Abnormal (clinically significant)	40/598 (6.7)	37/608 (6.1)	77/1,206 (6.4)
ECG, *n*/*N* (%)			
Normal	431/618 (69.7)	458/622 (73.6)	889/1,240 (71.7)
Abnormal (not clinically significant)	161/618 (26.1)	139/622 (22.3)	300/1,240 (24.2)
Abnormal (clinically significant)	26/618 (4.2)	25/622 (4.0)	51/1,240 (4.1)
ACR (mg mmol^−1^), median (IQR, *n*)	1.5 (0.7–4.4; 633)	1.5 (0.6–4.2; 637)	1.5 (0.6–4.3; 1,270)
eGFR (ml min^−1^ 1.73 m^−^^2^), mean (s.d., *n*)	43.9 (6.9; 676)	43.1 (6.8; 695)	43.5 (6.9; 1,371)
Potassium (mmol l^−1^), mean (s.d., *n*)	4.4 (0.4; 677)	4.5 (0.4; 695)	4.5 (0.4; 1,372)
Creatinine (μmol l^−1^), mean (s.d., *n*)	122.8 (23.3; 677)	125.2 (25.0; 695)	124.0 (24.2; 1,372)

Note that percentages have been computed with the number of participants with the response available as the denominator.

We did not collect absolute values for people with a normal result. Participants had a blood test taken at baseline and also an ECG, where available at their GP surgery. These results were reviewed by their own GP, who reported the results back to the study team as either normal, abnormal (not clinically significant) or abnormal (clinically significant). Absolute values were not collected at these stages, only this categorical data.

^a^Only includes those without diabetes.

BP, blood pressure; ECG, electrocardiogram.

At the end of 3 years of follow-up, 1,052 participants remained in the study. The median follow-up time was 3 years. Overall mean follow-up was 131 weeks, with a mean follow-up of 126 weeks in the spironolactone arm, compared to 135 weeks in the usual care arm. There were 182 (26.9%) participants in the spironolactone group who withdrew, discontinued or were lost to follow-up, compared to 147 (21.2%) in the usual care group (odds ratio 1.37, 95% confidence interval (CI): 1.07–1.76, *P* = 0.013). However, only a third of patients continued taking spironolactone beyond 6 months, although many of these participants continued trial follow-up. The median time from randomization to withdrawal from treatment was 3.2 months (IQR: 0.98–18.1).

The study was ongoing during the COVID-19 pandemic, which impacted the planned follow-up, especially blood monitoring. For example, 88.0% of participants completed the year 2 study visit, compared to 95.0% at 6 months. Although the primary endpoint components were available for all participants, at 3-year follow-up, only 71.1% of participants had a systolic blood pressure recorded and 71.4% an eGFR. Both groups were affected similarly by this disruption to follow-up.

### Primary outcome

**Fig. 2:**
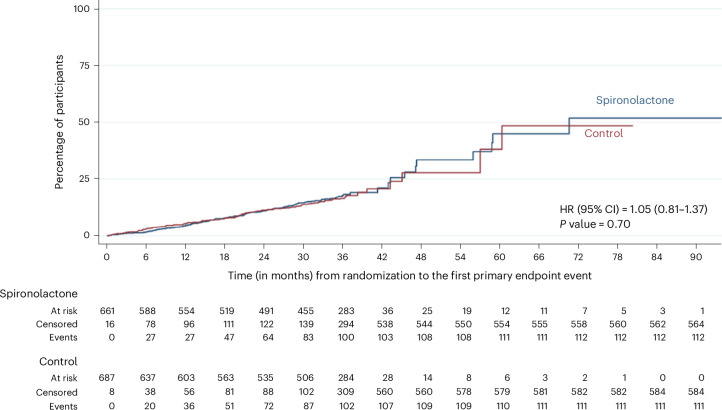
Kaplan–Meier curve for the time (in months) from randomization to the first primary endpoint event. Kaplan–Meier curve for the composite primary outcome comparing those randomized to spironolactone versus usual care (two-sided test).

There was no significant difference in the number of participants who experienced the composite primary outcome (defined as the time from randomization until the first occurrence of death, hospitalization for heart disease (coronary heart disease, arrhythmia, atrial fibrillation, sudden death and failed sudden death), stroke, heart failure, transient ischemic attack or peripheral arterial disease (PAD), or first onset of any condition listed not present at baseline) between groups (Fig. 2)—113 primary outcome events among the 677 participants randomized to spironolactone (16.7%) compared to 111 among the 695 participants (16.0%) in the usual care arm (hazard ratio (HR) = 1.05, 95% CI: 0.81–1.37, *P* = 0.702). Summary statistics for the primary are shown in Table 2. The incidence rate per 100 years at risk was 6.83 among participants prescribed spironolactone, compared to 6.27 among those in the usual care group and 6.54 among all participants.

**Table 2:** Summary statistics and the HRs for the primary analysis

Parameters	Spironolactone (*N* = 677)	Usual care (*N* = 695)	HR (95% CI)^a^	*P* value^b^
Primary analysis				
Primary endpoint^c^				
Experienced, *n*/*N* (%)	113/677 (16.7)	111/695 (16.0)	–	–
Time at risk (year; incidence rate per 100 years at risk)^d^	1653.9 (6.83)	1769.8 (6.27)	1.05 (0.81–1.37)	0.702
Primary endpoint component: hospitalization^c^				
Experienced, *n*/*N* (%)	39/677 (5.8)	42/695 (6.0)	–	–
Time at risk (year; incidence rate per 100 years at risk)	1703.4 (2.29)	1807.9 (2.32)	0.99 (0.64–1.53)	0.970
Primary endpoint component: CVD^c^				
Experienced, *n*/*N* (%)	66/677 (9.7)	61/695 (8.8)	–	–
Time at risk (year; incidence rate per 100 years at risk)	1661.0 (3.97)	1780.7 (3.43)	1.14 (0.80–1.61)	0.478
Primary endpoint component: death^c^				
Experienced, *n*/*N* (%)	42/677 (6.2)	38/695 (5.5)	–	–
Time at risk (year; incidence rate per 100 years at risk)	1767.4 (2.38)	1889.7 (2.01)	1.09 (0.70–1.70)	0.699

^a^Spironolactone versus usual care.

^b^Level of significance = 0.05.

^c^Cox proportional hazards model adjusted for a randomized arm.

^d^Primary endpoint.

### Secondary clinical outcomes

There were no significant differences between groups for any of the individual components of the primary endpoint, including hospitalization (HR = 0.99, 95% CI: 0.64–1.53, *P* = 0.970), CVD (HR = 1.14, 95% CI: 0.80–1.61, *P* = 0.478) or death (HR = 1.09, 95% CI: 0.70–1.70, *P* = 0.699; Table 2). In total, 6.0% of participants died, including 42 (6.2%) deaths among the intervention group and 38 (5.5%) among those randomized to usual care.

Among participants randomized to spironolactone compared to usual care, there was a small reduction in mean eGFR over follow-up at 3 years (adjusted treatment effect −1.14 ml min^−1^ 1.73 m^−2^, 95% CI: −1.92 to −0.37, *P* = 0.004; Table 3). A modest deterioration in mean eGFR was observed in the spironolactone arm at 6 months to 42 ml min^−1^ 1.73 m^−2^, which then remained stable at 1, 2 and 3 years. The mean eGFR in the control group was stable for up to 2 years and then a small decline to 42 ml min^−1^ 1.73 m^−2^ at year 3. Although small differences, these slightly lower mean eGFRs in the spironolactone participants were statistically significant at each follow-up time point (Table 3). Among those randomized to spironolactone, the adjusted treatment effects for eGFR at 6 months, 1 year, 2 years and 3 years, respectively, were −1.68 ml min^−1^ 1.73 m^−2^ (95% CI: −2.41 to −0.94), −2.33 ml min^−1^ 1.73 m^−2^ (95% CI: −3.08 to −1.58), −0.89 ml min^−1^ 1.73 m^−2^ (95% CI: −1.69 to −0.09) and −1.14 ml min^−1^ 1.73 m^−2^ (95% CI: −1.92 to −0.37; Table 3).

**Table 3:** Summary statistics and the adjusted treatment differences for the secondary analyses

Parameters	Spironolactone (*N* = 677)	Usual care (*N* = 695)	Adjusted treatment effect (95% CI)^a^	*P* value^b^
Secondary analyses				
Office measurements of systolic blood pressure (mm Hg), mean (s.d.) **(*n*)**^c^				
Baseline	138.6 (17.66) **(677)**	137.3 (17.51) **(695)**	–	–
6 months	131.2 (16.03) **(553)**	134.1 (15.87) **(636**)	−3.32 (−5.05 to −1.59)	<0.001
1 year	130.9 (15.86) **(518)**	133.4 (16.15) **(603)**	−2.66 (−4.43 to −0.90)	0.003
2 years	132.0 (17.21) **(434)**	133.5 (15.91) **(494)**	−1.33 (−3.22 to 0.56)	0.169
3 years	134.2 (16.46) **(460)**	134.8 (16.34) **(515)**	−1.69 (−3.55 to 0.16)	0.074
Rate of hypotension, *n*/*N* (%)^d^				
During the study	49/658 (7.4)	32/686 (4.7)	1.60 (1.04–2.46)	0.034
Natriuretic peptide (pg ml^−1^), mean (s.d.) **(*n*)**^c^				
Baseline	311.8 (505.01) **(677)**	323.8 (477.94) **(695)**	–	–
6 months	289.3 (819.26) **(451)**	308.6 (511.56) **(512)**	−1.30 (−1.63 to −1.03)	0.026
1 year	302.4 (513.36) **(425)**	358.2 (626.15) **(475)**	−1.28 (−1.61 to −1.01)	0.039
2 years	360.8 (604.48) **(352)**	367.3 (592.75) **(396)**	−1.09 (−1.39 to 1.17)	0.487
3 years	384.6 (568.18) **(363)**	451.6 (740.31) **(413)**	−1.20 (−1.53 to 1.06)	0.146
ACR (mg mmol^−1^), *n*/*N* (%) and mean (s.d.) **(*n*)**^e^				
Baseline	4.9 (8.43) **(677)**	5.3 (9.38) **(695)**	–	–
<3	429/677 (63.4)	439/695 (63.2)	*–*	*–*
3–30	229/677 (33.8)	233/695 (33.5)	*–*	*–*
>30	19/677 (2.8)	23/695 (3.3)	*–*	*–*
3 years	10.5 (36.07) **(361)**	8.2 (18.22) **(403)**	1.03 (−1.50 to 1.59)	0.897
<3	212/361 (58.7)	253/403 (62.8)	*–*	*–*
3–30	123/361 (34.1)	116/403 (28.8)	*–*	*–*
>30	26/361 (7.2)	34/403 (8.4)	*–*	*–*
eGFR (ml min^−1^ 1.73 m^−^^2^), mean (s.d.) **(*n*)**^c^				
Baseline	43.9 (6.90) **(677)**	43.1 (6.79) **(695)**	–	–
6 months	42.1 (8.01) **(550)**	43.2 (7.22) **(635)**	−1.68 (−2.41 to −0.94)	<0.001
1 year	42.0 (8.13) **(515)**	43.7 (8.09) **(599)**	−2.33 (−3.08 to −1.58)	<0.001
2 years	42.5 (8.79) **(425)**	43.0 (8.33) **(484)**	−0.89 (−1.69 to −0.09)	0.029
3 years	41.7 (9.18) **(462)**	42.0 (8.75) **(518)**	−1.14 (−1.92 to −0.37)	0.004

Numbers in bold refer to the number of participants in each analysis.

^a^Spironolactone versus usual care.

^b^Level of significance = 0.05.

^c^Linear mixed effects model adjusted for randomized arm, baseline measurement, assessment time point, an interaction between the randomized arm and assessment time point, and baseline factors that predict missingness of the endpoint as a fixed effect, and a random intercept for each participant.

^d^Log-binominal regression model adjusted for randomized arm, and baseline factors that predict missingness of the endpoint as covariates.

^e^Linear regression model adjusted for randomized arm, baseline measurement and baseline factors that predict missingness of the endpoint as covariates.

At 1-year follow-up, there was an initial relative reduction in systolic blood pressure among those randomized to spironolactone (adjusted treatment effect −2.66 mm Hg, 95% CI: −4.43 to −0.90, *P* = 0.003). However, there was no difference in systolic blood pressure between groups by the end of follow-up at 3 years (adjusted treatment effect −1.69 mm Hg, 95% CI: −3.55 to 0.16, *P* = 0.074; Table 3).

At 3 years, mean ACR levels had worsened in both arms from means of 4.9–10.5 mg mmol^−1^ and 5.3–8.2 mg mmol^−1^ in the spironolactone versus control arms, respectively. There was no significant difference between groups in the ACR at either time point or in a change in urine ACR over time (adjusted treatment effect 1.03 mg mmol^−1^, 95% CI: −1.5 to 1.59, *P* = 0.897; Table 3).

In terms of natriuretic peptide levels, the participants randomized to spironolactone recorded significantly lower levels than control participants at 6 months (−1.30 pg  ml^−1^ mean (−1.63 to −1.03), *P* = 0.026) and at 1 year (−1.28 pg ml^−1^ mean (−1.61 to −1.01), *P* = 0.039), but no difference was observed at 2 and 3 years, after adjustment for the highly skewed levels and missing values (Table 3). Mean natriuretic peptide levels rose during follow-up in both arms of the study, exceeding the mean baseline levels in the control group by 12 months and in the spironolactone group by 2 years.

### Healthcare cost evaluation

A within-trial health economic evaluation was done for the secondary cost-effectiveness outcomes. This further indicated that the added treatment of spironolactone to usual care was not cost-effective when compared with usual care. This remained the case after multiple imputations were performed and in a sensitivity analysis using EuroQol-visual analog scales (EQ-VAS; rescaled to 0–1) as an alternative health-related quality of life (HRQoL) weight to calculate the quality-adjusted life years (QALY).

**Extended Data Fig. 1:**
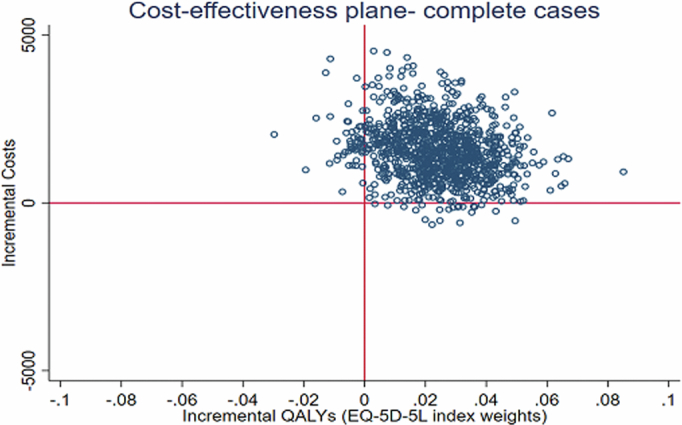
Cost-effectiveness plane for spironolactone in addition to usual care versus usual care alone under complete case analysis using EQ-5D-5L weights for the quality-adjusted life years (QALY). The cost-effectiveness plane presents the results from 1,000 bootstrap replications, each represented by an individual circle.

**Extended Data Fig. 2:**
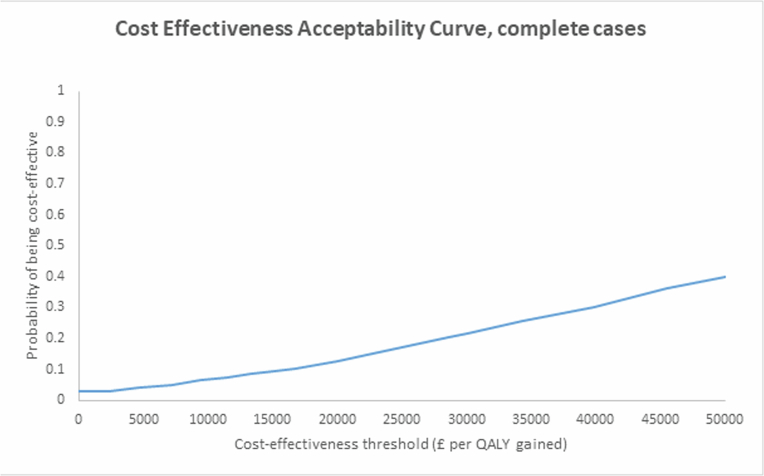
Cost-effectiveness acceptability curve for spironolactone in addition to usual care versus usual care alone under complete case analysis with EQ-5D-5L weights for the quality-adjusted life years (QALY). The cost-effectiveness acceptability curve presents the probability of spironolactone in addition to usual care being cost-effective compared to usual care alone at different cost-effectiveness thresholds.

Extended Data Fig. 1 shows the scatter plot on the cost-effectiveness plane of the bootstrap replicates using the complete case dataset, with most replicates in the northeast quadrant with incremental cost and QALY. Extended Data Fig. 2 shows the cost-effectiveness acceptability curve using complete cases for the probability of spironolactone plus usual care being cost-effective versus usual care across a range of cost-effectiveness thresholds. At the cost-effectiveness threshold of £20,000 per QALY gained, there was a 12.6% probability of spironolactone plus usual care being cost-effective versus usual care and 21.8% at the £30,000 per QALY gained threshold.

There was no significant difference in total healthcare resource use costs comparing between treatment groups at any follow-up time point nor a significant difference in the subcategories of primary care, secondary care or medication costs. Total mean healthcare resource use costs at 3-year follow-up for people randomized to spironolactone were £1,080 compared to £1,136 with usual care, a mean difference of −£56 (bootstrapped 95% CI: −£463 to £378, *P* = 0.796). There was no difference in mean EuroQol five-dimensions five-levels (EQ-5D-5L) scores between the two treatment arms at baseline, 6-month, 1-year or 2-year follow-up. However, by 3-year follow-up, spironolactone was associated with an increased mean score (0.751) compared to usual care (0.719) that was statistically significant (mean difference 0.032, 95% CI: 0.001–0.060, *P* = 0.037). There was no significant difference in Kidney Disease Quality of Life scores between groups across follow-up, although there was a statistically significant difference in favor of usual care for the burden of kidney disease component of the score at 1-year (spironolactone 91.0, usual care 93.2; *P* = 0.017) and a statistically significant difference in favor of spironolactone in the physical composite scale at 3-years in the complete case analysis (spironolactone 42.9, usual care 39.6; *P* = 0.008).

### Safety

In total, 455 participants randomized to spironolactone had treatment withdrawn because of safety concerns. The most common reason (*n* = 239, 35.4%) was a decrease in eGFR that met the prespecified stop criteria, followed by 128 (18.9%) participants being withdrawn due to potential treatment side effects.

The frequency of adverse events is shown in Table 4 (with classification by Medical Dictionary for Regulatory Activities (MedDRA) codes shown in Supplementary Table 1). An additional 33 participants who were randomized to spironolactone but who did not take any medication were included in the comparator usual care arm in this analysis. There were significantly more episodes of hypotension (systolic blood pressure <100 mm Hg or a drop in the systolic pressure of >20 mm Hg on standing) experienced across all individuals in the spironolactone versus the control arm (49 versus 32, adjusted treatment effect 1.6 (95% CI: 1.04–2.46), *P* = 0.034), with 11 participants citing hypotension as a reason for withdrawal from treatment (Tables 3 and 4). Participants who received spironolactone experienced significantly more episodes of hyperkalemia (24.7%) overall versus 13.4% in the control arm, defined by a potassium of ≥5.5 mmol l^−1^. Most of these were mild (potassium 5.5–5.9 mmol l^−1^) in both arms, but 11% of the raised potassium events that occurred were levels of ≥6 mmol l^−1^ and therefore required dose suspension or adjustment, and 8.0% (*n* = 54) of participants were withdrawn from spironolactone treatment for reasons related to hyperkalemia. Among patients who experienced hyperkalemia, the mean baseline eGFR was 41.9 ml min^−^^1^ 1.73 m^−2^ (s.d. 7.08) compared to a mean baseline eGFR of 43.9 ml min^−^^1^ 1.73 m^−2^ (s.d. 6.92) among those who did not experience hyperkalemia.

**Table 4:** Frequency and percentage of hyperkalemia, adverse events and serious adverse events

Parameters	Spironolactone (*N* = 677)	Usual care (*N* = 757)	*P* value^a^
Safety analysis			
Hyperkalemia, *n*/*N* (%)^b^			
Experienced	167/676 (24.7)	95/708 (13.4)	<0.001
Mild (*K* + 5.5–5.9 mmol l^−1^)	147/167 (88.0)	85/95 (89.5)	
Moderate (*K* + 6.0–6.4 mmol l^−1^)	16/167 (9.6)	10/95 (10.5)	
Severe (*K* + >6.5 mmol l^−1^)	4/167 (2.4)	0/95 (0.0)	
Hypotension, *n*/*N* (%)^c^	49/658 (7.4)	32/686 (4.7)	0.034
Adverse events (AEs), *n*/*N* (%)^b^			
Experienced at least one	562/677 (83.0)	384/757 (50.7)	<0.001
None	115/677 (17.0)	373/757 (49.3)	
1	259/677 (38.3)	192/757 (25.4)	
2	153/677 (22.6)	89/757 (11.8)	
3	69/677 (10.2)	52/757 (6.9)	
4	37/677 (5.5)	24/757 (3.2)	
≥5	44/677 (6.5)	27/757 (3.6)	
Severity of AEs, *n*/*N* (%)^d^			
*N* (number of AEs)	1,179	789	0.017
Mild	778/1,178 (66.0)	519/789 (65.8)	
Moderate	325/1,178 (27.6)	194/789 (24.6)	
Severe	75/1,178 (6.4)	76/789 (9.6)	
Plausible relationship of AEs to study drug, *n*/*N* (%)^b^			
*N* (number of AEs)	1,179	789	<0.001
Not related	523/1,178 (44.4)	786/789 (99.6)	
Possibly related	351/1,178 (29.8)	2/789 (0.3)	
Probably related	229/1,178 (19.4)	1/789 (0.1)	
Definitely related	75/1,178 (6.4)	0/789 (0.0)	
Serious AEs, *n*/*N* (%)^b^			
Experienced at least one	103/677 (15.2)	113/757 (14.9)	0.883
None	574/677 (84.8)	644/757 (85.1)	
1	68/677 (10.0)	84/757 (11.1)	
2	29/677 (4.3)	17/757 (2.2)	
≥3	6/677 (0.9)	12/757 (1.6)	

Note that one patient in the spironolactone arm did not have the severity of the adverse event recorded, and one did not have it recorded whether there was a plausible relationship between the adverse event and the study drug.

^a^Spironolactone versus usual care. Level of significance = 0.05.

^b^Fisher’s exact test.

^c^Log-binominal regression model adjusted for randomized arm, and baseline factors that predict missingness of the endpoint as covariates.

^d^Chi-squared test.

The participants on spironolactone were also more likely to experience an adverse event during the trial, to encounter more multiple adverse events and to have such events related to the spironolactone. However, the severity of these events and the proportion of serious adverse events were similar between the intervention and control groups.

### Exploratory outcomes

**Extended Data Fig. 3:**
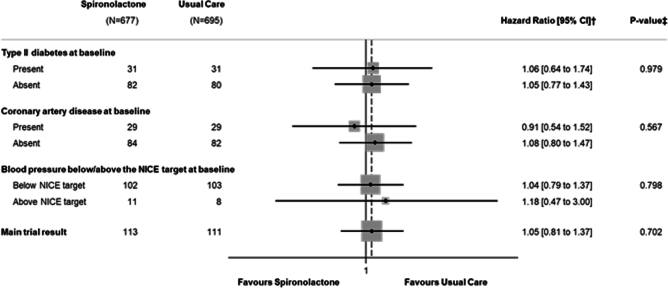
Forest plot of the hazard ratio and 95% confidence interval from the subgroup analyses compared to the main trial result (dotted vertical line). †Spironolactone versus standard care among sub-groups of participants with type 2 diabetes, coronary artery disease or blood pressure above or below the NICE target at baseline. Cox proportional hazards model adjusted for randomized arm, an indicator variable for the subgroup and an interaction between randomized arm and the subgroup indicator variable. A hazard ratio of less than 1 indicates improvement in favor of spironolactone. ‡Two-sided P-value. Level of significance = 0.05.

This trial was not designed to detect subgroup effects and thus lacked statistical power. All subgroup analyses should be considered exploratory in nature. The HR between the randomized groups with a 95% CI and the associated *P* values from the test of interaction were obtained for three prespecified subgroups of interest (type 2 diabetes at baseline, coronary artery disease at baseline and blood pressures below or above the National Institute for Health and Care Excellence (NICE) target at baseline; Extended Data Table 1). A forest plot of these results shows that there were no differences in relation to the combined primary outcome among any of these subgroups (Extended Data Fig. 3).

### Prespecified sensitivity analysis

The Cox proportional hazard model used in the primary analysis section was rerun with an indicator variable for the same three subgroups of interest as an additional covariate in the model (Extended Data Table 2). This did not substantially change the summary result (HR = 1.03, 95% CI: 0.79–1.35).

### Post hoc analyses

**Extended Data Fig. 4:**
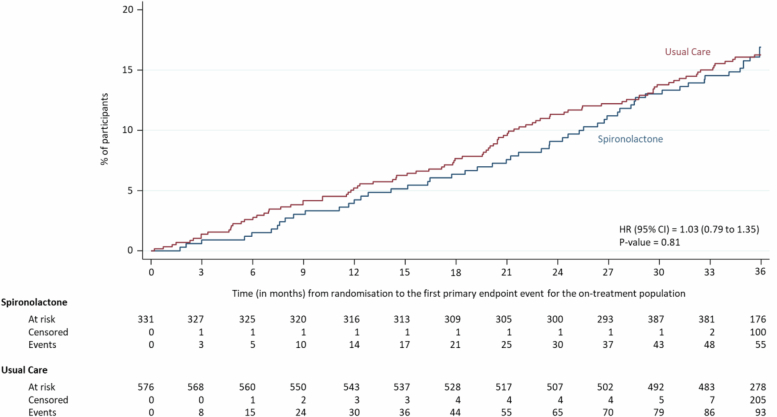
Kaplan–Meier curve for the time (in months) from randomization to the first primary endpoint for the post hoc analysis based on a per-protocol principle and an on-treatment population. Spironolactone versus usual care. Cox proportional hazards model adjusted for randomized arm. A hazard ratio of less than 1 indicates improvement in favor of spironolactone. Two-sided P-value. Level of significance = 0.05.

**Extended Data Fig. 5:**
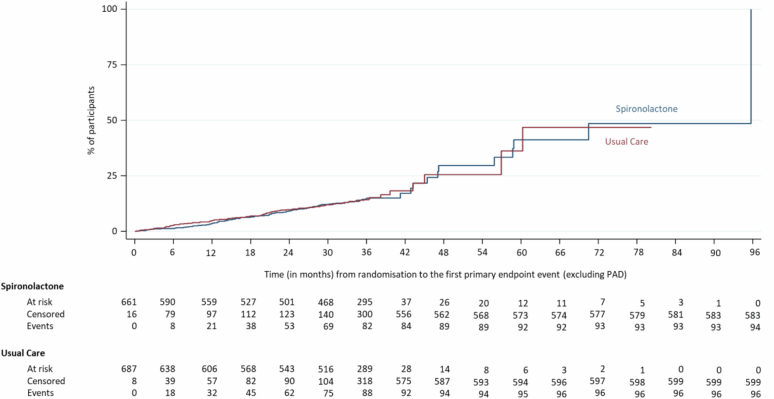
Kaplan–Meier curve for the time (in months) from randomization to the first primary endpoint, excluding PAD from the composite primary outcome definition. Spironolactone versus usual care (two-sided test), excluding PAD from the composite primary outcome. P-value level of significance = 0.05. PAD: peripheral arterial disease.

The slow rate of recruitment meant the follow-up of earlier recruits was longer than planned, which would have increased the chance of competing risks influencing the primary outcomes of interest. We, therefore, performed a post hoc per-protocol sensitivity analysis restricted to events observed in 3 years of follow-up of participants, as originally planned (Extended Data Fig. 4). This analysis showed no difference in rates of death, new-onset CVD or hospitalization in these participants with CKD stage 3b receiving spironolactone compared to usual care (Extended Data Fig. 4). Because of the late correction of PAD in the composite primary outcome (Methods), we also performed a post hoc sensitivity of the primary analysis with and without PAD, which showed no difference (Extended Data Fig. 5).

In the post hoc conditional power calculations, the average conditional probability power was 30.4% when event rates were simulated based on the previous data used to inform our sample size calculation, but only 11.8% when simulated based on the observed event rate within our study.

Finally, we performed a post hoc subgroup analysis of the primary outcome and safety analysis by participants’ gender. The HR for the primary outcome among women was 1.15 (95% CI: 0.77–1.71) and among men was 0.97 (0.68–1.38; Extended Data Table 3). Safety events were similar between men and women (Extended Data Tables 4 and 5), although the trial was not powered to detect differences between genders.

## Discussion

In this randomized PROBE trial, we found that low-dose spironolactone did not reduce the risk of a composite outcome of death, hospitalization for CVD or new cardiovascular events compared to usual care among a community cohort of people (mean age = 74.8 years) with moderate CKD and a high burden of cardiovascular comorbidity. We also found no reduction in risk in any component of the composite outcome nor in our prespecified subgroups of participants with coronary artery disease, type 2 diabetes or uncontrolled hypertension at baseline. In the within-trial economic analysis, spironolactone was not effective compared to usual care. Although difficulties with recruitment left the study underpowered, based on the anticipated effect size, this is unlikely to have influenced the outcomes. The conditional power calculation suggests that there is an 11.8% probability that a statistically significant result would have been found if the trial had reached its recruited target and the observed event rate remained constant with the participants who were recruited. Spironolactone appeared to be poorly tolerated among the study population, and only a third of participants continued on treatment beyond 6 months post-randomization. Participants randomized to spironolactone did have a small but statistically significant greater deterioration in eGFR across follow-up. These participants were also more likely to have hyperkalemia and hypotension. Our results suggest that spironolactone should not be used in people with CKD without another specific indication, such as heart failure.

The trial demonstrated the high cardiovascular risk associated with CKD stage 3b with the primary endpoint occurring in over 16% of participants, including 6% who died. The incidence rate per 100 years at risk was 6.54, which compares to the combined event rate for cardiovascular events or death of 16.05 per 100 person-years reported in ref. ^2^ used to power the trial. The absolute rates of cardiovascular events in BARACK-D were slightly higher than in other landmark trials of CKD, which may in part reflect the relatively older population recruited into our study but may also reflect differences in the composite primary outcome definition between trials^26,27^.

The results of our study of the steroidal MRA, spironolactone, differ from previous randomized trials of the nonsteroidal MRA, finerenone, which demonstrated that finerenone reduced the risk of progression of CKD or incident CVD among people with albuminuric CKD and type 2 diabetes^24^. The FIGARO-DKD study reported that finerenone led to a 13% relative reduction (HR = 0.87, 95% CI: 0.76–0.98, *P* = 0.03) in the risk of the primary composite outcome of death from cardiovascular cause, nonfatal myocardial infarction, nonfatal stroke or hospitalization for heart failure, compared to placebo^23^. In the FIDELIO-DKD study, randomization to finerenone led to a 14% reduction (HR = 0.86, 95% CI: 0.75–0.99, *P* = 0.03) in the primary composite outcome of kidney failure, a sustained 40% reduction in eGFR from baseline or death from renal cause^22^. The FIDELITY study pooled individual patient data from these two studies and confirmed these observations among the larger pool of trial participants^24^.

Although it is possible that our study failed to detect a treatment effect of spironolactone because of the comparatively small sample size, there is no suggestion of this from the data. Two-thirds of participants randomized to spironolactone discontinued treatment within the first 6 months, largely due to them meeting prespecified ‘stop’ criteria, such as prespecified reductions in renal function. The high number of participants who discontinued spironolactone treatment at an early stage may be important in explaining our results and implies that poor tolerability of spironolactone is a barrier to its use in this population. The difference in results between the finerenone trials and the BARACK-D trial may be explained by the differences in tolerability and efficacy of spironolactone as a steroidal MRA compared to the nonsteroidal MRA, finerenone. Finerenone may have additional benefits compared to spironolactone, such as an antifibrotic effect in the heart and inhibition of the expression of pro-inflammatory markers in the kidney^25,28^. Finerenone is also associated with a low risk of hyperkalemia and hypotension, meaning discontinuation rates are likely to be lower than for spironolactone, and more participants are able to continue on treatment^23^.

The differences in trial results may also partly relate to differences in the study populations. The BARACK-D trial population were mainly older participants (mean age of 74.8 years versus median age of 64.8 years in the FIDELITY study), with CKD mainly defined by age-related kidney impairment. In contrast, the finerenone trial populations involved CKD participants mainly defined by kidney damage in the context of diabetes^22,23^. The mean baseline eGFR in our study was lower than the FIDELITY study (43.5 versus 57.5 ml min^−1^ 1.73 m^−^^2^), but 98.2% of those in the FIDELITY study had moderate to significant proteinuria, compared to a mean baseline urinary ACR of just 1.5 mg mmol^−1^ (IQR = 0.6–4.3) in BARACK-D^24^. Despite the encouraging results of finerenone, our study suggests that the results cannot be immediately applied to older populations with CKD without diabetes or albuminuria.

Because of the design of the current trial, recent large randomized controlled trials have demonstrated that SGLT2 inhibitors can slow CKD progression and death from renal or cardiovascular causes among people with CKD, with or without type 2 diabetes^29–31^. The SGLT2 inhibitor trial findings have informed updates in guideline recommendations and are likely to substantially change the management of patients with CKD in practice^32^. However, SGLT2 inhibitors were used infrequently in primary care during the BARACK-D study period, with only four of our participants prescribed these treatments. This is in line with an observational study reporting that 11% of patients with type 2 diabetes were prescribed an SGLT2 inhibitor in 2019 (ref. ^33^). These data indicate that we can be confident that our results were not affected by SGLT2 inhibitor prescribing.

Our study was a large, randomized primary care study that managed to recruit a sample of participants that was older and with a more equal proportion of genders than previous randomized studies of patients with CKD. Given that CKD is common with increasing age, our findings are likely to be generalizable to the populations treated with CKD in primary care settings in high-income countries.

Limitations of the study include that we recruited only around half the number of participants we originally planned (>3,000 participants) due to difficulties with recruitment, despite over 300 GP surgeries acting as recruitment sites. This potentially risks the study being underpowered based on our original power calculations. However, no trend was seen toward a treatment effect in the primary analysis or any of its composite outcomes. Furthermore, the post hoc conditional power analysis suggests that the results would not have changed if we had recruited the planned numbers. Follow-up of participants was affected by the COVID-19 pandemic, particularly for data that could not be collected remotely, such as eGFR. Data for the primary outcome were complete for all randomized and eligible participants who completed follow-up.

Another potential limitation was that we did not use a placebo control in this study, meaning participants and their treating clinicians were unblinded to the intervention. This may have had an impact on the withdrawal and discontinuation rates, which were higher among people randomized to spironolactone, as treatment may have been stopped as a precaution or through attribution of symptoms to the intervention. Stop rules were implemented to protect participants’ safety, but these did risk trial treatment being terminated for some participants with a temporary change in kidney function or potassium levels.

Clinicians at participating practices were encouraged to manage all participants’ blood pressure according to NICE guidelines, and the mean results suggested that most participants achieved a reduction in systolic blood pressure across follow-up regardless of the randomization group. Patients in both arms had adjustments made to their blood pressure treatment, and this may have diluted the treatment effect of spironolactone, where some potential reduction in cardiovascular events and mortality would be expected to be achieved through hypertension control.

Recruitment was challenging for several reasons. We found there were fewer eligible patients than anticipated at participating practices, a relatively low response rate to take part in the study among eligible patients and difficulty recruiting additional new practices to the study. The study was designed so that participants would attend 15 study visits across 3 years. People with CKD are typically older with a high burden of multiple long-term conditions and may have been put off volunteering for the study because of concerns around the number of study visits, their own medication burden, the interaction of spironolactone with their current treatment, potential side effects from the drug or issues with mobility and access to healthcare services for follow-up. Future trials could consider using more routinely collected health data to reduce the burden of appointments and demands on study participants in the future to improve recruitment.

Future research could seek to determine if there is a treatment effect from nonsteroidal MRAs, such as finerenone, among a broader population of patients with CKD, such as people without type 2 diabetes or significant albuminuria, to determine whether the positive findings reported in the FIDELITY study could be replicated more widely. Given the high incidence of hyperkalemia associated with spironolactone, other antihypertensive agents, such as ACEi or ARB, may continue to be considered as treatments for blood pressure in preference to MRA in people with CKD. However, one benefit of spironolactone compared to finerenone is its relative effectiveness in reducing blood pressure. A future trial could investigate whether spironolactone could prevent the progression of CKD or reduce vascular events if it were combined with a potassium-binding agent to reduce discontinuation rates due to hyperkalemia^34^.

In conclusion, among participants with stage 3b CKD, treatment with low-dose spironolactone was not associated with a reduction in mortality or cardiovascular events compared to usual care. Discontinuation from the study due to safety concerns was more common among participants randomized to spironolactone. This suggests that low-dose spironolactone should not generally be used in people with stage 3b CKD unless there is another explicit indication for the treatment.

## Methods

### Trial design and participants

BARACK-D was a PROBE trial^34^. The trial design and rationale for this study have been published previously^35^. Trial steering and data monitoring committees supervised the trial. All participants provided written informed consent. The study was approved by a National Health Service (NHS) Research Ethics Committee (REC-13/SC/0114) as well as the Medicines and Healthcare Regulatory Authority (MHRA), relevant NHS Research and Development departments and the host institutions. It was registered prospectively: ISRCTN44522369 (ref. ^36^).

Eligible participants were aged 18 years or older, diagnosed with CKD stage 3b (eGFR = 30–44 ml min^−1^ 1.73 m^−^^2^, but widened to 30–49 ml min^−1^ 1.73 m^−^^2^ following initial recruitment to encompass larger than anticipated measurement error/fluctuations) or with two or more recent eGFR blood tests in their primary care record within this range, with a minimum of 6 weeks between tests. Where only one test had been performed in the 24 months preceding study recruitment and the eGFR was in the 3b range, patients were invited to attend the baseline visit at least 6 weeks from the initial test, at which point the eGFR was repeated to provide a second confirmatory test.

Participants also need to be:

- Willing and able to give informed consent for participation in the study.
- Able (in the recruiting physician’s opinion) and willing to comply with all study requirements.
- Willing to allow his or her GP and consultant, if appropriate, to be notified of their participation in the study.
- Willing to provide contact details to the research team (encompassing the recruitment center and practice staff), for use at any time should the need arise, on trial-related matters.
- Willing to ensure effective contraception during the trial period if they were a female participant of childbearing potential.

Participants were excluded from joining the study if any of the following applied:

- Female participants who were pregnant, lactating or planning pregnancy during the course of the study.
- Type 1 diabetes mellitus.
- Terminal disease or felt otherwise unsuitable by their physician.
- Chronic heart failure clinical diagnosis or known left ventricular systolic dysfunction, defined by an ejection fraction <40%.
- Recent myocardial infarction (within 6 months).
- Active cancer with less than 1-year life expectancy or in palliative care.
- Alcohol or drug abuse.
  - ∘ Suspected or known current hazardous or harmful drinking, as defined by an alcohol intake of greater than 42 units per week.
  - ∘ Suspected or known current substance misuse.
- Most recent potassium results >5.5 mmol l^−1^ where not thought to be spurious, or previously raised potassium needing a reduced dose of ACEI/ARB or intolerance to spironolactone.
- eGFR > 60 ml min^−1^ 1.73 m^−^^2^ in the last 6 months, and no identifiable reason for a temporary reduction in eGFR.
- Serum potassium at baseline over 5 mmol l^−1^.
- Documented Addisonian crisis and/or on fludrocortisone.
- Documented symptomatic hypotension or baseline systolic blood pressure under 100 mm Hg.
- Recent acute kidney injury or admission for renal failure.
- ACR > 70 mg mmol^−1^.
- Prescription of medications with known harmful interactions with spironolactone as documented in the British National Formulary, including tacrolimus, lithium and cyclosporine.
- Any other significant disease or disorder which, in the opinion of the recruiting physician, may either put the participants at risk because of participation in the study or may influence the result of the study or the participant’s ability to participate in the study.

### Baseline visit

Potentially eligible patients were invited to attend a baseline clinic at a trial practice where the trial was explained. Following consent, a baseline assessment was performed to collect key data for the study, including demographics, self-reported gender, medication, comorbidities and quality of life scores. Blood tests were taken for hematology and biochemistry, including renal function and an ECG was performed if available at the study site. The baseline visit was also used to confirm the eligibility of participants.

### Randomization

Eligible participants were randomized in a 1:1 ratio to usual care or usual care plus treatment with spironolactone 25 mg once daily by their usual doctor. Randomization was carried out using Sortition, a validated randomization system developed within our Primary Care Clinical Trials Unit. Participants were enrolled from 329 general practice sites across the UK. We stratified by GP practice to ensure a balance of the two arms within each practice.

### Blinding

BARACK-D was a PROBE trial, in which neither the participants nor their treating healthcare professionals were blinded to their treatment allocation. Advantages of the PROBE design include lower costs and a closer similarity to usual medical care, which is thought to mean such studies provide results that are more directly applicable to routine medical care^34^. The investigators and independent endpoint committee were blinded to the participants’ treatment arm until the completion of the trial.

### Trial procedures

Participants returned for the first study visit 7 days after randomization if they were in the usual care arm, or 7 days after starting spironolactone if not. Subsequent assessment continued for both treatment arms for a further 36 months with follow-up visits at weeks 1, 2, 4, 12 and 26 and then every 13–156 weeks. The list of different measurements taken at each follow-up is described in the study protocol paper^35^. All study visits were conducted at participants’ own general practice sites.

### Monitoring of adverse events

For safety monitoring, adverse events were recorded at trial visits, and any adverse event that was considered related to the study medication as judged by a medically qualified member of the research team or the sponsor was followed up until resolution or until the event was considered stable. Serious adverse events were reported by participating sites to the Clinical Trials Unit within 24 h of discovery or notification of the event. The documentation was then reviewed by a medically qualified member of the trial team, who evaluated the report for causality and expectedness. Any suspected unexpected serious adverse reactions were reported to the competent authorities (MHRA in the UK), the Research Ethics Committee concerned and the host NHS trusts, within the timelines defined in the Medicines for Human Use (Clinical Trials) Regulations, 2004.

Specific ‘stop rules’ were set by the independent Data Monitoring and Ethics Committee (DMEC) and took into consideration the established risks of spironolactone. We measured serum creatinine and potassium at every study visit. If a potassium result was between 5.5 and 5.9 mmol l^−1^, the dose of spironolactone was reduced to 25 mg on alternate days. If the result was between 6.0 and 6.4 mmol l^−1^, spironolactone was withheld for a week and then restarted on alternate days. Spironolactone was discontinued if there was a single potassium result ≥6.5 mmol l^−1^. Participants were also withdrawn from trial treatment if there was a reduction in eGFR of 20% or more between successive visits or 25% or more from baseline, a systolic blood pressure reading <100 mm Hg or a symptomatic postural drop in systolic blood pressure >20 mm Hg. Physicians were strongly encouraged to manage participants’ blood pressure according to the NICE guidelines on CKD and on hypertension, which recommended a target clinic blood pressure <140/90 mm Hg.

In addition, the recruiting physician could discontinue a participant from the study treatment at any time if it was considered necessary for any reason, including the following general rules:

- Ineligibility (either arising during the study or retrospectively having been overlooked at screening).
- Significant protocol deviation as judged by the trial physician.
- Significant noncompliance with treatment regimen or study requirements.
- An adverse event that requires discontinuation of the study medication or results in the inability to continue to comply with study procedures.
- Disease progression that requires discontinuation of the study medication or results in the inability to continue to comply with study procedures.
- Lost to follow-up.

Each participant also had the right to withdraw from the study at any time and for any reason.

### Outcomes

The primary outcome was the time from randomization until the first occurrence of any of the following events: death, hospitalization for heart disease (coronary heart disease, arrhythmia, atrial fibrillation, sudden death or failed sudden death, defined as a cardiac arrest where the participant was successfully resuscitated), stroke, heart failure, transient ischemic attack or PAD, or first onset of any condition listed not present at baseline.

Prespecified secondary outcomes included changes in blood pressure, natriuretic peptides, ACR and eGFR recorded in the primary care record across follow-up, as well as rates of adverse events, including hyperkalemia and hypotension. Hyperkalemia was considered serum potassium of 5.5 mmol l^−1^ or greater, subcategorized as 5.5–5.9 mmol l^−1^, 6.0–6.4 mmol l^−1^ and ≥6.5 mmol l^−1^. Hypotension was defined as systolic blood pressure <100 mm Hg or a drop in the systolic pressure of >20 mm Hg on standing. Progression of kidney disease was defined as a ≥30% increase in creatinine from baseline, a drop of ≥25% in eGFR from baseline or a ≥20% drop in eGFR from the previous result.

An independent panel of three senior clinicians adjudicated each of the endpoints, using all the available clinical information independently of each other, with discussion determining the endpoint where there was initial disagreement.

### Power calculations

We determined that a sample size of 1,511 participants per group (3,022 total) would be required at 80% power to detect a 20% relative risk reduction in death or cardiovascular events over 3 years of follow-up, accounting for an anticipated treatment withdrawal rate of 13%. We chose to power the trial conservatively on a 20% risk reduction because this proposed treatment effect was around half the risk reduction observed in a trial of the aldosterone receptor antagonist, eplerenone, in patients with mild heart failure^37^.

### Statistical analysis

We performed an intention-to-treat analysis, including all participants who had undergone randomization except for those who were found to be ineligible for participation postrandomization. For the primary outcome, we report the HR and 95% CI for the time to the first occurrence of a primary endpoint event using a Cox proportional hazards model adjusted for randomized treatment allocation. The safety analysis included all participants who had actually received the treatment versus the total number of people receiving usual care.

We report the frequency and percentage of the proportion of participants that reached the primary endpoint, the time at risk in days and the incidence rate per 100 years at risk. A Kaplan–Meier curve is used to compare the time to the first occurrence of a primary endpoint event between intervention groups. These analyses are repeated for each individual component of the primary endpoint (hospitalization, CVD and death).

Schoenfeld residuals were conducted for the primary outcome and confirmed that the proportional hazards assumption was not violated.

Natriuretic peptide levels, eGFR, urine ACR and systolic blood pressure were each analyzed by fitting a linear mixed effects model to the data, with the endpoint measure at all available postrandomization follow-up time points as the dependent variable. The model was adjusted for randomized treatment allocation, assessment time point, baseline measurement of the respective variable of interest and interaction between randomized treatment allocation and assessment time point to allow the treatment effect to be estimated at each time point as fixed effects and a random intercept for each participant to account for the repeated measures on the same participant.

The mixed effects model is valid under the missing at random (MAR) assumption; that is, the probability of a value being missing depends on the variables included in the model. The MAR assumption was tested for natriuretic peptide levels, eGFR, urine ACR and systolic blood pressure at 3 years by analyzing each baseline covariate using a logistic regression model to determine which (if any) are associated with missingness. The respective baseline factors found to be associated with missingness were then included in the model as additional fixed effects.

The normality assumptions of the linear mixed effects model were assessed by plotting a histogram of the natriuretic peptide levels, eGFR, urine ACR and systolic blood pressure at each time point split by randomized group, a histogram of the model residuals, an inverse normal plot of the standardized model residuals and a scatter plot of the fitted values versus the model residuals.

We performed a post hoc, on-treatment analysis given the high proportion of participants in the intervention arm who had stopped spironolactone by the end of follow-up and a post hoc analysis of the outcomes censored at 3 years to explore any competing risk effects given the longer than planned mean follow-up. These were intended as exploratory analyses only, to consider whether any benefit might be conferred for participants who are able to tolerate the treatment longer-term and determine any medium-term treatment effects.

The trial failed to recruit to target. We therefore also performed a post hoc conditional power calculation to inform the likelihood of finding a significant association between spironolactone treatment and our primary outcome had the trial achieved its recruitment goal. To calculate conditional power, the remaining data for the number of participants that we had planned to recruit were simulated first based on the predicted event rates used to inform the original power calculation and then using the observed event rates from our study.

### Within-trial economic analysis

The primary economic evaluation took the form of a cost-utility analysis (CUA), expressed in terms of incremental cost per QALY gained. The time horizon covered the period from randomization to the end of follow-up at 156 weeks (3 years) postrandomization. All costs (derived from the 2019/2020 National Cost Collection for the NHS and using methods in the 2022 NICE Health Technology Evaluation Manual) and health outcomes were discounted at an annual rate of 3.5%^38^.

The EQ-5D-5L was used to measure patient HRQoL at baseline, 6 months, 12 months, 2 years and 3 years. This was converted to a utility score of between 0 (dead) and 1 (perfect health) to inform the CUA. The KDQOL-SF score was also captured at the same time points to provide a measure of quality of life specific to kidney disease.

Summary statistics were generated for resource use costs by time point and treatment group. Statistics generated using (1) all available data and (2) participants with complete data over follow-up time points were presented separately. Between-group differences in resource use costs at each time point were compared using the two-sample *t* test. Statistical significance was assessed at the 5% significance level. The bootstrap 95% CI, calculated from 1,000 bootstrap replications, for the between-group differences in mean resource use and cost estimates were reported.

### Protocol deviations

In addition to the failure to recruit the intended number of participants and the post hoc analyses outlined below, the other deviations from the original study protocol were to remove two patient questionnaires for assessing well-being and quality of life (the ICEpop Capability Measure for Adults (ICECAP-A) and Visual Analogue Scale - Quality of Life (VAS QL)) and to reduce the frequency of administering healthcare resource use diary cards from every 13 weeks throughout follow-up to annually after the first year of the study. Both steps were taken to reduce the amount of time participants needed to spend engaging in the study. We do not feel that these changes impact our summary findings.

It was prespecified in the statistical analysis plan that all models in the primary and secondary analyses would be adjusted for GP practice as a random effect. Participants were randomized from over 300 GP practices across the UK. All of the practices recruited a small number of participants, and several only recruited one or two participants to trial. Due to the large number of practices, many of the models struggled to handle being adjusted for practice, so it was decided to not adjust any of the analyses for GP practice. Due to the large number of recruiting GP practices, this information is also not presented in Table 1.

It was originally planned in the statistical analysis plan that the primary analysis would be conducted using a mixed-effect Cox proportional hazards model adjusting for randomized treatment allocation as a fixed effect and GP practice as a random effect. As described above, it was decided that due to the amount of GP practices, the models would not adjust for GP practice; as such, the primary analysis was conducted using a Cox proportional hazards model, which was adjusted for randomized treatment allocation as a covariate only.

The primary outcome of the study in the original protocol, statistical analysis plan and endpoint forms included PAD. However, this was unintentionally missed from the primary outcome table in a subsequent updated protocol. This was reported to the DMEC, Trial Steering Committee and the funder before being corrected, before the data lock and before any analysis had occurred. We report a sensitivity analysis excluding PAD from the primary composite outcome to confirm that this correction did not impact our summary results.

### Reporting summary

Further information on research design is available in the Nature Portfolio Reporting Summary linked to this article.

## Online content

Any methods, additional references, Nature Portfolio reporting summaries, source data, extended data, supplementary information, acknowledgements, peer review information; details of author contributions and competing interests; and statements of data and code availability are available at 10.1038/s41591-024-03263-5.

## Supplementary information

Supplementary Information Supplementary Tables 1 and 2, study protocol and statistical analysis plan.

Reporting Summary

## Figures and Tables

**Extended Data Table 1:**
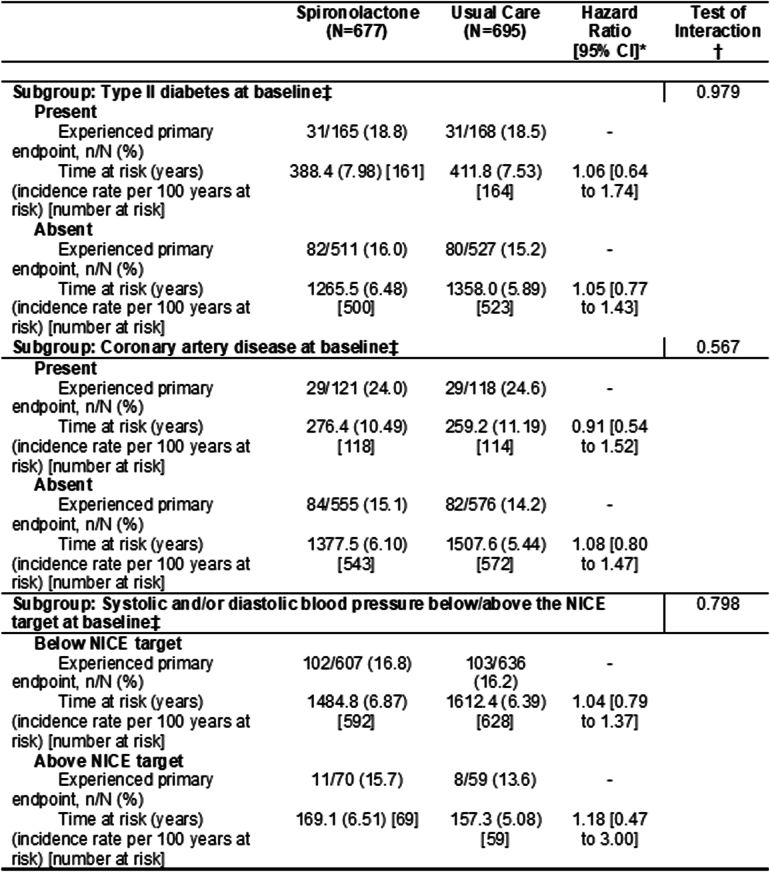
Summary statistics and hazard ratios for the subgroup analyses among people with type 2 diabetes, coronary artery disease or blood pressure values above or below the NICE target at baseline

*Spironolactone versus usual care. ^†^Two-sided P-value. Level of significance = 0.05. ^‡^Cox proportional hazards model adjusted for randomized arm, an indicator variable for the subgroup, and an interaction between randomized arm and the subgroup indicator variable. A hazard ratio of less than 1 indicates improvement in favor of spironolactone. All row and column headings are in bold format for clarity.

**Extended Data Table 2:**
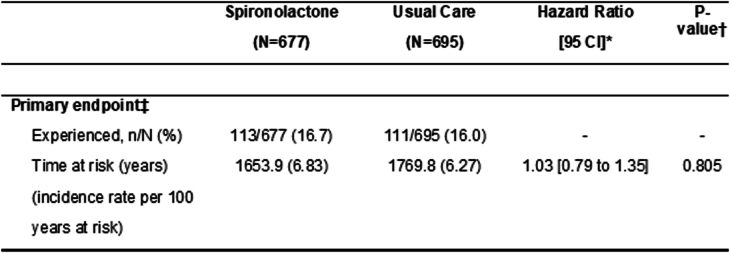
Summary statistics and the hazard ratio for the sensitivity analysis, including an indicator variable for type 2 diabetes, coronary artery disease or blood pressure values above or below the NICE target at baseline as an additional covariate in the model

*Spironolactone versus usual care. ^†^Two-sided P-value. Level of significance = 0.05. ^‡^Cox proportional hazards model adjusted for randomized arm, type II diabetes at baseline, coronary artery disease at baseline and blood pressure below or above NICE target at baseline. A hazard ratio of less than 1 indicates improvement in favor of spironolactone. All row and column headings are in bold format for clarity.

**Extended Data Table 3:**
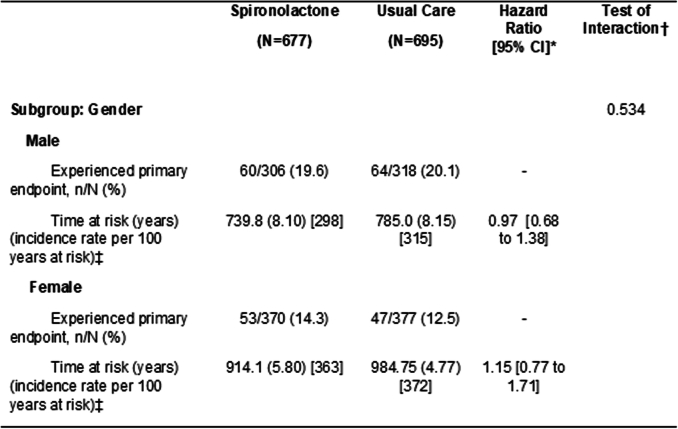
Post hoc sub-group analysis of the primary outcome in relation to gender

*Spironolactone versus usual care. ^†^Two-sided P-value. Level of significance = 0.05. ^‡^Primary endpoint. Cox proportional hazards model adjusted for randomized arm. A hazard ratio of less than 1 indicates improvement in favor of spironolactone. All row and column headings are in bold format for clarity.

**Extended Data Table 4:**
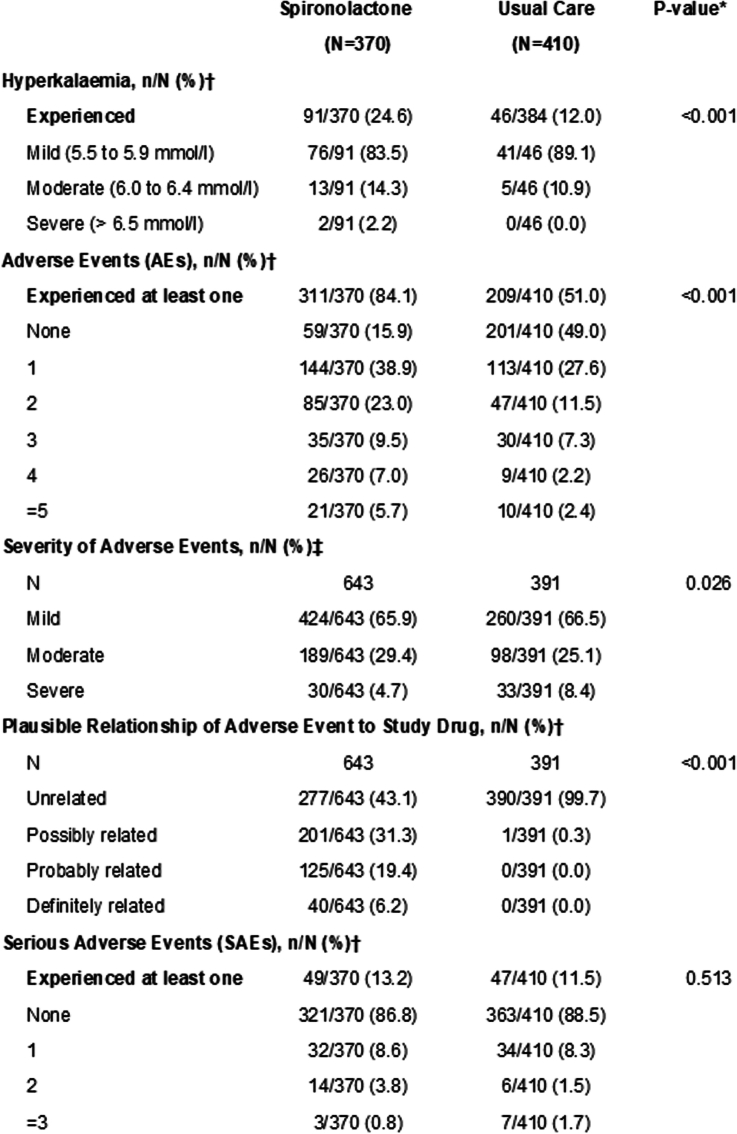
Post hoc, on-treatment safety analysis showing adverse events in women

*Spironolactone versus usual care. Two-sided P-value. Level of significance = 0.05. ^†^Fisher’s exact test. ^‡^Chi-squared test. All row and column headings are in bold format for clarity.

**Extended Data Table 5:**
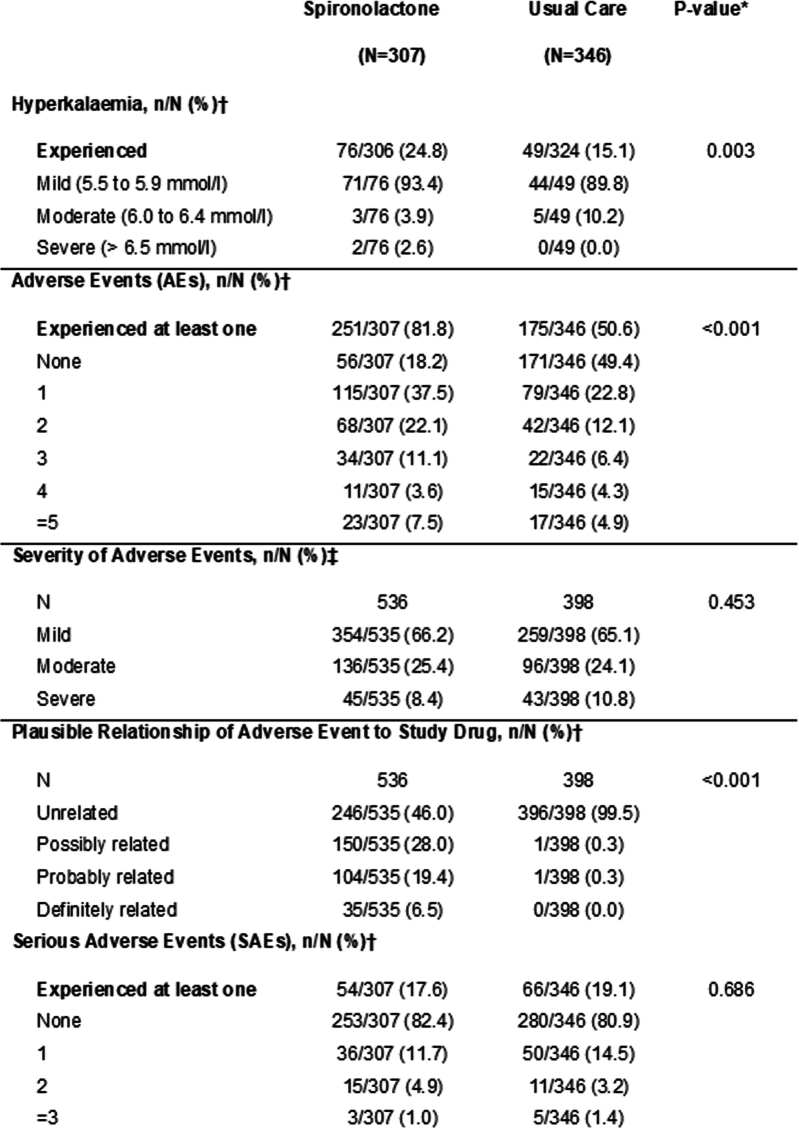
Post hoc, on-treatment safety analysis showing adverse events in men

*Spironolactone versus usual care. Two-sided P-value. Level of significance = 0.05. ^†^Fisher’s exact test. ^‡^Chi-squared test. All row and column headings are in bold format for clarity.

## Data Availability

All data are securely stored under the Data Protection Act 2004 and adhere to the Primary Care Clinical Trials Unit data-sharing standard operating procedure in which data-sharing agreements have to be approved by both the Trial Management Group and the sponsor. All available data can be obtained by contacting the chief investigator (F.D.R.H.). Individual patient data will be shared in datasets in a de-identified and anonymized format, following our data-sharing process. We will aim to make data available within 6–9 weeks. We are working toward having a fully de-identified dataset available to publicly share, but this has not been completed at the time of publication.

## Code availability

The scripts for the statistical analysis are freely available at https://github.com/samjamesmort/BARACK-D_Statistical_Analysis.

## References

[1] Keith, D. S. et al. Longitudinal follow-up and outcomes among a population with chronic kidney disease in a large managed care organization. *Arch. Intern. Med.* **164**, 659–663 (2004). 15037495 10.1001/archinte.164.6.659

[2] Go, A. S. et al. Chronic kidney disease and the risks of death, cardiovascular events, and hospitalization. *N. Engl. J. Med.* **351**, 1296–1305 (2004). 15385656 10.1056/NEJMoa041031

[3] Alicic, R. Z., Rooney, M. T. & Tuttle, K. R. Diabetic kidney disease: challenges, progress, and possibilities. *Clin. J. Am. Soc. Nephrol.* **12**, 2032–2045 (2017). 28522654 10.2215/CJN.11491116 PMC5718284

[4] Tonelli, M. et al. Chronic kidney disease and mortality risk: a systematic review. *J. Am. Soc. Nephrol.* **17**, 2034–2047 (2006). 16738019 10.1681/ASN.2005101085

[5] Jankowski, J. et al. Cardiovascular disease in chronic kidney disease: pathophysiological insights and therapeutic options. *Circulation* **143**, 1157–1172 (2021). 33720773 10.1161/CIRCULATIONAHA.120.050686 PMC7969169

[6] Foley, R. N. et al. Left ventricular hypertrophy in new hemodialysis patients without symptomatic cardiac disease. *Clin. J. Am. Soc. Nephrol.* **5**, 805–813 (2010). 20378644 10.2215/CJN.07761109 PMC2863966

[7] Foley, R. N. et al. Chronic kidney disease and the risk for cardiovascular disease, renal replacement, and death in the United States Medicare population, 1998 to 1999. *J. Am. Soc. Nephrol.* **16**, 489–495 (2005). 15590763 10.1681/ASN.2004030203

[8] Fox, C. S. et al. Associations of kidney disease measures with mortality and end-stage renal disease in individuals with and without diabetes: a meta-analysis. *Lancet* **380**, 1662–1673 (2012). 23013602 10.1016/S0140-6736(12)61350-6 PMC3771350

[9] Chronic Kidney Disease Prognosis, C. et al. Association of estimated glomerular filtration rate and albuminuria with all-cause and cardiovascular mortality in general population cohorts: a collaborative meta-analysis. *Lancet* **375**, 2073–2081 (2010). 20483451 10.1016/S0140-6736(10)60674-5 PMC3993088

[10] Van Biesen, W. et al. The glomerular filtration rate in an apparently healthy population and its relation with cardiovascular mortality during 10 years. *Eur. Heart J.* **28**, 478–483 (2007). 17223665 10.1093/eurheartj/ehl455

[11] Abramson, J. L. et al. Chronic kidney disease, anemia, and incident stroke in a middle-aged, community-based population: the ARIC study. *Kidney Int.* **64**, 610–615 (2003). 12846757 10.1046/j.1523-1755.2003.00109.x

[12] GBD Chronic Kidney Disease Collaboration Global, regional, and national burden of chronic kidney disease, 1990–2017: a systematic analysis for the Global Burden of Disease Study 2017. *Lancet* **395**, 709–733 (2020). 32061315 10.1016/S0140-6736(20)30045-3 PMC7049905

[13] Hill, N. R. et al. Global prevalence of chronic kidney disease—a systematic review and meta-analysis. *PLoS ONE* **11**, e0158765 (2016). 27383068 10.1371/journal.pone.0158765 PMC4934905

[14] Vidal-Petiot, E. et al. Chronic kidney disease has a graded association with death and cardiovascular outcomes in stable coronary artery disease: an analysis of 21,911 patients from the CLARIFY registry. *J. Clin. Med.* **9**, 4 (2019). 31861379 10.3390/jcm9010004 PMC7019870

[15] Di Angelantonio, E. et al. Chronic kidney disease and risk of major cardiovascular disease and non-vascular mortality: prospective population based cohort study. *BMJ* **341**, c4986 (2010). 20884698 10.1136/bmj.c4986 PMC2948649

[16] Levin, A. et al. Global kidney health 2017 and beyond: a roadmap for closing gaps in care, research, and policy. *Lancet* **390**, 1888–1917 (2017). 28434650 10.1016/S0140-6736(17)30788-2

[17] Brenner, B. M. et al. Effects of losartan on renal and cardiovascular outcomes in patients with type 2 diabetes and nephropathy. *N. Engl. J. Med.* **345**, 861–869 (2001). 11565518 10.1056/NEJMoa011161

[18] Lewis, E. J. et al. Renoprotective effect of the angiotensin-receptor antagonist irbesartan in patients with nephropathy due to type 2 diabetes. *N. Engl. J. Med.* **345**, 851–860 (2001). 11565517 10.1056/NEJMoa011303

[19] Sarafidis, P. et al. Mineralocorticoid receptor antagonist use in chronic kidney disease with type 2 diabetes: a clinical practice document by the European Renal Best Practice (ERBP) board of the European Renal Association (ERA). *Clin. Kidney J.* **16**, 1885–1907 (2023). 37915899 10.1093/ckj/sfad139 PMC10616462

[20] Edwards, N. C. et al. Effect of spironolactone on left ventricular mass and aortic stiffness in early-stage chronic kidney disease: a randomized controlled trial. *J. Am. Coll. Cardiol.* **54**, 505–512 (2009). 19643310 10.1016/j.jacc.2009.03.066

[21] Edwards, N. C. et al. Effect of spironolactone on left ventricular systolic and diastolic function in patients with early stage chronic kidney disease. *Am. J. Cardiol.* **106**, 1505–1511 (2010). 21059444 10.1016/j.amjcard.2010.07.018

[22] Bakris, G. L. et al. Effect of finerenone on chronic kidney disease outcomes in type 2 diabetes. *N. Engl. J. Med.* **383**, 2219–2229 (2020). 33264825 10.1056/NEJMoa2025845

[23] Pitt, B. et al. Cardiovascular events with finerenone in kidney disease and type 2 diabetes. *N. Engl. J. Med.* **385**, 2252–2263 (2021). 34449181 10.1056/NEJMoa2110956

[24] Agarwal, R. et al. Cardiovascular and kidney outcomes with finerenone in patients with type 2 diabetes and chronic kidney disease: the FIDELITY pooled analysis. *Eur. Heart J.* **43**, 474–484 (2022). 35023547 10.1093/eurheartj/ehab777 PMC8830527

[25] Agarwal, R. et al. Steroidal and non-steroidal mineralocorticoid receptor antagonists in cardiorenal medicine. *Eur. Heart J.* **42**, 152–161 (2021). 33099609 10.1093/eurheartj/ehaa736 PMC7813624

[26] Baigent, C. et al. The effects of lowering LDL cholesterol with simvastatin plus ezetimibe in patients with chronic kidney disease (Study of Heart and Renal Protection): a randomised placebo-controlled trial. *Lancet* **377**, 2181–2192 (2011). 21663949 10.1016/S0140-6736(11)60739-3 PMC3145073

[27] Grune, J. et al. Selective mineralocorticoid receptor cofactor modulation as molecular basis for finerenone’s antifibrotic activity. *Hypertension* **71**, 599–608 (2018). 29437893 10.1161/HYPERTENSIONAHA.117.10360

[28] Heerspink, H. J. L. et al. Dapagliflozin in patients with chronic kidney disease. *N. Engl. J. Med.* **383**, 1436–1446 (2020). 32970396 10.1056/NEJMoa2024816

[29] Perkovic, V. et al. Canagliflozin and renal outcomes in type 2 diabetes and nephropathy. *N. Engl. J. Med.* **380**, 2295–2306 (2019). 30990260 10.1056/NEJMoa1811744

[30] The EMPA-KIDNEY Collaborative Group & Herrington, W. G. et al. Empagliflozin in patients with chronic kidney disease. *N. Engl. J. Med.* **388**, 117–127 (2023). 36331190 10.1056/NEJMoa2204233 PMC7614055

[31] National Institute for Health and Care Excellence. Dapagliflozin for treating chronic kidney disease. Technology appraisal guidance (TA775). www.nice.org.uk/guidance/ta775 (2022). 40455906

[32] Hinton, W. et al. Prescribing sodium–glucose co-transporter-2 inhibitors for type 2 diabetes in primary care: influence of renal function and heart failure diagnosis. *Cardiovasc. Diabetol.* **20**, 130 (2021). 34183018 10.1186/s12933-021-01316-4 PMC8237469

[33] Agarwal, R. et al. Patiromer versus placebo to enable spironolactone use in patients with resistant hypertension and chronic kidney disease (AMBER): a phase 2, randomised, double-blind, placebo-controlled trial. *Lancet* **394**, 1540–1550 (2019). 31533906 10.1016/S0140-6736(19)32135-X

[34] Hansson, L., Hedner, T. & Dahlof, B. Prospective randomized open blinded end-point (PROBE) study. A novel design for intervention trials. *Blood Press.* **1**, 113–119 (1992). 1366259 10.3109/08037059209077502

[35] Hill, N. R. et al. Benefits of Aldosterone Receptor Antagonism in Chronic Kidney Disease (BARACK D) trial—a multi-centre, prospective, randomised, open, blinded end-point, 36-month study of 2,616 patients within primary care with stage 3b chronic kidney disease to compare the efficacy of spironolactone 25 mg once daily in addition to routine care on mortality and cardiovascular outcomes versus routine care alone: study protocol for a randomized controlled trial. *Trials* **15**, 160 (2014). 24886488 10.1186/1745-6215-15-160 PMC4113231

[36] ISRCTN Registry. Benefits of Aldosterone Receptor Antagonism in Chronic Kidney Disease (BARACK-D) trial: a potential new treatment for kidney disease. www.isrctn.com/ISRCTN44522369?q=ISRCTN44522369&filters=&sort=&offset=1&totalResults=1&page=1&pageSize=10 (2013).

[37] Pitt, B. et al. Eplerenone, a selective aldosterone blocker, in patients with left ventricular dysfunction after myocardial infarction. *N. Engl. J. Med.* **348**, 1309–1321 (2003). 12668699 10.1056/NEJMoa030207

[38] National Institute for Health and Care Excellence. NICE health technology evaluations: the manual (PMG36).www.nice.org.uk/process/pmg36/chapter/introduction-to-health-technology-evaluation (2022).

