# Supplementary material for: Low-dose spironolactone and cardiovascular outcomes in moderate stage chronic kidney disease: a randomized controlled trial
Source: Nat Med. 2024 Sep 30;30(12):3634–45. doi: 10.1038/s41591-024-03263-5 (PMC11753262; doi:10.1038/s41591-024-03263-5)

nature medicine

Supplementary information https://doi.org/10.1038/s41591-024-03263-5

Low-dose spironolactone and cardiovascular outcomes in moderate stage chronic kidney disease: a randomized controlled trial

In the format provided by the authors and unedited

Supplementary material

Supplementary Table 1. Adverse events coded by MedDRA system organ class

Number of participants (%) number of events	Spironolactone (N=388)	Standard Care (N=984)	Overall (N=1372)
**SYSTEM ORGAN CLASS**			
Blood and lymphatic system disorders	3 (0.8) 3	3 (0.3) 4	6 (0.4) 7
Cardiac disorders	17 (4.4) 21	47 (4.8) 61	64 (4.7) 82
Congenital, familial and genetic disorders	0 (0.0) 0	1 (0.1) 1	1 (0.1) 1
Ear and labyrinth disorders	1 (0.3) 1	5 (0.5) 5	6 (0.4) 6
Endocrine disorders	0 (0.0) 0	2 (0.2) 2	2 (0.1) 2
Eye disorders	6 (1.5) 6	9 (0.9) 10	15 (1.1) 16
Gastrointestinal disorders	34 (8.8) 59	80 (8.1) 137	114 (8.3) 196
General disorders and administration site conditions	34 (8.8) 47	62 (6.3) 66	96 (7.0) 113
Hepatobiliary disorders	2 (0.5) 2	3 (0.3) 6	5 (0.4) 8
Immune system disorders	0 (0.0) 0	2 (0.2) 3	2 (0.1) 3
Infections and infestations	41 (10.6) 48	80 (8.1) 101	121 (8.8) 149
Injury, poisoning and procedural complications	16 (4.1) 28	37 (3.8) 46	53 (3.9) 74
Investigations	229 (59.0) 346	407 (41.4) 591	636 (46.4) 937
Metabolism and nutrition disorders	30 (7.7) 38	37 (3.8) 42	67 (4.9) 80
Musculoskeletal and connective tissue disorders	32 (8.2) 41	52 (5.3) 73	84 (6.1) 114
Neoplasms benign, malignant and unspecified (incl cysts and polyps)	15 (3.9) 25	31 (3.2) 33	46 (3.4) 58
Nervous system disorders	41 (10.6) 57	76 (7.7) 96	117 (8.5) 153
Psychiatric disorders	4 (1.0) 4	8 (0.8) 10	12 (0.9) 14
Renal and urinary disorders	23 (5.9) 25	47 (4.8) 58	70 (5.1) 83
Reproductive system and breast disorders	25 (6.4) 29	7 (0.7) 9	32 (2.3) 38

Number of participants (%) number of events	**Spironolactone**<br><br>**(N=388)**	**Standard Care**<br><br>**(N=984)**	**Overall**<br><br>**(N=1372)**
Respiratory, thoracic and mediastinal disorders	14 (3.6) 17	33 (3.4) 44	47 (3.4) 61
Skin and subcutaneous tissue disorders	8 (2.1) 9	12 (1.2) 13	20 (1.5) 22
Social circumstances	0 (0.0) 0	1 (0.1) 1	1 (0.1) 1
Surgical and medical procedures	21 (5.4) 23	38 (3.9) 43	59 (4.3) 66
Vascular disorders	33 (8.5) 37	65 (6.6) 75	98 (7.1) 112
Missing	0 (0.0) 0	1 (0.1) 1	1 (0.1) 1

Supplementary Table 2. Summary statistics and the hazard ratio for the post hoc analysis based on a per-protocol principle and an on-treatment population

	Spironolactone (N=677)	Standard Care (N=695)	Hazard Ratio [95 CI]†	P-value‡
**POST HOC ANALYSIS**				
**Per-protocol principle and an on-treatment population: Primary endpoint[1]**				
Experienced, n/N (%)	55/331 (16.6)	93/576 (16.1)	-	-
Time at risk (years) (incidence rate per 100 years at risk) [number at risk]	919.0 (5.98) [331]	1575.1 (5.90) [576]	1.01 [0.72 to 1.41]	0.946
**Excluding PAD: Primary endpoint[1]**				
Experienced, n/N (%)	94/677 (13.9)	96/695 (13.8)	-	-
Time at risk (years) (incidence rate per 100 years at risk)* [number at risk]	1679.5 (5.60) [661]	1786.5 (5.37) [687]	0.99 [0.75 to 1.33]	0.973

†Spironolactone versus standard care. [1]Cox-proportional hazards model adjusted for randomised arm. ‡Level of significance = 0.05.

Trial Title: Benefits of Aldosterone Receptor Antagonism in Chronic Kidney Disease (BARACK D) Trial

Internal Reference Number / Short title: BARACK-D (RH/BARACK D/0003)

Ethics Ref: 13/SC/0114

IRAS Project ID: 107072

EudraCT Number: 2012-002672-13

Date and Version No: V8.0 11 Aug 2022

Chief Investigator:

Professor Richard Hobbs
Nuffield Department of Primary Health Care Sciences
Radcliffe Primary Care
Radcliffe Observatory Quarter,
Woodstock Road,
Oxford OX2 6GG

Investigators:

Prof Richard McManus, University of Oxford
Dr Jonathan Townend, University of Birmingham
Dr Charles Ferro, University of Birmingham
Prof Peter Bower, University of Manchester
Prof Daniel Lasserson, University of Birmingham
Prof Andrew Farmer, University of Oxford
Prof David Fitzmaurice, University of Warwick
Prof Gene Feder, University of Bristol
Prof Paul Little, University of Southampton
Dr Nadeem Qureshi, University of Nottingham
Dr Rafael Perera-Salazar, University of Oxford
Dr Jane Wolstenholme, University of Oxford
Dr Emma Ogburn, University of Oxford
Dr Louise Jones, University of Oxford

Sponsor:

University of Oxford
Research Governance, Ethics & Assurance Team
Joint Research office
1st Floor, Boundary Brook House,
Churchill Drive,
Headington,
Oxford OX3 7GB

© Copyright: The University of Oxford and Oxford University Hospitals NHS Foundation Trust 2019

Funder: NIHR Health Technology Assessment Programme

Chief Investigator Signature: The approved protocol should be signed by author(s) and/or person(s) authorised to sign the protocol

Statistician Signature:

Confidentiality Statement

This document contains confidential information that must not be disclosed to anyone other than the Sponsor, the Investigator Team, HRA, host organisation, and members of the Research Ethics Committee and Regulatory Authorities unless authorised to do so.

© Copyright: The University of Oxford and Oxford University Hospitals NHS Foundation Trust 2019

Trial Title: Benefits of Aldosterone Receptor Antagonism in Chronic Kidney Disease (BARACK D) Trial
EudraCT Number: 2012-002672-13
Protocol Date and Version No: Version 8.0, 11 Aug 2022

Protocol signature page

The undersigned has read and understood the trial protocol detailed above and agrees to conduct the trial in compliance with the protocol.

______________________	______________________	______________________	______________
**Principal Investigator**<br>(Please print name)	**Signature**	**Site name or ID number**	**Date**

Following any amendments to the protocol, this page must be updated with the new protocol version number and date and re-signed by the site PI.

© Copyright: The University of Oxford and Oxford University Hospitals NHS Foundation Trust 2019

Table of Contents

1. KEY TRIAL CONTACTS 7
2. LAY SUMMARY 8
3. SYNOPSIS 8
4. ABBREVIATIONS 11
5. BACKGROUND AND RATIONALE 14
   5.1. Rationale for Current Trial 17
6. OBJECTIVES AND OUTCOME MEASURES 19
7. TRIAL DESIGN 21
   7.1. Summary of Trial Design 21
   7.2. Primary and Secondary Endpoints/Outcome Measures 21
8. PARTICIPANT IDENTIFICATION 21
   8.1. Trial Participants 21
   8.2. Exclusion Criteria 22
   8.3. Expenses and Benefits 23
9. TRIAL PROCEDURES 23
   9.1. Recruitment, Screening and Eligibility Assessment 23
   9.2. Informed Consent 24
   9.3. Randomisation 24
   9.4. Blinding and code-breaking 24
   9.5. Baseline Assessments 25
   9.6. Subsequent Visits 26
   9.7. Sample Handling 27
   9.8. Early Discontinuation/Withdrawal of Participants 27
   9.9. Definition of End of Trial 28
10. TRIAL INTERVENTIONS 28
   10.1. Investigational Medicinal Product(s) (IMP) Description 28
   10.2. Other Treatments (non-IMPS) 29
   10.3. Other Interventions 29
11. SAFETY REPORTING 29
   11.1. Adverse Event Definitions 29
   11.2. Assessment results outside of normal parameters as AEs and SAEs 31
   11.3. Assessment of Causality 31

© Copyright: The University of Oxford and Oxford University Hospitals NHS Foundation Trust 2019

11.4. Procedures for Reporting Adverse Events 31
11.5. Reporting Procedures for Serious Adverse Events 32
11.6. Expectedness 33
11.7. SUSAR Reporting 33
11.8. Development Safety Update Reports 34
12. STATISTICS 34
12.1. Statistical Analysis Plan (SAP) 34
12.2. Description of Statistical Methods 35
12.3. Sample Size Determination 35
12.4. Analysis Populations 36
12.5. Decision Points 36
12.6. Stopping Rules 36
12.7. The Level of Statistical Significance 36
12.8. Procedure for Accounting for Missing, Unused, and Spurious Data. 36
12.9. Procedures for Reporting any Deviation(s) from the Original Statistical Plan 37
12.10. Health Economics Analysis 37
13. DATA MANAGEMENT 39
13.1. Source Data 39
13.2. Access to Data 40
13.3. Data Recording and Record Keeping 40
14. QUALITY ASSURANCE PROCEDURES 41
14.1. Risk assessment 41
14.2. Monitoring 41
14.3. Trial committees 41
15. PROTOCOL DEVIATIONS 42
16. SERIOUS BREACHES 42
17. ETHICAL AND REGULATORY CONSIDERATIONS 43
17.1. Declaration of Helsinki 43
17.2. Guidelines for Good Clinical Practice 43
17.3. Approvals 43
17.4. Other Ethical Considerations 43
17.5. Reporting 43
17.6. Transparency in Research 43
17.7. Participant Confidentiality 43
17.8. Expenses and Benefits 44

© Copyright: The University of Oxford and Oxford University Hospitals NHS Foundation Trust 2019

18. FINANCE AND INSURANCE 44
18.1. Funding 44
18.2. Insurance 44
18.3. Contractual arrangements 44
19. PUBLICATION POLICY 44
20. DEVELOPMENT OF A NEW PRODUCT/ PROCESS OR THE GENERATION OF INTELLECTUAL PROPERTY 45
21. ARCHIVING 45
22. REFERENCES 45
23. APPENDIX A: TRIAL FLOW CHART 51
24. APPENDIX B: SCHEDULE OF PROCEDURES 52
25. APPENDIX C: SAE REPORTING FLOW CHART 54
26. APPENDIX D: AMENDMENT HISTORY 55
27. APPENDIX E: TRANSPARENCY STATEMENT FROM THE SPONSOR 58

© Copyright: The University of Oxford and Oxford University Hospitals NHS Foundation Trust 2019

1. KEY TRIAL CONTACTS

**Chief Investigator**	Professor Richard Hobbs<br>Nuffield Department of Primary Health Care Sciences<br>Radcliffe Primary Care<br>Radcliffe Observatory Quarter,<br>Woodstock Road,<br>Oxford. OX2 6GG<br>Email: Richard.hobbs@phc.ox.ac.uk
**Sponsor**	University of Oxford<br>Research Governance, Ethics &<br>Assurance Team,<br>Joint Research Office, 1st floor, Boundary Brook House, Churchill Drive,<br>Headington,<br>Oxford OX3 7GB<br>Email: ctrg@admin.ox.ac.uk
**Funder(s)**	NIHR Health Technology Assessment Programme
**Clinical Trials Unit**	Primary Care Clinical trials Unit,<br>Nuffield Department of Primary Care Health Sciences,<br>University of Oxford,<br>Gibson Building,<br>Radcliffe Observatory Quarter,<br>Woodstock Road,<br>Oxford OX2 6GG<br>email: barack@phc.ox.ac.uk<br>Tel:+ 44(0)1865 287849<br>Fax: +44(0)1865 289287
**Statistician**	Professor Ly-Mee Yu<br>Nuffield Department of Primary Care Health Sciences,<br>University of Oxford,<br>Gibson Building,<br>Radcliffe Observatory Quarter,<br>Woodstock Road,<br>Oxford OX2 6GG<br>Email: ly-mee.yu@phc.ox.ac.uk<br>Tel: +44(0)1865 617199<br>Fax:+44(0)1865 289287
**Committees**	

© Copyright: The University of Oxford and Oxford University Hospitals NHS Foundation Trust 2019

2. LAY SUMMARY

Chronic Kidney Disease (CKD) affects around 10% of the UK population. It is linked with increasing age and is more common in people with other illnesses such as hypertension, diabetes mellitus, obesity and primary renal disorders. Of interest to this study, CKD is a major contributor to cardiovascular disease, with CKD patients showing greater incidence of heart failure and sudden cardiac death. Conventional treatments for cardiovascular disease have been disappointing in CKD patients. There are also limited treatment options to prevent further decline in kidney function. Established drugs called aldosterone receptor antagonists reduce deaths in patients with heart disease. There is also evidence that these drugs may reduce kidney damage attributed to circulating aldosterone. In order to answer the research question, we will conduct a prospective randomised open blinded endpoint (PROBE) trial using a low dose of the aldosterone receptor antagonist, spironolactone.

3. SYNOPSIS

Trial Title	Benefits of Aldosterone Receptor Antagonism in Chronic Kidney Disease (BARACK D) Trial: a prospective randomised open blinded endpoint trial to determine the effect of aldosterone receptor antagonism on mortality and cardiovascular outcomes in patients with stage 3b chronic kidney disease.
Internal ref. no. (or short title)	A potential new treatment for kidney disease<br>RH/BARACK D/0003
Trial registration	ISRCTN44522369
Sponsor	University of Oxford<br>Research Governance<br>Ethics & Assurance Team<br>Boundary Brook House, Churchill Drive,<br>Headington,<br>Oxford OX3 7GB
Funder	NIHR Health Technology Assessment Programme
Clinical Phase	III
Trial Design	Prospective Randomised Open Blinded Endpoint (PROBE)
Trial Participants	Patients meeting the criteria for a diagnosis of CKD stage 3b (eGFR 30-44 ml/min/1.73m^2) according to NICE guidelines. Due to the higher than anticipated measurement error/fluctuations, the range was extended to 30-50 ml/min/1.73m^2 following the initial recruitment period.
Sample Size	3022 participants will be randomised 1:1 to receive either routine care or the aldosterone receptor antagonist spironolactone 25mg OD on top of routine care.
Planned Trial Period	97 months (from first randomisation to last active patient follow-up, excluding long term mortality follow-up).

© Copyright: The University of Oxford and Oxford University Hospitals NHS Foundation Trust 2019

	Participant involvement: 3 years (active participation), followed by long term follow-up via medical records to Dec 2049.		
Planned Recruitment period	Start date: November 2013<br>Planned recruitment end date: August 2018		
	Objectives	Outcome Measures	Timepoint(s)
Primary	To determine the effect of aldosterone receptor antagonism on mortality and cardiovascular outcomes (onset or progression of cardiovascular disease) in patients with stage 3b CKD.	Time from randomisation until the first occurring of:<br>• Death or<br>• Hospitalisation for heart disease (coronary heart disease, arrhythmia, atrial fibrillation, sudden death, failed sudden death), stroke, transient ischaemic attack, peripheral arterial disease or heart failure or<br>• First onset of any condition listed above not present at baseline.<br>Primary endpoints will be adjudicated by an independent endpoints committee blinded to treatment arm.	Time from randomisation to first occurrence
Primary Long term	To determine the effect of aldosterone receptor antagonism (even short-term use) on long term mortality and cardiovascular outcome in patients with stage 3b CKD.	Annual rates of:<br>• Death,<br>• Hospitalisation for heart disease (coronary heart disease, arrhythmia, atrial fibrillation, suddendeath, resuscitated sudden death), stroke, transient ischaemic attack, peripheral arterial disease or heart failure.<br>• First onset of any condition listed above not present at baseline.	Annual rates, collected via medical notes review

© Copyright: The University of Oxford and Oxford University Hospitals NHS Foundation Trust 2019

Secondary	To determine the effect of adding an aldosterone receptor antagonism in patients on 1-5 below:<br><br>1. The individual components of the composite primary outcome	• Hospitalisation or new onset heart disease (coronary heart disease, arrhythmia, atrial fibrillation, suddendeath, resuscitated sudden death), stroke, transient ischaemic attack, peripheral arterial disease or heart failure.	• Total occurrences
	2. Measures of cardiovascular haemodynamics	• Change in blood pressure annually and at final visit.	• Annually and at final visit.
	3. Measures of renal function	• Changes in NP<br>• Change in ACR<br>• Changes in eGFR	• Change from baseline, annually and to final visit for NP, ACR and eGFR
	4. Healthcare cost evaluation	• Change in health status on EQ-5D-5L, KDQol, (ICECAP-A and Qol VAS – Oxford only) and NHS resource use (records).	• Change from baseline, annually and to final visit
	5. Safety	• Rates of hypotension (<100mmHg systolic or >20 mmHg systolic drop on standing)<br>• Rates of adverse events<br>• Rates of hyperkalaemia	• Total occurrences<br><br>• Total occurrences
Intervention(s)<br><br>IMP(s)	Spironolactone<br><br>Tablet, 25 mg OD, Oral in addition to routine care		
Comparator	Routine care		

© Copyright: The University of Oxford and Oxford University Hospitals NHS Foundation Trust 2019

4. ABBREVIATIONS

AE	Adverse event
AR	Adverse reaction
CI	Chief Investigator
CRA	Clinical Research Associate (Monitor)
CRF	Case Report Form
CRO	Contract Research Organisation
CT	Clinical Trials
CTA	Clinical Trials Authorisation
CTRG	Clinical Trials and Research Governance
DMEC/DMSC	Data Monitoring Ethics Committee / Data Monitoring and Safety Committee
DSUR	Development Safety Update Report
GCP	Good Clinical Practice
GP	General Practitioner
GTAC	Gene Therapy Advisory Committee
HRA	Health Research Authority
IB	Investigators Brochure
ICF	Informed Consent Form
ICH	International Conference on Harmonisation
IMP	Investigational Medicinal Product
IRB	Independent Review Board
MHRA	Medicines and Healthcare products Regulatory Agency
NHS	National Health Service
RES	Research Ethics Service
OXTREC	Oxford Tropical Research Ethics Committee
PI	Principal Investigator
PIL	Participant/ Patient Information Leaflet
R&D	NHS Trust R&D Department
REC	Research Ethics Committee
RSI	Reference Safety Information
SAE	Serious Adverse Event
SAR	Serious Adverse Reaction

© Copyright: The University of Oxford and Oxford University Hospitals NHS Foundation Trust 2019

SDV	Source Data Verification
SMPC	Summary of Medicinal Product Characteristics
SOP	Standard Operating Procedure
SUSAR	Suspected Unexpected Serious Adverse Reactions
TMF	Trial Master File
TSG	Oxford University Hospitals NHS Foundation Trust / University of Oxford Trials Safety Group

Medical Abbreviations

ACE	Angiotensin Converting Enzyme
ACR	Albumin Creatinine Ratio
ARA	Aldosterone Receptor Antagonist
ARB	Angiotensin II Receptor Blockers
NP	Natriuretic Peptides
BP	Blood Pressure
CKD	Chronic kidney disease
CV	Cardiovascular
CVD	Cardiovascular disease
DM	Diabetes Mellitus
EF	Ejection Fraction
eGFR	Estimated Glomerular Filtration Rate
ESRF	End stage Renal Failure
HbA1c	Glycated Haemoglobin
HRQL	Health Related Quality of Life
ICECAP-A	ICEpop CAPability measure for Adults
KDQOL-SF	Kidney Disease Quality of Life – Short Form Questionnaire
LDL	Low-density Lipoprotein
LV	Left ventricular
LVH	Left ventricular hypertrophy
LVSD	Left Ventricular Systolic Dysfunction
MDRD	Modification of Diet in Renal Disease
NHS	National Health Service
NICE	National Institute of Clinical Excellence
NSAID	Non-steroidal Anti-inflammatory Drug
ONS	Office for National Statistics

© Copyright: The University of Oxford and Oxford University Hospitals NHS Foundation Trust 2019

PWV	Pulse Wave Velocity
QALY	Quality Adjusted Life Year
QoL	Quality of Life
RAAS	Renin-angiotensin-aldosterone System
TIA	Transient Ischaemic Attack
VAS	Visual Analogue Scale

© Copyright: The University of Oxford and Oxford University Hospitals NHS Foundation Trust 2019

5. BACKGROUND AND RATIONALE

Chronic kidney disease (CKD) is increasingly common, affecting around 10% of the entire population, associated with an age-related decline in renal function that is accelerated in hypertension, diabetes mellitus, obesity and primary renal disorders. While this high (and rising) prevalence is in part due to the ageing population, it is also a result of increases in hypertension and diabetes mellitus as well as a variety of primary renal disorders. CKD is defined and categorised in to 5 stages using glomerular filtration rate (GFR) as well as evidence of renal damage (imaging or proteinuria) in the early stages. The largest group, with over 50% of cases, is CKD stage 3, defined as a GFR of 30 to 59 ml/min/1.73m^2. Population studies have used the four variable Modification of Diet in Renal Disease (MDRD) formula to determine estimated GFR (eGFR) [1]. In patients aged 65 or over, up to 35% have an eGFR of less than 60 mls/min/1.73m^2 [2]. CKD prevalence appears to be increasing, rising from 10% to 13% over the last decade in one large cohort in the United States [3].

CKD and risk of cardiovascular disease

CKD is a major cause of increased mortality and morbidity through increased vascular events and progression to end stage renal failure (ESRF) [4]. These increased events result in CKD having high cost to healthcare systems, with the dialysis required in ESRF benchmarked as at the maximum acceptable cost effectiveness threshold for an intervention by most healthcare systems. However, the most important component of CKD in terms of mortality and morbidity is cardiovascular disease (CVD) [5]. There is a graded inverse relationship between cardiovascular risk and eGFR, independent of age, sex and other riskfactors [6-9] or for creatinine [10]. While the cardiovascular risk of end-stage CKD is extreme, in public health terms the burden resides in early stage (CKD stages 1-3) disease, which is more prevalent, affectingaround 40% of those over 70 years. When added to conventional risk factors, renal markers substantially improve risk stratification and CKD is therefore an important and under-recognised risk factor for CVD in the general population [11].

Although the risks of myocardial infarction and other manifestations of coronary artery disease are increased in CKD, the pattern of CVD is atypical, with a much greater incidence of heart failure and sudden cardiac death than in the general CVD population [12-14]. The main pathological features in CKD that appear to determine this particular cardiovascular risk phenotype are:

a. Left ventricular hypertrophy and fibrosis accompanied by both systolic and diastolic dysfunction: there is a very high prevalence of left ventricular hypertrophy (LVH) in CKD, often accompanied by magnetic resonance imaging evidence of fibrosis, with LVH in over 30% of patients in stage 2 CKD (eGFR 20-29) and Stage 3 CKD (eGFR 30-59) and in 80% of patients at the start of renal replacement therapy [15-17]. Importantly, the increase in LV mass is a strong independent predictor ofmortality in CKD (as in non-CKD states) and regression of LVH is associated with improved cardiacoutcome.

b. Arterial wall thickening, stiffening and calcification (atherosclerosis). Large conduit arteries buffer the changes in pressure resulting from intermittent ventricular ejection. Stiffening of the arteries (loss of arterial compliance) leads to increased systolic and pulse pressure, and the resultant increase in afterload is a major cause of LVH and its progression over time [18-20]. Prospective studies have demonstrated that measures of aortic stiffness, such as aortic pulse wave velocity (PWV), and augmentation of central aortic pressure by early wave reflections (AIx), are strong independent predictors of all-cause and cardiovascular mortality in patients on dialysis [21, 22] and lowering aortic PWV, mainly by use of an ACE-inhibitor, is associated with an improved survivalin

© Copyright: The University of Oxford and Oxford University Hospitals NHS Foundation Trust 2019

dialysis patients [23]. In the latter study, the reduction in aortic PWV was associated with a parallel reduction in mean arterial and pulse pressure in survivors. In contrast, in those dying from cardiovascular events neither pulse pressure nor aortic PWV were significantly modified by ACE inhibition, although mean arterial pressure (the usual measure in clinical practice) was lowered to the same extent as in survivors. All these data suggest that arterial stiffness is not merely a marker of arterial damage but a potentially reversible factor contributing to mortality.

Therefore, although patients with CKD also suffer typical patterns of cardiovascular disease (coronary and peripheral artery atherosclerosis), the excess rates of cardiovascular events in CKD appear to relate more to vascular wall and ventricular changes then to atherosclerosis. The causes of atherosclerosis and LVH in CKD are complex but it is likely that as renal function declines, the onset of sodium overload combined with hypertension, chronic anaemia, oxidative stress and activation of the renin-angiotensin-aldosterone system (RAAS) and sympathetic nervous system all contribute to this development of atherosclerosis, myocardial hypertrophy and fibrosis. Furthermore, many of these factors cause vascular endothelial dysfunction which as well as leading to atherosclerotic disease is a major functional component of arterial stiffening [16]. It is the early development of arterial stiffening, causing loss of arterial compliance, increased afterload and exposure of end organs to high phasic pressures, which is thought to be a key factor in the causation of left ventricular hypertrophy and small vessel damage in the brain and kidney [15].

Given this particular vascular pathophysiology, it is unsurprising that conventional cardiovascular risk factors are less predictive of outcomes in CKD than in the general population, [24] and much less predictive than eGFR and protein excretion [6, 9, 25], even after controlling for variables such as blood pressure [17]. Furthermore, interventions to reduce the increased cardiovascular risk in CKD have proved disappointing, with only limited evidence for traditional therapies in terms of cardiovascular outcomes. For example, the SHARP (Study of Heart and Renal Protection) trial [26] aimed to assess the safety and efficacy of reducing LDL cholesterol in more than 9000 patients with chronic kidney disease with a low dose of a statin (simvastatin 20 mg daily). The trial showed that lowering of LDL cholesterol safely reduced the risk of major atherosclerotic events in patients with CKD. However, the reduction in non-fatal myocardial infarction or coronary death was not significant. There are also limited therapeutic options for the prevention of further renal functional decline. Presently, the only interventions shown to reduce or prevent renal function decline for most patients with CKD is avoidance of renal damage (e.g. treating infections and avoiding NSAIDs in at-risk people), and effective treatment of risk factors, namely hypertension and diabetes mellitus (DM). In addition, drugs acting on the RAAS system offer modest additional benefits to blood pressure (BP) lowering alone in patients with diabetic nephropathy with proteinuria [27].

Better treatment options to provide protection from vascular events or delay progression of CKD are therefore urgently needed, especially given the increasing burden of the disease. Desirable clinical outcomes for any new therapies would be effective and safe reduction of cardiovascular events and premature death and/or delay in progression of renal decline. The most important target CKD population for such preventive interventions are those with CKD stage 3b (eGFR 30-44 ml/min/1.73m^2), since this has high prevalence at 3%, represents progressive renal disease, and is associated with a 12-fold increase in cardiovascular disease (CVD), compared to those with eGFR above 60. In contrast, relative cardiovascular risk is 2 fold in CKD 3a (eGFR 45-59), though the prevalence is nearer 15% [6]. Important new candidates for potential cardio-protection in CKD are drugs that act on the aldosterone pathway of the RAA system.

© Copyright: The University of Oxford and Oxford University Hospitals NHS Foundation Trust 2019

Role of aldosterone in cardiovascular disease

Blockade of RAAS with Angiotensin Converting Enzyme (ACE) inhibitors and Angiotensin II Receptor Blockers (ARBs) has shown mortality benefit in patients with chronic heart failure and in those with, or at high risk of, coronary artery disease [27-29]. The benefits are attributed to prevention of the multiple adverse effects of angiotensin II.

Aldosterone may also be an important mediator of cardiac and vascular damage in many disease states. Mineralocorticoid receptors are present in many tissues, including the brain, heart and blood vessels as well as the kidney and there is aldosterone production within these tissues [30]. These receptors may also be activated by circulating glucocorticoids in the presence of oxidative stress [31]. Local mineralocorticoid receptor activation by aldosterone leads to numerous pathological effects on the cardiovascular system including endothelial injury, inflammation, oxidative stress and fibrosis in the heart and vasculature, as well as the development of hypertension and autonomic dysfunction [30, 32, 33].

Rationale for ARA intervention to reduce cardiovascular events: In animal models, chronic aldosterone infusion and sodium loading results in myocardial fibrosis and ventricular hypertrophy in rats, while treatment with ARAs prevents aortic and myocardial fibrosis even in the absence of blood pressure lowering [33, 34]. In aldosterone treated stroke-prone hypertensive rats, spironolactone exerts a strong protective effect against the development of nephrosclerotic and cerebrovascular lesions [35]. In humans, studies have shown that primary aldosteronism is associated with a greater LV mass and higher risk of adverse cardiovascular events than control hypertensive populations and in patients after myocardial infarction, plasma aldosterone concentration within the normal range predicts an adverse prognosis [36-38]. A recent study of subjects undergoing coronary angiography confirmed an independent association of plasma aldosterone levels with total and cardiovascular mortality [39].

Importantly, there are reliable and large studies that show that targeting aldosterone improves outcomes in established cardiovascular disease. In heart failure, a human disease state that like CKD is characterised by sodium overload and high levels of aldosterone production, the addition of the ARA spironolactone (RALES) in severe heart failure [40], or eplerenone (EPHESUS) [41] in post infarction heart failure and in mild to moderate chronic heart failure (EMPHASIS) [42], to standard therapy including ACE inhibition, reduced mortality by 30%, which therefore has a greater impact on mortality than both ACE inhibitors and beta-blockers. Further, treatment with ARAs in addition to ACE inhibitors prevents adverse LV remodelling after myocardial infarction and effectively reduces LVH in drug resistant hypertension [43]. The mechanisms of action of aldosterone include up-regulation of AT1 receptors and direct effects on fibroblast collagen synthesis as well as decreased matrix metallo-proteinase secretion [30]. An anti-fibrotic effect of ARA therapy may also be important. After myocardial infarction circulating markers of collagen turnover and fibrosis were reduced by ARA therapy [43] and in the RALES study myocardial collagen turnover was significantly reduced by spironolactone, and the fall in the marker of this index was related to the mortality benefit [44].

Role of aldosterone and potential for ARA in progression of renal disease

Angiotensin Converting Enzyme inhibitors *(*ACE) inhibitors and Angiotensin II Receptor Blockers (ARBs) appear superior to other blood pressure (BP) lowering drugs in slowing the progression of CKD, though the effect may be marginal [27]. These agents are therefore widely recommended in international guidelines as 'Reno-protection' for CKD patients, especially those with proteinuria or diabetes mellitus.

Renal specialists have avoided use of ARA drugs because of perceived risk of azotaemia and hyperkalaemia,

© Copyright: The University of Oxford and Oxford University Hospitals NHS Foundation Trust 2019

though similar restrictions were applied to ACE inhibitors until outcome data were reported. There are, however, accumulating data on combined treatment with ACE and ARAs to improve renal function in patients with CKD [45]. Animal experiments have shown that aldosterone can mediate renal injury and that ARAs, such as eplerenone, effectively reduce this [46-48]. Importantly, ARAs are similarly effective in low aldosterone models of CKD probably reflecting the importance of local (paracrine) aldosterone synthesis. [49] In humans, small studies have reported that adding ARAs to ACE inhibitors or ARBs reduces proteinuria and may slow progression of renal disease [50, 51]. From a safety perspective, even oligoanuric haemodialysis patients can tolerate spironolactone in low doses [52].

Diagnosis of CKD

The current UK standard estimating equation for GFR, Modification of Diet in Renal Disease study (MDRD) [53], results in an underestimation bias for higher levels of renal function. The more recent Chronic Kidney Disease Epidemiology Collaboration (CKD-EPI) equation [54] has been validated in general populations (excluding very elderly persons) [55, 56], as well as in different ethnic groups with appropriate equation modification (as per MDRD [57, 58]) and has shown greater accuracy. The MDRD equation has some utility in cardiac risk prediction [59, 60] but CKD-EPI based CKD staging improves risk prediction [61, 62] and this may influence policy in the United States with plans to switch to CKD-EPI for GFR reporting [63]. Evidence for the optimal GFR estimation method in primary care populations has not been systematically summarised. BARACK D will measure CKD using both criteria and will provide important new comparator data to inform this debate.

5.1. Rationale for Current Trial

CKD is common and increasing in prevalence. Cardiovascular disease is a major cause of morbidity and death in CKD, though of a different phenotype to the general CVD population. Currently, few therapies have proved effective in modifying the increased CVD risk or the rate of renal decline in CKD. There are accumulating data that aldosterone receptor blockers (ARAs) may offer cardio-protection and delay renal impairment in patients with the CV phenotype in CKD. The use of ARA in CKD has therefore been increasingly advocated and even termed the 'renal aspirin' [64]. To date, however, no large study of ARAs with renal or CVD outcomes is underway.

There are recent data that indicate beneficial effects of ARA therapy on surrogate markers for cardiovascular disease risk in patients with CKD, i.e. not just in those with established advanced cardiovascular disease, such as heart failure. This is important because there are presently limited therapeutic options to reduce overall cardiovascular risk in CKD, with modest effects of LDL reduction shown in the recent SHARP study [26] and sub-studies of large ACE inhibitor and statin trials only suggesting limited cardiovascular benefits in patients with early stage CKD [11, 28].

The Birmingham CRIB-2 study, in which two of the co-applicants to BARACK D were involved (Ferro & Townend), recently showed that spironolactone provided significant beneficial effects on validated intermediate cardiovascular end points of prognostic value, including LV mass and arterial stiffness [65]. In a placebo controlled double blind trial 112 patients with stage 2 and 3 CKD with good blood pressure control on established treatment with ACE inhibitors or ARBs were treated in an active run-in phase with spironolactone 25 mg once daily and then randomised to continue spironolactone or to receive a matching placebo. LV mass (cardiac magnetic resonance) and arterial stiffness (augmentation index, and aortic distensibility using MR imaging) were measured before run in and after 40 weeks of treatment. Compared

© Copyright: The University of Oxford and Oxford University Hospitals NHS Foundation Trust 2019

with placebo, the use of spironolactone resulted in highly significant reductions in LV mass and arterial stiffness (pulse wave velocity, augmentation index and aortic distensibility), improved myocardial diastolic function and collagen turnover [65]. These clinical findings were attributed to a reduction in arterial and myocardial inflammation and fibrosis but may also be a function of the considerable human and animal evidence base that aldosterone receptor antagonism improves endothelial dependent vasodilatation and vascular nitric oxide bioactivity [66]. Further recent data have shown that ARA therapy in early CKD prevented progression of carotid intima-media thickness in haemodialysis patients [67]. These recent clinical data on the effect of ARA on intermediate vascular outcomes have resulted in calls for definitive trials [68, 69]. In a recent review, the RALES Chief Investigator Bertram Pitt was cautiously optimistic that use of an ARA '…will reduce the mortality and morbidity associated with CKD, as well as prevent its progression to end-stage renal disease with all of its health-care and health-cost consequences" [68].

ARA therapy might therefore be an effective candidate for improved cardiovascular outcomes, through the prevention of aldosterone mediated vascular endothelial dysfunction as well as widespread cardiovascular inflammation, fibrosis, hypertrophy. Since spironolactone is well recognised as an effective anti-hypertensive agent for patients with hypertension, even when this is resistant to other drugs [70], the intensive phenotyping of blood pressure, LV function and arterial stiffness in BARACK D will enable modelling of the extent to which any positive results may be explained by any blood pressure differences between study arms. The 25mg dose of spironolactone used in BARACK D, and most clinical trials in which it has been involved, is similar to that used in hypertension and heart failure cases which are states characterised by excess cardiovascular risk and with a high probability of co-morbid CKD.

© Copyright: The University of Oxford and Oxford University Hospitals NHS Foundation Trust 2019

6. OBJECTIVES AND OUTCOME MEASURES

	Objectives	Outcome Measures	Timepoint(s)
Primary	To determine the effect of aldosterone receptor antagonism on mortality and cardiovascular outcomes (onset or progression of cardiovascular disease) in patients with stage 3b CKD.	Time from randomisation until the first occurring of:<br>• Death or<br>• Hospitalisation for heart disease (coronary heart disease, arrhythmia, atrial fibrillation, sudden death, resuscitated sudden death), stroke, transient ischaemic attack, peripheral arterial disease or heart failure or<br>• First onset of any condition listed above not present at baseline.<br>Primary endpoints will be adjudicated by an independent endpoints committee blinded to treatment arm.	Time from randomisation to first occurrence
Primary Long term	To determine the effect of aldosterone receptor antagonism (even short-term use) on long term mortality and cardiovascular outcome in patients with stage 3b CKD.	Annual rates of:<br>• Death,<br>• Hospitalisation for heart disease (coronary heart disease, arrhythmia, atrial fibrillation, sudden death, resuscitated sudden death), stroke, transient ischaemic attack, peripheral arterial disease or heart failure.<br>• First onset of any condition listed above not present at baseline.	Annual rates, collected via medical notes review

© Copyright: The University of Oxford and Oxford University Hospitals NHS Foundation Trust 2019

Secondary	To determine the effect of adding an aldosterone receptor antagonism in patients on 1-5 below:<br><br>1. The individual components of the composite primary outcome	• Hospitalisation or new onset heart disease (coronary heart disease, arrhythmia, atrial fibrillation, suddendeath, resuscitated sudden death), stroke, transient ischaemic attack, peripheral arterial disease or heart failure.	• Total occurrences
	2. Measures of cardiovascular haemodynamics	• Change in blood pressure annually and at final visit.	• Annually and at final visit.
	3. Measures of renal function	• Changes in NP<br>• Change in ACR<br>• Changes in eGFR	• Change from baseline, annually and to final visit for NP, ACR and eGFR
	4. Healthcare cost evaluation	• Change in health status on EQ-5D-5L, KDQol, (ICECAP-A and Qol VAS – Oxford only) and NHS resource use (records).	• Change from baseline, annually and to final visit
	5. Safety	• Rates of hypotension (<100mmHg systolic or >20 mmHg systolic drop on standing)<br>• Rates of adverse events<br>• Rates of hyperkalaemia	• Total occurrences<br><br>• Total occurrences

© Copyright: The University of Oxford and Oxford University Hospitals NHS Foundation Trust 2019

7. TRIAL DESIGN

7.1. Summary of Trial Design

- A PROBE trial: Eligible patients, from a minimum of 120 practices recruited by 6 NIHR School for Primary Care Research departments and collaborating renal specialist groups, with previously recorded blood test results suggesting CKD stage 3b will be invited to take part in the study and randomised between the ARA spironolactone 25mg OD on top of routine care versus routine care. Blood pressure in both groups will be titrated (monitored and adjusted accordingly) by the physicians against NICE guideline standards and routine checks of electrolytes undertaken. Primary outcome will be time to changes in cardiovascular events (coronary heart disease, arrhythmia, atrial fibrillation, suddendeath, resuscitated sudden death), stroke, transient ischaemic attack, peripheral arterial disease or heart failure), either new onset of or hospitalisation for cardiovascular disease, or death (regardless of cause).

An internal pilot will be conducted which, in addition to testing study procedures and documentation, will test our assumptions regarding:

i) practice uptake of the invitation to participate

ii) rates of eligible CKD patients in practice populations on existing disease registers

iii) the response rates to patient invitations

iv) the rates of consent at baseline visits.

These early recruitment data will be used after 4 months to determine whether any changes are needed to overall recruitment strategy in the other centres e.g. whether numbers of practice sites need to be supplemented.

7.2. Primary and Secondary Endpoints/Outcome Measures

As listed in section 6.

8. PARTICIPANT IDENTIFICATION

8.1. Trial Participants

Overall Description of Trial Participants

Patients identified by their GPs or physicians who have been diagnosed with CKD stage 3b (eGFR 30-44 ml/min/1.73m^2 but widened to 30-50 ml/min/1.73m^2 following initial recruitment to encompass larger than anticipated measurement error/fluctuations) based on their recent blood tests. Patients declining to participate will be asked for consent to review their records for comparative data.

Inclusion Criteria

Participants must fulfil either the **Search 1** or **Search 2** criteria specified below:

Search 1

- Evidence of stage 3b CKD using the MDRD equation. This includes patients on the CKD register undergoing annual monitoring who have had two or more recent blood samples in the 30-50

© Copyright: The University of Oxford and Oxford University Hospitals NHS Foundation Trust 2019

ml/min/1.73m^2 range in the preceding 24 months, with a minimum of 6 weeks between tests

- Where only one test has been performed in the preceding 24 months and is in the 3b range, the patient will be invited to attend the baseline visit at least 6 weeks from the initial test, the eGFR result from this can be taken as the second confirmatory test. Physicians will also be reminded that standard care suggests a second confirmatory test.

Search 2

- Patients with eGFR results in the preceding 24 months with a reading of 25-29 ml/min/1.73m^2
- Participant is willing and able to give informed consent for participation in the study.
- Male or Female, aged 18 years or above.
- Able (in the recruiting physician's opinion) and willing to comply with all study requirements.
- Willing to allow his or her General Practitioner and consultant, if appropriate, to be notified of participation in the study.
- Willing to provide contact details to the Research Team (encompassing recruitment centre and practice staff), for use at any time should the need arise, on trial related matters.
- If the participant is a female of child-bearing potential, they are willing to ensure effective contraception during the trial period.

8.2. Exclusion Criteria

The participant may not enter the study if ANY of the following apply:

- Female participants who are pregnant, lactating or planning pregnancy during the course of the study.
- Type 1 diabetes mellitus
- Terminal disease or felt otherwise unsuitable by their physician
- Chronic heart failure clinical diagnosis or known LVSD with EF<40%
  Recent myocardial infarction (within 6 months)
- Active cancer with less than 1 year life expectancy or in palliative care.
- Alcohol or drug abuse.
  - Suspected or known current hazardous or harmful drinking, as defined by an alcohol intake of greater than 42 units every week.
  - Suspected or known current substance misuse.
- Most recent potassium result >5.5 mmol/L, where not thought to be spurious, or previous raised potassium needing a reduced dose of ACEI/ARB or intolerance to spironolactone.
- eGFR >60 ml/min/1.73m^2 in the last 6 months and no identifiable reason for a temporary reduction in eGFR.
- Serum potassium at baseline over 5 mmol/L.
- Documented Addisonian crisis and/or on fludrocortisone.
- Documented symptomatic hypotension or baseline systolic blood pressure under 100mmHg.
- Recent acute kidney injury or admission for renal failure.
- ACR > 70 mg/mmol.
- Prescription of medications with known harmful interactions with spironolactone as documented in the British National Formulary including tacrolimus, lithium and cyclosporine.
- Any other significant disease or disorder which, in the opinion of the recruiting physician, may either put the participants at risk because of participation in the study, or may influence the result of the

© Copyright: The University of Oxford and Oxford University Hospitals NHS Foundation Trust 2019

study, or the participant's ability to participate in the study.

8.3. Expenses and Benefits

All Participants will be reimbursed receipted, reasonable travel expenses.

9. TRIAL PROCEDURES

See Appendix B for details of study visits and procedures.

9.1. Recruitment, Screening and Eligibility Assessment

Potential subjects will be identified by searching routine electronic clinical records (eCRs) for patients with biochemical evidence of CKD stage 3b (eGFR 30-44 ml/min/1.73m^2 but widened to 30-50 ml/min/1.73m^2 following initial recruitment to encompass larger than anticipated measurement error/fluctuations) identified from one blood test in the last two years. The GP practice/renal specialist group will then send out an invitation letter inviting the patients to attend a baseline assessment and eligibility visit. A reply slip, pre-paid envelope and alternative contact details (e.g. e-mail address and phone number) will be provided for expressions of interest. Further to this through the invitation letter patients will be informed of an intention to carry out phone calls within two weeks of the initial mail out. In order to prevent patients receiving unwanted phone calls they will be given opportunity to opt out of receiving this call via a phone number, postal reply slip in a prepaid envelope, or email. These calls will have dual purpose, being facilitated by a member of the research team; firstly to identify any concerns, anxieties, or questions the patients may have, as patients may not have been told they have "Chronic Kidney Disease" in consultation previously, and may have been given an alternative description for example "renal impairment", by their physician. Secondly this will act as reminder call to patients giving them the opportunity to seek any further information they require with regard to the study and will allow them to express interest in the study if they so wish. Approximately two weeks after the initial mail out a reminder letter will be sent to non-responders (including patients who have not been contactable by phone), along with, in a subgroup, a feedback form for negative responders to complete should they wish.

A further search will be performed, again for the preceding 24 months, to identify patients with one reading in the eGFR range 25-29 ml/min/1.73m^2 (with no eGFR >60 ml/min/1.73m^2 in the last 6 months). Patients identified from these searches will be invited to a screening visit, using the same strategy as described above and, if found to be within the 30-50 ml/min/1.73m^2 range, asked to attend the baseline visit a minimum of 6 weeks later. If the patient's eGFR at this visit is not in the 30-50 ml/min/1.73m^{2}range but still within the wider range (25-29 ml/min/1.73m^2) the patient will be asked to return 3 months later for a repeat screening visit. This process will repeat throughout the recruitment period for as long as the patient is willing. The initial screening visit will consist of an informed consent procedure and a single renal profile blood test to encompass potassium levels and eGFR.

Initial calculations showed that, for the average practice, 180 patients are likely to meet Stage 3b CKD criteria. Assuming that around 80% of these patients are eligible and at least 50% of these are willing to take part (based on our experience recruiting to heart failure studies which have a similar age distribution as patients with CKD), then 72 patients may be recruited per practice, requiring 37 practices in total, but increased to 60 to allow for poor recruiting practices, or 15 practices per Townsend quartile of deprivation. To improve the representativeness of the trial population, the number of practices per recruiting centre will be increased to 20 with the intention of reducing these numbers by 50% and giving 30 practices per Townsend quartile of deprivation.

© Copyright: The University of Oxford and Oxford University Hospitals NHS Foundation Trust 2019

Following analysis of the initial patient recruitment data, 20 practices having mailed-out, the number of practices recruited by the 6 main NSPCR hubs was increased to a minimum of 300, to be initiated in a phased manner.

9.2. Informed Consent

Informed consent will be taken according to the PC-CTU Standard Operating Procedure (PC-CTU_SOP_TM107) “Obtaining Informed Consent”. A Patient Information Leaflet will be given by the Research Team to the patient following identification as a potential participant. This leaflet describes the purpose of the study, explains in detail what is required of participants, discusses potential risks and benefits, and provides contact details for the Research Team. The patient will be given adequate time to consider participation and read the leaflet, consulting with family or friends or any other independent advisors if needed, before seeing the Research Team for the first study consultation. At the baseline assessment informed consent will be taken, by a suitably qualified member of the Research Team, who will have received training in Good Clinical Practice and will be authorised to take consent by the Chief Investigator, delegated through the Principal Investigators where applicable. The Consent Form will be signed and dated both by the patient and the member of the Research Team taking consent. No study related procedures will take place prior to the signing of the Consent Form. It is clearly stated that the participant is free to withdraw from the study at any time for any reason without prejudice to future care, and with no obligation to give a reason for withdrawal. If the patient requires more time to make a decision on participation, then a further consultation will be arranged. Participants will be asked to consent to being contacted by the Research Team in the event they fail to return for any of the trial follow-up. Consented participants will be asked to complete a Contact Details Form which includes all of their relevant contact details and indication as to their preferred method of contact by the Research Team. Consent will be taken to allow relevant sections of patient medical notes and data collected during the study to be looked at by responsible individuals from the University of Oxford and collaborating partners, regulatory authorities (including the MHRA) and the NHS trust, where it is relevant to taking part in the trial. A copy of the signed Consent Form will be given to the participant and a further copy will be sent with the Contact Details Form to the Research Team. One copy of the consent will remain in the patient’s records at the GP practice/specialist renal group.

Patients declining to participate will be asked if they are willing to provide separate written consent to review their records for comparative data. Data will be manually recorded in a separate CRF and transferred to the trial database.

9.3. Randomisation

Randomisation will be carried out using Sortition, a validated randomisation system within the PC-CTU. We will stratify by Practice ensuring a balance of the two arms within each practice. Patients will be randomised to treatment with spironolactone 25 mg once daily prescribed on top of routine care or to continue with routine care alone.

9.4. Blinding and code-breaking

BARACK D is a PROBE trial where neither the patients nor physicians are blinded to the trial treatment, but the primary endpoints will be assessed by an independent endpoint committee who are blinded to the treatment arm.

© Copyright: The University of Oxford and Oxford University Hospitals NHS Foundation Trust 2019

9.5. Baseline Assessments

Potentially eligible patients will be invited to attend a baseline clinic at a trial practice where the trial will be explained. Informed consent will be obtained and baseline assessments performed.

A subset of patients will form the intensively phenotyped group who will undergo additional trial procedures as described below and in the procedure schedule (Appendix B). The intensive phenotyping of 24hr blood pressure and arterial stiffness in BARACK D will enable modelling of the extent to which any positive results may be explained by any blood pressure differences between study arms.

<u>Following consent</u>, all patients will have the following information taken and investigations performed at the initial baseline visit:

- Age
- Gender
- Self-assigned ethnicity
- Residential postcode
- Clinical history
- Past medical history
- Current medication
- Smoking status
- Physical examination
- Weight
- Height
- Waist circumference (using validated method)
- Office BP measurement using a British Hypertension Society validated automated device after 5 minutes rest
- Venepuncture for routine haematology and biochemistry including renal function (including eGFR calculated using MDRD and CKD-EPI formulae, hepatic and bone profiles, full blood count, fasting blood sugar, HbA1c, lipids, and, where local labs allow, BNP). Tests will be performed by a suitably qualified member of the Research Team (e.g. G.P. or research/practice nurse). Where appropriate to the Department of Health guidelines (e.g. routine tests) funding will be provided by the CLRN. Any outstanding costs will be met by the funder. Where transport and local coordination allows (initially involving specific practices within the Oxford recruitment area) an additional blood sample will be taken and stored for future genetic and protein testing.
- Urinalysis using albumin:creatinine ratio (ACR). Where transport and local coordination allows (initially involving specific practices within the Oxford recruitment area) an additional urine sample will be taken and stored for future testing.
- 12 lead electrocardiograph – where practice equipment availability allows
- Quality of life questionnaires (EQ-5D-5L and KDQOL-SF questionnaires, (ICECAP-A and Qol VAS – Oxford only))
- Issue diary card to monitor side effects of trial medication
- Pregnancy tests will be performed on women of childbearing potential, if deemed necessary, at the discretion of the physician

© Copyright: The University of Oxford and Oxford University Hospitals NHS Foundation Trust 2019

Following the baseline visit, as with all laboratory analyses returned to the GP practice/specialist renal group under routine care as the same mechanisms will be utilised, blood results (which are normally returned within 1 working day) will be reviewed as soon as practically possible and no later than 72hrs after receipt, and the reports signed by the recruiting physician, or delegate (for example the patient's own GP) who will record the results in the CRF including information on whether they are normal, abnormal but not clinically significant, or abnormal AND clinically significant. In the latter case the eligibility of the participants will be reviewed. The patients' General Practitioner (GP) will be referred to, in order to confirm eligibility, if:

- BP ≥ 180/110mmHg
- ACR ≥ 70 mg/mmol: to refer to GP to consider referral to nephrology specialist if patients have not been reviewed by nephrologist in the past 5 years since the diagnosis.
- ACR= 30-69 mg/mmol and BP ≥ 140/90 mmHg and NOT on either angiotensin converting enzyme inhibitor (ACEI) or angiotensin receptor blocker (ARB): to refer to GP to consider for ACEI/ARB. Patients will be re-invited to participate in BARACK-D study after they have been on ACEI/ARB for at least 6 weeks.
- ACR= 30-69 mg/mmol with haematuria: to refer to GP for review.

Once eligibility is confirmed, the physician will randomise the patient (by accessing Sortition to obtain the randomisation code), produce the necessary prescription (in some instances through the patient's own GP, depending on standard practice mechanisms), if applicable, and issue to the patient where necessary. The physician will also book an appointment for the patient to return for their next visit after taking spironolactone for 7 days where assigned to the treatment arm of the trial, or 7 days following randomisation where assigned to the routine care arm.

9.6. Subsequent Visits

Subsequent assessment will continue for both treatment arms for a further 36 months with follow up visits at weeks 1, 2, 4, 12, 26, and then every 13 weeks to 156 weeks. Windows either side of the visits will be two days for V1 and V2, 4 days at V3 and V4, 7 days for V5 and two weeks thereafter (all calculated from date of randomisation). Measurements at each follow-up visit will vary according to the schedule in Appendix B but will consist of a combination of:

- Office BP measurement, using a validated automated device;
- Venepuncture for creatinine & electrolyte levels;
- eGFR (MDRD and CKD-EPI estimations);
- Monitoring for side effects.
- Additional bloods for fasting blood sugar and HbA1c, BNP (where local labs allow), lipids, full blood count and samples for future analysis;
- QoL questionnaires;
- Issue of drug monitoring diary card.
- Urinalysis using albumin: creatinine ratio.
- Home blood pressure measurement recorded on diary card.

Patients will also be supplied with a validated home blood pressure monitoring machine, along with an additional diary card and an instruction sheet, for 1 week every 6 months to document their self-assessed blood pressures. They will take 2 readings twice daily, i.e. 2 each morning and 2 each evening over the

© Copyright: The University of Oxford and Oxford University Hospitals NHS Foundation Trust 2019

week. The readings for the first two days will be discarded and the mean of the remaining readings taken as the home blood pressure level.

Physicians will be strongly encouraged to manage blood pressure according to NICE CKD guidelines (2008): Blood Pressure Targets: CKD and ACR <70 mg/mmol: systolic blood pressure target of <140 mmHg (target range 120–139 mmHg) and diastolic blood pressure target <90 mmHg. Choice of antihypertensive agents: ACE inhibitors/ARBs if not already prescribed will be offered to people with hypertension and ACR ≥30 mg/mmol. We estimate that around two thirds of patients in BARACK D will be additionally taking an ACE inhibitor or ARB. The remainder, (people with CKD and hypertension and ACR <30 mg/mmol) will be offered a choice of antihypertensive treatment according to the NICE guidance on hypertension (NICE clinical guideline CG127 or its update) to prevent or ameliorate progression of CKD.

Patients will also be flagged for long term follow up of study endpoints, by digital data capture,continuing until 2049. Separate consent will be taken for this extended follow up; a patient information leaflet and consent form will be sent to the patient's home and if consented, the patient will keep a signed copy of the consent form and return two copies to the research team.

9.7. Sample Handling

Samples taken during the study are routine tests and will therefore be taken and analysed using routine processes in the GP practices.

9.8. Early Discontinuation/Withdrawal of Participants

Each participant has the right to withdraw from the study at any time in line with the following criteria:

1. Withdrawal from treatment (follow-up continued)
2. Complete withdrawal from trial excluding notes review (without participant involvement)
3. Complete withdrawal

In addition, the recruiting physician may discontinue a participant from the study treatment at any time if it is considered necessary for any reason including the following general rules:

- Ineligibility (either arising during the study or retrospective having been overlooked at screening)
- Significant protocol deviation as judged by the trial physician
- Significant non-compliance with treatment regimen or study requirements
- An adverse event which requires discontinuation of the study medication or results in inability to continue to comply with study procedures
- Disease progression which requires discontinuation of the study medication or results in inability to continue to comply with study procedures
- Lost to follow up

In all cases, where possible, follow-up and inclusion in the intention-to-treat analysis, will continue. Safety monitoring will include the following discontinuation rules:

Hyperkalaemia: In RALES, incidence of serious hyperkalaemia was 2% although patients with a creatinine of > 221 were excluded [17]. In EPHESUS, eplerenone caused a K^+ > 5.5 mmol/L in 10% of patients with a GFR of < 70 ml/min [30]. In CRIB-2 [22], during the open label run in only 1 patient was withdrawn due to hyperkalemia (K^+>6.5) and 6 had a K^+ of >5.5 mmol/L requiring dose reduction to alternate days. During

© Copyright: The University of Oxford and Oxford University Hospitals NHS Foundation Trust 2019

the double-blind phase only 2 patients on ARA and 2 on placebo had a K^+ of >5.5 mmol/L. For BARACK D, serum K^+ and creatinine will be checked at all visits. Patients will stop trial medication if systemically unwell due to intercurrent infection, diarrhoea or need for surgical intervention for any reason. Study drug will be re-started one week after the recruiting physician is satisfied recovery has taken place; serum K^+ and creatinine will be rechecked at weeks 1 and 2 following resumption. The protocol below will be followed in the event of hyperkalaemia:

- Serum potassium below 5.4 mmol/L, no action;
- Between 5.5-5.9, reduce dose to 25mg alternate days;
- 6.0-6.4 stop study drug and restart after 7 days on alternate days and if remains over 6.0 withdraw patient from trial treatment;
- >6.5 appropriate management and withdraw patient from trial treatment.

Deterioration of renal disease: If there is a deterioration of 20% in eGFR between visits or a deterioration of 25% from baseline to any visit, then withdraw the patient from trial treatment and refer to specialist care;
Hypotension: If there is >20 mmHg systolic postural drop in blood pressure with symptoms during the trial and/or the systolic blood pressure drops to below 100 mmHg then the trial medication will be discontinued;

If withdrawn from the trial, the reason for withdrawal will be recorded on the trial withdrawal form and if due to an adverse event, the Research Team will arrange for follow-up visits or telephone calls until the adverse event has resolved or stabilised.

9.9. Definition of End of Trial

The end of trial will be defined as the date of the last data capture for the last participant in December 2049, allowing 30 years follow-up of all participants. The trial will have an independent TSC and DMEC who will assess the study feasibility as the trial progresses and will have 'stop rule' authority to advise early termination of the trial in the event of safety concerns or futilityeither through poor recruitment, lack of events, or lack of any treatment effect. A formal futility and feasibility analysis will be performed by the DMEC to assess recruitment and retention which will determine whether criteria for the trial to proceed have been met.

10. TRIAL INTERVENTIONS

10.1. Investigational Medicinal Product(s) (IMP) Description

Spironolactone has been selected as the trial ARA, to be used in the "Standard Care + Spironolactone" arm, since it has a large evidence base for effective treatment in hypertension and heart failure. There are considerable data from these trials on the drug's renal safety in high risk cardiovascular populations. Spironolactone is also the most cost effective ARA being available as a generic prescription. The modest cost of the prescriptions to the NHS will be treated as an excess treatment cost but this is not anticipated as likely to cause local barriers to recruitment.

Clinical trial labelling will not be required in accordance with Article 14 of the EU clinical trial directive.

10.1.1. Blinding of IMPs

© Copyright: The University of Oxford and Oxford University Hospitals NHS Foundation Trust 2019

There is no blinding of the trial medication for the participants or their GPs in this study.

10.1.2. Storage of IMP

Spironolactone 25mg will be prescribed on FP10 by the study recruiting physician using the physician's local pharmacies, processes and systems. As such, there will be no trial specific study treatment requirements. The trial treatment regime will be 25mg spironolactone once daily for the duration of the trial.

10.1.3. Compliance with Trial Treatment

Study treatment compliance will be self-monitored throughout the trial using a medication monitoring diary card. For participants assigned to the spironolactone treatment arm, where appropriate, for example if compliance cannot be verified through patient report, prescription uptake will also be verified by the patient's physician through database searches of prescription collection.

10.1.4. Accountability of the Trial Treatment

The study treatment will be prescribed on FP10 by the recruiting physician, or delegate (for example the patient's own GP, depending on standard practice mechanisms) and therefore no drug accountability processes will be necessary.

10.1.5. Concomitant Medication

If participants on the spironolactone arm develop medical conditions which require treatment with medications known to have harmful interactions with spironolactone as listed in the SmPC, then their prescription will be halted [75] but follow-up will continue.

10.1.6. Post-trial Treatment

Throughout the trial the participant remains the responsibility of their GP practice/specialist renal group and therefore under normal care.

10.2. Other Treatments (non-IMPS)

There are no non-IMPs being used in this Study.

10.3. Other Interventions

There are no additional interventions being tested in this study.

11. SAFETY REPORTING

11.1. Adverse Event Definitions

Adverse Event (AE)	Any untoward medical occurrence in a participant to whom a medicinal product (or study intervention) has been administered, including occurrences which are not necessarily caused by or related to that product.

© Copyright: The University of Oxford and Oxford University Hospitals NHS Foundation Trust 2019

Adverse Reaction (AR)	An untoward and unintended response in a participant to an investigational medicinal product which is related to any dose administered to that participant.<br>The phrase "response to an investigational medicinal product" means that a causal relationship between a trial medication and an AE is at least a reasonable possibility, i.e. the relationship cannot be ruled out.<br>All cases judged by either the reporting medically qualified professional or the Sponsor as having a reasonable suspected causal relationship to the trial medication qualify as adverse reactions.
	Causality of all cases must be judged by a medically qualified doctor.
Serious Adverse Event (SAE)	A serious adverse event is any untoward medical occurrence that:<br>• results in death<br>• is life-threatening<br>• requires inpatient hospitalisation or prolongation of existing hospitalisation<br>• results in persistent or significant disability/incapacity<br>• consists of a congenital anomaly or birth defect*.<br>Other 'important medical events' may also be considered a serious adverse event when, based upon appropriate medical judgement, the event may jeopardise the participant and may require medical or surgical intervention to prevent one of the outcomes listed above.<br>NOTE: The term "life-threatening" in the definition of "serious" refers to an event in which the participant was at risk of death at the time of the event; it does not refer to an event which hypothetically might have caused death if it were more severe.<br>*NOTE: Pregnancy is not, in itself an SAE. In the event that a participant or his/her partner becomes pregnant whilst taking part in a clinical trial or during a stage where the fetus could have been exposed to the medicinal product (in the case of the active substance or one of its metabolites having a long half-life) the pregnancy should be followed up by the investigator until delivery for congenital abnormality or birth defect, at which point it would fall within the definition of "serious".
Serious Adverse Reaction (SAR)	An adverse event that is both serious and, in the opinion of the reporting Investigator, believed with reasonable probability to be due to one of the trial treatments, based on the information provided.

© Copyright: The University of Oxford and Oxford University Hospitals NHS Foundation Trust 2019

Suspected Unexpected Serious Adverse Reaction (SUSAR)	A serious adverse reaction, the nature and severity of which is not consistent with the Reference Safety Information for the medicinal product in question set out:<br>• in the case of a product with a marketing authorisation, in the approved summary of product characteristics (SmPC) for that product<br>• in the case of any other investigational medicinal product, in the approved investigator's brochure (IB) relating to the trial in question.

NB: to avoid confusion or misunderstanding of the difference between the terms "serious" and "severe", the following note of clarification is provided: "Severe" is often used to describe intensity of a specific event, which may be of relatively minor medical significance. "Seriousness" is the regulatory definition supplied above.

11.2. Assessment results outside of normal parameters as AEs and SAEs

The following normal parameters will also be recorded as AEs:

- significant decrease in eGFR (20% between visits, or 25% from baseline),
- rise in potassium levels (to above 5.5), and
- drop in BP (to below 100 in both arms

11.3. Assessment of Causality

The relationship of each adverse event to the trial medication must be determined by a medically qualified doctor according to the following definitions:

- **Unrelated** – where an event is not considered to be related to the IMP
- **Possibly** – although a relationship to the IMP cannot be completely ruled out, the nature of the event, the underlying disease, concomitant medication or temporal relationship make other explanations possible.
- **Probably** – the temporal relationship and absence of a more likely explanation suggest the event could be related to the IMP.
- **Definitely** – the known effects of the IMP, its therapeutic class or based on challenge testing suggest that the IMP is the most likely cause.

All AEs (SAEs) labelled possibly, probably or definitely will be considered as related to the IMP.

11.4. Procedures for Reporting Adverse Events

All site staff are appropriately trained in the procedures to follow and the forms to use by the PC-CTU prior to study initiation. Regular central and on-site monitoring will be used to ensure that all adverse events are identified and acted on appropriately.

All adverse events will be recorded at trial visits for the initial 6 months of follow-up by the member of the

© Copyright: The University of Oxford and Oxford University Hospitals NHS Foundation Trust 2019

Research Team conducting that visit for the previous inter-visit period. Following this initial 6 month period, only the following AEs will be monitored by the member of the Research Team performing that visit. Safety concerns (i.e. significant decrease in eGFR (20% between visits, or 25% from baseline), rise in potassium levels (to above 5.5), and drop in BP (to below 100 in both arms))

- Enlargement of breasts in men and women
- Erectile dysfunction
- Irregular periods
- Vaginal bleeding after the menopause
- Deepening of the voice in women, change in the tone of voice in men
- Excessive hair growth
- Tiredness
- Palpitations
- Numbness and tingling

AEs considered related to the study medication as judged by a medically qualified member of the Research Team or the Sponsor will be followed until resolution or the event is considered stable, clinically insignificant or asymptomatic. All related AEs that result in a participant's withdrawal from the study or are present at the end of the study, should be followed up until a satisfactory resolution occurs.

It will be left to the recruiting physician's clinical judgment whether or not an AE is of sufficient severity to require the participant's removal from treatment and, if treatment is withdrawn, the reason will be recorded. A participant may also voluntarily withdraw from treatment due to what he or she perceives as an intolerable AE. If either of these occurs, the participant must undergo an end of study assessment and be given appropriate care under medical supervision until symptoms cease or the condition becomes stable.

- The severity of events will be assessed on the following scale: 1 = mild, 2 = moderate, 3 = severe.

The relationship of AEs to the study medication will be assessed by a medically qualified member of the Research Team.

11.5. Reporting Procedures for Serious Adverse Events

All SAEs occurring during the study, (from randomisation until 30 days following the last dose of trial medication/ last visit, whichever is the latter, either observed by the site physician or reported by the participant, whether or not attributed to study medication, will be recorded on the CRF and forwarded by the site to PC-CTU, using the "PC-CTU SAE Report Form" following assessment for seriousness and relatedness by the site clinician. This form will becompleted and faxed or emailed to the PC-CTU using the details quoted on the report form. As a minimum,the following information will be recorded:

- Description
- Date of onset
- End date
- Severity
- Assessment of relatedness to study medication
- Other suspect drug or device
- Action taken

© Copyright: The University of Oxford and Oxford University Hospitals NHS Foundation Trust 2019

Follow-up information should be provided as necessary.

SAEs must be reported to the PC-CTU within 24 hours of discovery or notification of the event. The PC-CTU will acknowledge receipt of the SAE Report Form using the PC-CTU 'SAE Form Receipt' document. This receipt will be emailed or faxed to the site physician. If the site physician does not receive a receipt within 24hrs of them sending the report (during office hours), they should re-send the SAE Report Form to the PC-CTU by email or fax and telephone ahead.

The documentation will be reviewed by the Trial team. Following the initial check of the report, any additional information will be requested, and the CI or their medically qualified designated representative will review and evaluate the report for, causality and expectedness. In the event of a SUSAR the reporting timelines stated below will be followed. If there have been two assessments of causality made, the site physician's assessment cannot be downgraded. Where there is a discrepancy the worst case assessment is used for reporting purposes.

The PC-CTU will also ensure that SAE reports are reviewed by the Data Monitoring and Ethics Committee (DMEC), at their regular meetings.

Additional information, as it becomes available, will also be reported on the SAE Report Form (i.e. updating the original form) and returned to the PC-CTU by email or fax as above. The SAE Report Form will be filed in the Trial Master File according to PC-CTU_SOP_TM112 'Trial Master File and associated files', with copies filed in the patient's notes, the Case Record Form file and the Investigator Site File.

Any pregnancy occurring during the clinical trial and the outcome of the pregnancy will be recorded and followed up for congenital abnormalities or birth defects until the end of the trial at which point standard care will recommence.

Trial Managers log all SAEs reported. One of the metrics contained within this reporting is the number of SAEs reported and the cumulative number of SAEs for each study. Any concerns identified will be immediately raised with the Chief Investigator and may be tabled for discussion at the regular PC-CTU Management Committee meetings or referred to the study's DMEC for review. The DMEC also monitors the frequency and pattern of events reported as part of its independent oversight of the trial.

11.5.1. Events exempt from immediate reporting as SAEs

All SAEs will be reported to the PC-CTU from randomisation until 30-days after the participants' last dose/ last visit (as above)

11.5.2. Procedure for immediate reporting of Serious Adverse Events

- Site study team will complete an SAE report form for all reportable SAEs.
- The SAE report form will be scanned and emailed to the main trial team at the Primary Care Trials Unit, NDPCHS, Oxford immediately i.e., within 24 hours of site study team becoming aware of the event.
- Site study team will provide additional, missing or follow up information in a timely fashion.

11.6. Expectedness

Expectedness will be determined according to the approved RSI, i.e. Summary of Product Characteristics.

11.7. SUSAR Reporting

In collaboration with the PC-CTU, CTRG and DMEC, the Trial Management group will report all SUSARs to

© Copyright: The University of Oxford and Oxford University Hospitals NHS Foundation Trust 2019

the Competent Authorities (MHRA in the UK), the Research Ethics Committee concerned and Host NHS Trusts.

All SUSARs will be reported electronically to the MHRA within the timelines defined in the Medicines for Human Use (Clinical Trials) Regulations 2004 (as amended) using the following e-SUSAR reporting link via the MHRA website: https://esusar.mhra.gov.uk/

To use the e-SUSAR link, a copy of the MHRA approval letter for a new trial should be sent to the Sponsor so that they can log the trial on to the e-SUSAR system that they maintain. The Sponsor will then send the CI, and anyone else that has been nominated, the log-in password and details for them to view the trial and report SUSARs directly on to the system.

A fatal or life-threatening SUSAR is reported as soon as possible to the MHRA, the competent authorities of any EEA State other than the United Kingdom in which the trial is being conducted, and the relevant Ethics Committee not later than 7 days after the Sponsor was first aware of the reaction. Any additional relevant information should be reported within 8 days of the initial report.

A SUSAR which is not fatal or life-threatening is reported as soon as possible and in any event not later than 15 days after the PC-CTU is first aware of the reaction.

The Trial Management group will also inform all members of the Research Team concerned of relevant information about SUSARs that could adversely affect the safety of participants.

Further details are available at https://esusar.mhra.gov.uk/about/.

11.8. Development Safety Update Reports

In addition to the expedited reporting above, the CI shall submit once a year, throughout the clinical trial within 60 days of the date of the anniversary of the CTA or on request, a Developmental Safety Update Report to the Competent Authority (MHRA in the UK), Ethics Committee, Host NHS Trust and sponsor in line with PC-CTU_SOP_TM119 “Pharmacovigilance”.

If approved under the notification scheme, the HRA Annual Progress Report (APR) form should be used as a template for the DSUR, and should include a list of all SARs in Section 6. The cover letter must state that this is an APR in lieu of a full DSUR, and include the EudraCT number and CTA reference number.

For assessment of SARs in the DSUR, the RSI that was approved at **the start of the safety reporting period** will be used. When there has been approved changes to the RSI by substantial amendment during the reporting period, the RSI used for the DSUR will differ to the RSI used to assess expectedness at the time of SAR occurrence for SARs which require expedited reporting.

12. STATISTICS

12.1. Statistical Analysis Plan (SAP)

The statistical aspects of the study are summarised here with details fully described in a statistical analysisplan The SAP will be finalised before any analysis takes place.

© Copyright: The University of Oxford and Oxford University Hospitals NHS Foundation Trust 2019

12.2. Description of Statistical Methods

In accordance with CONSORT guidelines, we will record and report participant flow. Descriptive statistics of recruitment, drop-out, and completeness of interventions will be provided. Baseline variables will be presented by randomised group using frequencies (with percentages) for binary and categorical variables and means (and standard deviations) or medians (with lower and upper quartiles) for continuous variables. There will be no tests of statistical significance nor confidence intervals for differences between groups on any baseline variables.

The primary analysis will be on an intention-to-treat basis. That is, after randomisation, participants will be analysed according to their allocated treatment group irrespective of what treatment they actually receive. The primary outcome will be analysed using a survival analysis method, such as Cox proportional-hazards model, adjusting for practices. Results will be presented as hazard ratios with 95% confidence intervals and associated two-sided P-values. To test the robustness of the result, a sensitivity analysis will be carried out, using the same method, adjusting the following pre-specified baseline prognostic factors: diastolic and/or systolic blood pressure above or below NICE target, type II diabetes and coronary artery disease.

Same approach will be repeated for individual components of the primary composite endpoint and all-cause mortality as secondary analyses. Analyses for other outcomes will be carried out using multiple log-binomial regression models for binary data and linear mixed effect model for continuous data collected over time.

Assumption of proportional hazards will be examined and if any of the assumptions were violated, a suitable alternative survival method will be considered. Similarly, alternative methods will be consideredif any violation of assumptions is detected in any of the aforementioned methods for other outcomes.

Adverse effects will be tabulated according to randomised group assignments and the proportions will be compared using Fisher's exact test.

A full detailed analysis plan, including approach of handling missing data, subgroup analyses, and sensitivity analyses, and a plan for interim analysis will be prepared before the final datalock and analysis by a statistician who is independent from the study. All analyses will be performed by the trial statistician and validated bya separate statistician.

12.3. Sample Size Determination

A UK representative spread of practices will be achieved by stratifying practice postcode location into quartiles of Townsend Deprivation Score and selecting practices that agree to take part sequentially until each deprivation quartile practice target is reached. This strategy will most probably ensure that populations selected will also be representative for ethnicity but the sequential practice selection strategy will be examined after ten practices have been selected for each deprivation quartile to ensure practices serving high proportions of ethnic minorities are including in the final five places, if this has not already occurred in the earlier selections.

The estimate for the cardiovascular (CV) event rate (defined by hospitalisation for coronary heart disease, heart failure, ischemic stroke and peripheral arterial disease) and total mortality rate in patients with CKD 3b (eGFR 30-44 ml/min/1.73m^2) being 11.29 and 4.76 per 100 person years respectively gives a combined

© Copyright: The University of Oxford and Oxford University Hospitals NHS Foundation Trust 2019

event rate of 16.05 per 100 person years [5]. In those with eGFR in the range 45-50 ml/min/1.73m^2, the event rate is conservatively estimated to be 0.667 times as high (10.7 events per 100 person years[76] and we assume half the participants will fall in this range giving an overall event rate of 13.4 events per 100 person years. To detect a 20% relative risk reduction in death or cardiovascular events within 3 years in the intervention group as compared with the control group (i.e. hazard ratio=0.8) with an anticipated treatment withdrawal rate of 13% (which gives a diluted estimated treatment effect = 0.84) and a two sided significance of 0.05, 1511 participants per group (3022 total) are required at 80% power and assuming 10% attrition rate.

We have decided to power the trial conservatively on a 20% risk reduction since this proposed treatment effect is around half the risk reduction observed in the ARA mild heart failure trial (EMPHASIS). The estimated hazard ratio in the EMPHASIS eplerenone versus placebo mild heart failure trial (only mildly symptomatic patients were included) were 0.63 (CI 0.54-0.74, p<0.001) for the composite endpoint of death from CV causes or hospitalisation for heart failure at the median follow up of 21 months. The conservative upper CI for the treatment effect was 26% reduction. The placebo CV event rate in EMPHASIS trial was similar to observational data on CV events in CKD 3b patients [5].

12.4. Analysis Populations

The primary analysis will be carried out on all randomised participants, on an Intention to treat (ITT) basis, assuming non-informative censoring for those withdrawn or lost to follow-up. Based in the ITT principle, participants who are withdrawn from treatment (e.g. for safety reasons) but consent to continue follow-up will be included in the ITT analysis. Participants who are withdrawn or lost to follow-up will be censored at the date of withdrawal or date of last follow-up, respectively. All participants will be analysed in the groups to which they were allocated, regardless of treatment compliance.

The safety population will include all participants who received the study medication.

12.5. Decision Points

There will be no interim analysis performed to assess treatment efficacy. The statisticians who prepares the DMEC reports and the DMEC assess the closed session reports with treatment allocation being blinded. All results of the closed report will only be available to the trial statisticians and the DMEC.

12.6. Stopping Rules

A formal futility analysis will be performed and fully reviewed by the DMEC.

12.7. The Level of Statistical Significance

5% significance level (2-sided)

12.8. Procedure for Accounting for Missing, Unused, and Spurious Data.

The missing at random assumption will be tested as far as is possible by analysing each baseline covariate in a regression model to determine which if any are associated with missingness.

All baseline covariates are expected to be observed. Baseline values will be summarised for those who did

© Copyright: The University of Oxford and Oxford University Hospitals NHS Foundation Trust 2019

and did not complete follow up measurements to describe any characteristics related to missingness that are able to be observed.

We will be analysing our data using an intention to treat analysis. All randomised patients will be included in the analysis, assuming non-informative censoring for those withdrawn from the study or lost to follow-up for the primary analysis.

During statistical data review and analysis, any anomalies in the data will be investigated and discussed with the trial management team. The data investigation will be broad and flexible and focus on variability of the data, consistency, dispersion, outliers, inliers, relationships between variables and relationships over time. The statistical data review will be fully documented with allthe output dated. If fraud is proved, fraudulent data will be removed from the analysis.

12.9. Procedures for Reporting any Deviation(s) from the Original Statistical Plan

We do not anticipate any major deviation from the Statistical Analysis Plan. However, provision for alternative methods and changes to analyses will be included in the Statistical Analysis Plan as specified in PC-CTU_SOP_ST104 “Statistical Report”.

12.10. Health Economics Analysis

A health economic analysis will be integrated into the trial.

Research Question: What is the cost-effectiveness of adding an ARA to usual care in CKD3b?

Data collection: The cost analysis will adopt an NHS perspective. Data on health care resource use will be collected from all trial patients, including all relevant hospital and GP consultations, medications, referrals, tests and equipment. Protocol-driven costs will be omitted. Where possible data on resource utilisation will be collected from electronic patient records, although it is likely that some resources will not be routinely documented in electronic format and data extraction from the medical notes will be supplemented by self-reported resource utilisation diaries filled out by the patients. Patients will be asked to complete the diaries for the period from weeks 0-12, 13-26 and every 13 weeks up to 152 weeks in which we will ask them to identify and record items relating to utilisation of any other relevant health care resources and patient burden, including time off work and foregone leisure and productivity time (i.e. absenteeism).

Where possible, we will value our items on health care resource utilisation using appropriate unit costs obtained from published sources, including the most recent version of Unit Costs of Health and Social Care and NHS Reference Costs. We will estimate unit costs which are not available from secondary sources using the approach used in the most recent version of Unit Costs of Health and Social Care.

Primary endpoint data will be collected within the trial. NICE recommends the use of preference-based health-related quality of life (HRQL) measures for the purpose of determining Quality Adjusted Life Years (QALYs) for economic evaluation. The use of quality-adjusted life years aims to capture the impact of disease progression and non-fatal events on quality of life in addition to any impact on survival. The EQ-5D-5L will be used to measure patient health-related QoL at baseline, 6 months, 12 months and annually thereafter. Patient’s 5-dimension (mobility, self-care, usual activities, pain/discomfort, anxiety and depression) EQ-5D-5L health state classification at each trial time point will be converted into a utility score on a 0 to 1 scale where 0 is equivalent to dead, and 1, to perfect health. This conversion will be made using the new algorithm based on the UK value set currently being conducted by the EuroQol Group, if available

© Copyright: The University of Oxford and Oxford University Hospitals NHS Foundation Trust 2019

at the time of analysis. If not available the current crosswalk algorithm provided by the EuroQolgroup and algorithm estimated by Dolan et al. derived from a survey of the UK population (n=3337) [71], will be used. Utility values in the tariff set range from no problems on any of the five dimensions in the EQ-5D-5L descriptive system (value=1.0) to severe or extreme problems across all five dimensions (value=−0.594) [71, 72]. The utility scores will be combined with within-trial survival data to estimate the quality adjusted life-years (QALY's) required for the cost-utility analysis.

Adding an ARA to usual care in the CKD3b population may improve the patient's overall quality of life which goes beyond health. The ICEpop CAPability measure for Adults (ICECAP-A), and Quality of Life Visual Analogue Scale (QoL VAS) will be used to measure CKD patient's overall quality of life, initially at baseline, 6 months, 12 months in a sub-population of the trial, and annually thereafter as funding permits. ICECAP-A is a self-reported measure of capability in adults (over 18 years). The measure covers attributes of wellbeing that were found to be important to adults in the UK. ICECAP-A comprises five attributes: settlement (feeling settled and secure), attachment (love, friendship and support), control (independence), role (achievement and progress), and enjoyment (enjoyment and pleasure); and each factor has four response levels. Index values have been estimated using a best-worst scaling technique. QoL VAS is a vertical line from 0 to 100 with anchors of best and worst imaginable life for people to report their perceived quality of life today. Together with KDQOL-SF and EQ-5D, ICECAP-A and QoL VAS will provide a full picture of treatment effect on CKD, general health and overall quality of life.

Analysis:

Missing data

The resource-use/cost and EQ-5D-5L data will be investigated to ascertain the extent of missing data and whether it is missing at random or not at random and/or censoring. If deemed to be required and missing at random, we will conduct multiple imputation using standard methods [73, 74].

Analysis of healthcare resource use, cost and EQ-5D-5L data

The focus of studying the healthcare resource use is to investigate how ARA plus routine care in CKD patients affects the health care costs. With the aim of the economic analysis to estimate how the costs of the intervention minus the difference in health care costs between the intervention and routine care group of patients balances against the health care benefits. A two-stage analysis of the healthcare resource use and their costs will be conducted. First the impact of the intervention on (1) all healthcare resource use/costs, (2) kidney disease specific healthcare resource use/costs, and (3) CVD related healthcare resources costs will be evaluated over the duration of the study (36-month period). Secondly, a regression framework that relates healthcare costs to baseline characteristics (age and gender), kidney disease stage, progression, other co-morbidities and CVD will be developed. The objective being to provide estimates of healthcare costs for different kidney disease stages and CVD events to inform the extrapolation model (see below). A similar regression framework approach will be used for the EQ-5D-5L tariff data at the different data collection time-points, again to inform the extrapolation model.

Within-trial cost-effectiveness analysis

The economic evaluation will compare the implementation of ARA plus routine care with routine care for CKD patients. We plan to conduct a within-trial economic analysis, then if the trial demonstrates clinical effectiveness, these within trial results will be used to extrapolate beyond the trial endpoint and model the likely life-time cost-effectiveness.

© Copyright: The University of Oxford and Oxford University Hospitals NHS Foundation Trust 2019

A within-trial cost-consequence analysis will initially be reported, describing all the important results relating to the health care resource use, costs and consequences of ARA plus routine care compared with routine care for CKD patients. Subsequently, a within-trial cost-effectiveness analysis will consider cost per additional primary endpoint (mortality and onset of or hospitalisation for of CVD) averted, and a cost-utility analysis will determinecost per quality-adjusted life year (QALY) gained. The use of QALY's aims to capture the impact of diseaseprogression and non-fatal events on health-related quality of life in addition to any impact on survival. Discounting at a rate of 3.5% will be applied. Results will be expressed in terms of incremental cost- effectiveness ratios (ICERs). Sensitivity analysis will test the robustness of the results. This will explore uncertainties in the trial-based data itself, the methods employed to analyse the data and the generalisability of the results to other settings, to determine the impact of changes on results. Non-parametric bootstrapping and probabilistic sensitivity analysis will explore uncertainty in the confidence placed on the results of the economic analysis and cost effectiveness acceptability curves will be presented.

Lifetime cost-effectiveness analysis:

If trial results demonstrate clinical effectiveness, extrapolation beyond the trial period of 36 months will be undertaken. The methods used will depend on the within trial data, but will either use parametric methods as set out by the NICE Decision Support Unit [74] or use a lifetime decision-model (developing a Markov model or adapting a CKD model that is currently being developed by researchers in HERC for the SHARP trial http://www.ctsu.ox.ac.uk/~sharp/) in order to determine the long-term cost-effectiveness of the intervention in terms of cost per QALY gained. This will be based on the individual patient data (using the results from the regression analyses outlined above) from the study and external data (where required). It will be carried out from an NHS and Personal Social Services perspective, to take into account health care costs and longer term social care costs of cardiovascular events and the impact on life expectancy, quality adjusted life expectancy. The model will be run over remaining patient lifetime, with costs and benefits discounted at a rate of 3.5%. The lifetime cost-effectiveness analysis will be driven by the decision analytic model and the way treatment effects are propagated in the model. Extensive deterministic sensitivity analysis will be undertaken to assess the impact of changing the values of key parameters and will be used to explore the importance of modelling assumptions. Probabilistic sensitivity analyses will be conducted to deal with uncertainty in model parameters and cost-acceptability curves presented.

13. DATA MANAGEMENT

The data management aspects of the study are summarised here with details fully described in the Data Management Plan.

13.1. Source Data

Source documents will include:

- Primary care electronic and paper records/outputs
- Reports from laboratory investigations
- Hospital correspondence
- Records of 24 hour ambulatory and home BP measurements

© Copyright: The University of Oxford and Oxford University Hospitals NHS Foundation Trust 2019

- Patient questionnaires
- Patient diary cards
- The CRF itself where there is no other written or electronic record of data

All Study Data Documents (SDDs) in paper format are date stamped upon receipt and tracked within a trial management database. A full pre-entry review ensures that all pages have been received, IDs are consistent and obvious errors/missing data are appropriately addressed prior to entry. All SDDs are double entered by two independent staff into the clinical database.

Data validation for all data entered into the clinical database, either manually, is achieved by programming study specific checks or through manual review of listing outputs. All discrepancies generated by electronic validation checks or manual listings are reviewed by the Clinical Data manager. If clarification from a Research Site is required, the query is added to a Data Verification Site (DVS) Report, and subsequently issued. The Clinical Data Manager oversees the tracking of DVS reports until they are resolved, and application of any updates to the clinical database. Query status is tracked and monitored within the clinical database and feedback is provided regularly to the trial management team.

All documents will be stored safely in confidential conditions according to PC-CTU policies and SOPs. On all study-specific documents, other than the signed consent and contact details form, the participant will be referred to by the study participant number/code, not by name. Study documentation will be archived for a period of 5 years according to PC-CTU_SOP_TM124 “Archiving”.

Source data will be verified as appropriate by the PC-CTU Quality Manager or delegate using a risk based approach and will be defined in the monitoring plan.

13.2. Access to Data

Direct access will be granted to authorised representatives from the sponsor, host institution (Oxford University BARACK D Research Team) and the regulatory authorities to permit trial-related monitoring, audits and inspections. Individual GP practices/specialist renal groups will be required to give access to those bodies described above and this will be outlined in the Site Agreement.

13.3. Data Recording and Record Keeping

All Data Management functions will be performed in line with PC-CTU_SOP_DM101 “Data Management”. A Data Management Plan (DMP) is in place for all PC-CTU studies outlining in detail the study specific procedures that are in place to ensure that high quality data are produced for statistical analysis. The DMP is reviewed and signed by all applicable parties including the Trial Manager and the Trial Statistician prior to the first patient being enrolled.

Clinical trial data is collected by the PC-CTU both electronically and in paper format. All Study Data Documents (SDDs) in paper format are date stamped upon receipt and tracked within a trial management database. A full pre-entry review ensures that all pages have been received, IDs are consistent and obvious errors/missing data are appropriately addressed prior to entry. All SDDs are double entered by two independent staff into the clinical database.

Data validation for all data entered into the clinical database, either manually or by electronic data capture from site, is achieved by programming study specific checks or through manual review of listing outputs. All discrepancies generated by electronic validation checks or manual listings are reviewed by the Clinical

© Copyright: The University of Oxford and Oxford University Hospitals NHS Foundation Trust 2019

Data manager. If clarification from a Research Site is required, the query is added to a Data Verification Site (DVS) Report, and subsequently issued. The Clinical Data Manager oversees the tracking of DVS reports until they are resolved, and application of any updates to the clinical database. Query status is tracked and monitored within the clinical database and feedback is provided regularly to the trial management team.

Prior to database lock, dataset review is undertaken by the Information System Manager and the Trial Statistician. All critical data items are 100% checked against original SDDs to ensure accuracy and an error rate is established across all fields to ensure a consistently accurate dataset.

An independent review of the quality of the data being produced by each PC-CTU trial is provided by its Data Monitoring and Ethics Committee throughout the study.

14. QUALITY ASSURANCE PROCEDURES

14.1. Risk assessment

The study will be conducted in accordance with the current approved protocol, ICH GCP, relevant regulations and PC-CTU Standard Operating Procedures. The PC-CTU has in place procedures for assessing risk management for trials which will outline the monitoring required. The monitoring will be carried out by the PC-CTU Quality Assurance Manager or equivalent. The investigators and all trial related site staff will receive appropriate training in Good Clinical Practice and trial procedures.

14.2. Monitoring

Regular monitoring will be performed according to ICH GCP using a risk based approach. Data will be evaluated for compliance with the protocol and accuracy in relation to source documents where possible. Following written standard operating procedures, the team will verify that the clinical trial is conducted and data are generated, documented and reported in compliance with the protocol, GCP and the applicable regulatory requirements. The Study Monitor may also assess SAE's.

The PC-CTU Trial Management Committee will be responsible for the monitoring of all aspects of the trial's conduct and progress and will ensure that the protocol is adhered to and that appropriate action is taken to safeguard participants and the quality of the trial itself. The TMC will be comprised of individuals responsible for the trial's day to day management (e.g. the CI, trial manager, statistician, data manager) and will meet regularly throughout the course of the trial

14.3. Trial committees

A Trial Steering Committee (TSC) will be convened to provide overall supervision of the trial and ensure its conduct is in accordance with the principles of GCP and the relevant regulations. The role of a Trial Steering Committee is to provide overall supervision of the trial and ensure that it is being conducted in accordance with the principles of GCP and the relevant regulations. The Trial Steering Committee will agree the trial protocol and any protocol amendments and provide advice to the investigators on all aspects of the trial. The TSC will consist of members who are independent of the investigators, in particular, an independent chairperson.

14.3.1 Safety Monitoring Committee

An independent Data Monitoring and Ethics Committee (DMEC) will review the accruing trial andsafety data to ensure trial site staff and participants are aware of any relevant safety informationand to determine whether any reasons exist for the trial to be discontinued.

© Copyright: The University of Oxford and Oxford University Hospitals NHS Foundation Trust 2019

15. PROTOCOL DEVIATIONS

A trial related deviation is a departure from the ethically approved trial protocol or other trial document or process (e.g. consent process or IMP administration) or from Good Clinical Practice (GCP) or any applicable regulatory requirements. Any deviations from the protocol will be documented in a protocol deviation form and filed in the trial master file, as per SOP PC-CTU_SOP_TM125 "Incident Reporting, Protocol deviations and Serious Breaches"

16. SERIOUS BREACHES

The Medicines for Human Use (Clinical Trials) Regulations contain a requirement for the notification of "serious breaches" to the MHRA within 7 days of the Sponsor becoming aware of the breach.

A serious breach is defined as "A breach of GCP or the trial protocol which is likely to affect to a significant degree –

(a) the safety or physical or mental integrity of the subjects of the trial; or
(b) the scientific value of the trial".

In the event that a serious breach is suspected the Sponsor must be contacted within 1 working day. In collaboration with the CI the serious breach will be reviewed by the Sponsor and, if appropriate, the Sponsor will report it to the REC committee, Regulatory authority and the relevant NHS host organisation within seven calendar days.

Possible serious breaches may be identified by members of the study team through various means including meetings, site monitoring and audit visits. Members of the team may also receive allegations of serious breach of GCP directly or indirectly from whistle blowers or complainants from within or outside the University. Information in written form will be retained and where communication is verbal, study staff will generate a written record. The possible breach will then be recorded and discussed with the relevant trial team members.

Information regarding possible serious breaches will be treated as confidential with details being released to staff on a need-to-know basis. All individuals interviewed during the investigation will be expected to respect this confidentiality. A specific folder will be created both electronically and within the TMF and will include all relevant documentation and copies of emails, referencing the addressee, the date and time of the email. Once information has been received, the subsequent procedure will be followed:

- Data will be collated
- The study team, involving relevant staff e.g. QA manager, will review MHRA guidance to assess whether the event constitutes a serious breach. All relevant information and decisions made shall be recorded on the PC-CTU Serious Breaches Assessment Form.
- If, following assessment, the event is considered a serious breach, the CI will confirm the decision and contact CTRG. Day 1 as regards to the reporting timelines will be from the agreement of the characterisation between the CI and CTRG. The event will then be reported to the MHRA by the head of CTRG or delegate within seven days and provide follow-up to the CI and study team.
- PC-CTU staff will immediately review the related documentation and systems to assess the possible cause or systemic failure in order to inform an action plan.

A Corrective Action Preventative Action Plan will be drawn up by the study team in collaboration with CTRG.

© Copyright: The University of Oxford and Oxford University Hospitals NHS Foundation Trust 2019

17. ETHICAL AND REGULATORY CONSIDERATIONS

17.1. Declaration of Helsinki

The Investigator will ensure that this trial is conducted in accordance with the principles of the Declaration of Helsinki.

17.2. Guidelines for Good Clinical Practice

The Investigator will ensure that this trial is conducted in accordance with relevant regulations and with Good Clinical Practice.

17.3. Approvals

Following Sponsor approval the protocol, informed consent form, participant information sheet and any proposed advertising material will be submitted to an appropriate Research Ethics Committee (REC), the Health Research Authority (HRA), the Medicines and Healthcare Regulatory Authority (MHRA in the UK), the relevant NHS Research and Development Departments and host institution for written approval. The Research Team will submit and, where necessary, obtain approval from the above parties for all substantial amendments to the original approved documents.

17.4. Other Ethical Considerations

We do not believe that there are any significant ethical issues related to this trial. Site staff will be fully trained in GCP according to their study role.

17.5. Reporting

The CI shall submit once a year throughout the clinical trial, or on request, an Annual Progress Report to the REC, HRA (where required), host organisation, funder (where required) and Sponsor. In addition, an End of Trial notification and final report will be submitted to the MHRA, the REC, host organisation and Sponsor.

17.6. Transparency in Research

Prior to the recruitment of the first participant, the trial will have been registered on a publicly accessible database.

Results will be uploaded to the European Clinical Trial (EudraCT) Database within 12 months of the end of trial date as given on the trial declaration (6 months for paediatric trials) by the CI or their delegate.

Where the trial has been registered on multiple public platforms, the trial information will be kept up to date during the trial, and the CI or their delegate will upload results to all those public registries within 12 months of the end of the trial declaration.

17.7. Participant Confidentiality

Ensuring patient confidentially is an established and robust process within the PC-CTU. All Staff adhere to the principles of Good Clinical Practice (GCP) and the Data Protection Act, 2018.

© Copyright: The University of Oxford and Oxford University Hospitals NHS Foundation Trust 2019

It is the PC-CTU's preferred procedure that patients will only be identified on study documents by use of a unique study ID which cannot be used to identify individual participants. Where this is not possible specific consent will be taken and participants contact details will be used, in order of their preference e.g. when necessary to make follow-up phone calls or emails. All study documents such as case report forms (CRFs) holding patient information are held securely with restricted access either electronically or in paper format.

CRFs and all other documents holding identifiers are anonymised as soon as possible with the process of management being outlined in detail within the ethics application and in trial specific procedures. The holding of patient identifiers is noted as a trial specific vulnerability in the risk assessment and the Chief Investigator (CI) is required to clearly outline how such risks will be managed, to minimise both likelihood and impact and how the success of the management will be monitored and assessed.

Patients will be flagged for long term follow up for mortality, kidney function and cardiovascular outcomes, collected via the RCGP network. Consent for this activity is included in the consent form.

The study will comply with the General Data Protection Regulation (GDPR) and Data Protection Act 2018, which require data to be de-identified as soon as it is practical to do so. The processing of the personal data of participants will be minimised by making use of a unique participant study number only on all study documents and any electronic database(s). All documents will be stored securely and only accessible by study staff and authorised personnel. The study staff will safeguard the privacy of participants' personal data.

17.8. Expenses and Benefits

Reasonable travel expenses for any visits additional to normal care will be reimbursed on production of receipts, or a mileage allowance provided as appropriate.

18. FINANCE AND INSURANCE

18.1. Funding

The trial is funded by the National Institute for Health Research Health Technology Assessment Programme.

18.2. Insurance

The University has a specialist insurance policy in place which would operate in the event of any participant suffering harm as a result of their involvement in the research (Newline Underwriting Management Ltd, at Lloyd's of London). NHS indemnity operates in respect of the clinical treatment that is provided.

18.3. Contractual arrangements

Appropriate contractual arrangements will be put in place with all third parties.

19. PUBLICATION POLICY

The Investigators will be involved in reviewing drafts of the manuscripts, abstracts, press releases and any other publications arising from the study. Authors will acknowledge that the study was funded by the National Institute for Health Research Health Technology Assessment Programme. Authorship will be

© Copyright: The University of Oxford and Oxford University Hospitals NHS Foundation Trust 2019

determined in accordance with the ICMJE guidelines and other contributors will be acknowledged

20. DEVELOPMENT OF A NEW PRODUCT/ PROCESS OR THE GENERATION OF INTELLECTUAL PROPERTY

Ownership of IP generated by employees of the University vests in the University. The University will ensure appropriate arrangements are in place as regards any new IP arising from the trial.

21. ARCHIVING

Study materials will be archived according to the PC-CTU Standard Operating Procedure on Archiving and kept for at least 5 years following the publication on long term outcomes.

22. REFERENCES

1. Levey, A.S., et al., *A more accurate method to estimate glomerular filtration rate from serum creatinine: a new prediction equation. Modification of Diet in Renal Disease Study Group.* Ann Intern Med, 1999. **130**(6): p. 461-70.

2. Hallan, S.I., et al., *Screening strategies for chronic kidney disease in the general population: follow-up of cross sectional health survey.* BMJ, 2006. **333**(7577): p. 1047.

3. Coresh, J., et al., *Prevalence of chronic kidney disease and decreased kidney function in the adult US population: Third National Health and Nutrition Examination Survey.* American Journal of Kidney Diseases, 2003. **41**(1): p. 1-12.

4. Keith, D.S., et al., *Longitudinal follow-up and outcomes among a population with chronic kidney disease in a large managed care organization.* Arch Intern Med, 2004. **164**(6): p. 659-63.

5. Go, A.S., et al., *Chronic kidney disease and the risks of death, cardiovascular events, and hospitalization.* N Engl J Med, 2004. **351**(13): p. 1296-305.

6. Matsushita, K., et al., *Association of estimated glomerular filtration rate and albuminuria with all-cause and cardiovascular mortality in general population cohorts: a collaborative meta-analysis.* Lancet, 2010. **375**(9731): p. 2073-81.

7. Coresh, J., et al., *Prevalence of chronic kidney disease in the United States.* JAMA, 2007. **298**(17): p. 2038-47.

8. Abramson, J.L., et al., *Chronic kidney disease, anemia, and incident stroke in a middle-aged, community-based population: the ARIC Study.* Kidney Int, 2003. **64**(2): p. 610-5.

9. Van Biesen, W., et al., *The glomerular filtration rate in an apparently healthy population and its relation with cardiovascular mortality during 10 years.* Eur Heart J, 2007. **28**(4): p. 478-83.

10. Shlipak, M.G., et al., *Cystatin C and the risk of death and cardiovascular events among elderly persons.* N Engl J Med, 2005. **352**(20): p. 2049-60.

11. Tonelli, M., et al., *Chronic Kidney Disease and Mortality Risk: A Systematic Review.* Journal of the

© Copyright: The University of Oxford and Oxford University Hospitals NHS Foundation Trust 2019

American Society of Nephrology, 2006. **17**(7): p. 2034-2047.

12. Foley, R.N., et al., *Left ventricular hypertrophy in new hemodialysis patients without symptomatic cardiac disease.* Clin J Am Soc Nephrol, 2010. **5**(5): p. 805-13.

13. Foley, R.N., et al., *Chronic kidney disease and the risk for cardiovascular disease, renal replacement, and death in the United States Medicare population, 1998 to 1999.* J Am Soc Nephrol, 2005. **16**(2): p. 489-95.

14. Collins, A.J., et al., *Chronic kidney disease and cardiovascular disease in the Medicare population.* Kidney Int Suppl, 2003(87): p. S24-31.

15. Mark, P.B., et al., *Redefinition of uremic cardiomyopathy by contrast-enhanced cardiac magnetic resonance imaging.* Kidney Int, 2006. **69**(10): p. 1839-45.

16. Foley, R.N., et al., *Clinical and echocardiographic disease in patients starting end-stage renal disease therapy.* Kidney Int, 1995. **47**(1): p. 186-92.

17. Edwards, N.C., et al., *Aortic distensibility and arterial-ventricular coupling in early chronic kidney disease: a pattern resembling heart failure with preserved ejection fraction.* Heart, 2008. **94**(8): p. 1038-43.

18. London, G.M., et al., *Cardiac and arterial interactions in end-stage renal disease.* Kidney Int, 1996. **50**(2): p. 600-8.

19. Covic, A., et al., *Analysis of the effect of hemodialysis on peripheral and central arterial pressure waveforms.* Kidney Int, 2000. **57**(6): p. 2634-43.

20. Matsumoto, Y., M. Hamada, and K. Hiwada, *Aortic distensibility is closely related to the progression of left ventricular hypertrophy in patients receiving hemodialysis.* Angiology, 2000. **51**(11): p. 933-41.

21. Chue, C.D., et al., *Arterial stiffness in chronic kidney disease: causes and consequences.* Heart, 2010. **96**(11): p. 817-23.

22. London, G.M., et al., *Arterial wave reflections and survival in end-stage renal failure.* Hypertension, 2001. **38**(3): p. 434-8.

23. Guerin, A.P., et al., *Impact of aortic stiffness attenuation on survival of patients in end-stage renal failure.* Circulation, 2001. **103**(7): p. 987-92.

24. Muntner, P., et al., *Traditional and nontraditional risk factors predict coronary heart disease in chronic kidney disease: results from the atherosclerosis risk in communities study.* J Am Soc Nephrol, 2005. **16**(2): p. 529-38.

25. Mourad, J.J., et al., *Creatinine clearance, pulse wave velocity, carotid compliance and essential hypertension.* Kidney Int, 2001. **59**(5): p. 1834-41.

26. Baigent, C., et al., *The effects of lowering LDL cholesterol with simvastatin plus ezetimibe in patients with chronic kidney disease (Study of Heart and Renal Protection): a randomised placebo-controlled trial.* Lancet, 2011. **377**(9784): p. 2181-92.

© Copyright: The University of Oxford and Oxford University Hospitals NHS Foundation Trust 2019

27. Casas, J.P., et al., *Effect of inhibitors of the renin-angiotensin system and other antihypertensive drugs on renal outcomes: systematic review and meta-analysis.* Lancet, 2005. **366**(9502): p. 2026-33.

28. SOLVD Investigators, *Effect of enalapril on survival in patients with reduced left ventricular ejection fractions and congestive heart failure. The SOLVD Investigators.* N Engl J Med, 1991. **325**(5): p. 293-302.

29. Yusuf, S., et al., *Effects of an angiotensin-converting-enzyme inhibitor, ramipril, on cardiovascular events in high-risk patients. The Heart Outcomes Prevention Evaluation Study Investigators.* N Engl J Med, 2000. **342**(3): p. 145-53.

30. Brown, N.J., *Eplerenone: cardiovascular protection.* Circulation, 2003. **107**(19): p. 2512-8.

31. Funder, J.W. and A.S. Mihailidou, *Aldosterone and mineralocorticoid receptors: Clinical studies and basic biology.* Mol Cell Endocrinol, 2009. **301**(1-2): p. 2-6.

32. Struthers, A.D., *Aldosterone: cardiovascular assault.* Am Heart J, 2002. **144**(5 Suppl): p. S2-7.

33. Robert, V., et al., *Biological determinants of aldosterone-induced cardiac fibrosis in rats.* Hypertension, 1995. **26**(6 Pt 1): p. 971-8.

34. Rocha, R., et al., *Aldosterone: a mediator of myocardial necrosis and renal arteriopathy.* Endocrinology, 2000. **141**(10): p. 3871-8.

35. Rocha, R., et al., *Mineralocorticoid blockade reduces vascular injury in stroke-prone hypertensive rats.* Hypertension, 1998. **31**(1 Pt 2): p. 451-8.

36. Catena, C., et al., *Long-term cardiac effects of adrenalectomy or mineralocorticoid antagonists in patients with primary aldosteronism.* Hypertension, 2007. **50**(5): p. 911-8.

37. Milliez, P., et al., *Evidence for an increased rate of cardiovascular events in patients with primary aldosteronism.* J Am Coll Cardiol, 2005. **45**(8): p. 1243-8.

38. Palmer, B.R., et al., *Plasma aldosterone levels during hospitalization are predictive of survival post-myocardial infarction.* Eur Heart J, 2008. **29**(20): p. 2489-96.

39. Tomaschitz, A., et al., *Plasma aldosterone levels are associated with increased cardiovascular mortality: the Ludwigshafen Risk and Cardiovascular Health (LURIC) study.* Eur Heart J, 2010. **31**(10): p. 1237-47.

40. Pitt, B., et al., *The effect of spironolactone on morbidity and mortality in patients with severe heart failure. Randomized Aldactone Evaluation Study Investigators.* N Engl J Med, 1999. **341**(10): p. 709-17.

41. Pitt, B., et al., *Eplerenone, a selective aldosterone blocker, in patients with left ventricular dysfunction after myocardial infarction.* N Engl J Med, 2003. **348**(14): p. 1309-21.

42. Zannad, F., et al., *Eplerenone in patients with systolic heart failure and mild symptoms.* N Engl J Med, 2011. **364**(1): p. 11-21.

43. Hayashi, M., et al., *Immediate administration of mineralocorticoid receptor antagonist*

© Copyright: The University of Oxford and Oxford University Hospitals NHS Foundation Trust 2019

spironolactone prevents post-infarct left ventricular remodeling associated with suppression of a marker of myocardial collagen synthesis in patients with first anterior acute myocardial infarction. Circulation, 2003. **107**(20): p. 2559-65.

44. Zannad, F., et al., *Limitation of excessive extracellular matrix turnover may contribute to survival benefit of spironolactone therapy in patients with congestive heart failure: insights from the randomized aldactone evaluation study (RALES). Rales Investigators.* Circulation, 2000. **102**(22): p. 2700-6.

45. Navaneethan, S.D., et al., *Aldosterone antagonists for preventing the progression of chronic kidney disease.* Cochrane Database Syst Rev, 2009(3): p. CD007004.

46. Del Vecchio, L., et al., *Mechanisms of disease: The role of aldosterone in kidney damage and clinical benefits of its blockade.* Nat Clin Pract Nephrol, 2007. **3**(1): p. 42-9.

47. Remuzzi, G., D. Cattaneo, and N. Perico, *The aggravating mechanisms of aldosterone on kidney fibrosis.* J Am Soc Nephrol, 2008. **19**(8): p. 1459-62.

48. Epstein, M., *Aldosterone as a mediator of progressive renal disease: pathogenetic and clinical implications.* Am J Kidney Dis, 2001. **37**(4): p. 677-88.

49. Nagase, M., et al., *Podocyte injury underlies the glomerulopathy of Dahl salt-hypertensive rats and is reversed by aldosterone blocker.* Hypertension, 2006. **47**(6): p. 1084-93.

50. Jain, G., R.C. Campbell, and D.G. Warnock, *Mineralocorticoid receptor blockers and chronic kidney disease.* Clin J Am Soc Nephrol, 2009. **4**(10): p. 1685-91.

51. Bianchi, S., R. Bigazzi, and V.M. Campese, *Intensive versus conventional therapy to slow the progression of idiopathic glomerular diseases.* Am J Kidney Dis, 2010. **55**(4): p. 671-81.

52. Matsumoto, Y., et al., *Long-term low-dose spironolactone therapy is safe in oligoanuric hemodialysis patients.* Cardiology, 2009. **114**(1): p. 32-8.

53. Andrew S. Levey, M.J.P.B., MD; Julia Breyer Lewis, MD; Tom Greene, PhD; Nancy Rogers, MS; and David Roth, MD, for the Modification of Diet in Renal Disease Study Group, *A More Accurate Method To Estimate Glomerular Filtration Rate from Serum Creatinine: A New Prediction Equation.* Ann Intern Med, 1999. **130**: p. 461-47.

54. Andrew S. Levey, M.L.A.S., MD, MS; Christopher H. Schmid, PhD; Yaping (Lucy) Zhang, MS; Alejandro F. Castro III, MPH;, M. Harold I. Feldman, MSCE; John W. Kusek, PhD; Paul Eggers, PhD; Frederick Van Lente, PhD; Tom Greene, PhD; and, and M. Josef Coresh, PhD, MHS, for the CKD-EPI (Chronic Kidney Disease Epidemiology Collaboration)*, *A New Equation to Estimate Glomerular Filtration Rate.* Ann Intern Med, 2009. **150**: p. 604-612.

55. Stevens, L.A., et al., *Comparative Performance of the CKD Epidemiology Collaboration (CKD-EPI) and the Modification of Diet in Renal Disease (MDRD) Study Equations for Estimating GFR Levels Above 60 mL/min/1.73 m2.* American Journal of Kidney Diseases, 2010. **56**(3): p. 486-495.

56. Levey, A.S. and L.A. Stevens, *Estimating GFR using the CKD Epidemiology Collaboration (CKD-EPI) creatinine equation: more accurate GFR estimates, lower CKD prevalence estimates, and better risk predictions.* American Journal of Kidney Diseases, 2010. **55**(4): p. 622-7.

© Copyright: The University of Oxford and Oxford University Hospitals NHS Foundation Trust 2019

57. Horio, M., et al., *Modification of the CKD epidemiology collaboration (CKD-EPI) equation for Japanese: accuracy and use for population estimates.* Am J Kidney Dis, 2010. **56**(1): p. 32-8.

58. Soares, A.A., et al., *Performance of the CKD Epidemiology Collaboration (CKD-EPI) and the Modification of Diet in Renal Disease (MDRD) Study equations in healthy South Brazilians.* Am J Kidney Dis, 2010. **55**(6): p. 1162-3.

59. Hippisley-Cox, J. and C. Coupland, *Predicting the risk of Chronic Kidney Disease in Men and Women in England and Wales: prospective derivation and external validation of the QKidney® Scores.* BMC Family Practice, 2010. **11**(1): p. 49.

60. Watanabe, H., et al., *Close bidirectional relationship between chronic kidney disease and atrial fibrillation: The Niigata preventive medicine study.* American Heart Journal, 2009. **158**(4): p. 629-636.

61. Stevens, L.A., S. Padala, and A.S. Levey, *Advances in glomerular filtration rate-estimating equations.* Curr Opin Nephrol Hypertens, 2010. **19**(3): p. 298-307.

62. Matsushita, K., et al., *Risk implications of the new CKD Epidemiology Collaboration (CKD-EPI) equation compared with the MDRD Study equation for estimated GFR: the Atherosclerosis Risk in Communities (ARIC) Study.* Am J Kidney Dis, 2010. **55**(4): p. 648-59.

63. Becker, B.N. and J.A. Vassalotti, *A software upgrade: CKD testing in 2010.* Am J Kidney Dis, 2010. **55**(1): p. 8-10.

64. Bomback, A.S., A.V. Kshirsagar, and P.J. Klemmer, *Renal aspirin: will all patients with chronic kidney disease one day take spironolactone?* Nat Clin Pract Nephrol, 2009. **5**(2): p. 74-5.

65. Edwards, N.C., et al., *Effect of spironolactone on left ventricular systolic and diastolic function in patients with early stage chronic kidney disease.* Am J Cardiol, 2010. **106**(10): p. 1505-11.

66. Farquharson, C.A. and A.D. Struthers, *Spironolactone increases nitric oxide bioactivity, improves endothelial vasodilator dysfunction, and suppresses vascular angiotensin I/angiotensin II conversion in patients with chronic heart failure.* Circulation, 2000. **101**(6): p. 594-7.

67. Vukusich, A., et al., *A randomized, double-blind, placebo-controlled trial of spironolactone on carotid intima-media thickness in nondiabetic hemodialysis patients.* Clin J Am Soc Nephrol, 2010. **5**(8): p. 1380-7.

68. Pitt, B., *Pharmacotherapy: Cardiovascular effects of aldosterone blockade in CKD.* Nat Rev Cardiol, 2009. **6**(11): p. 679-80.

69. Herzog, C.A., *Kidney disease in cardiology.* Nephrol Dial Transplant, 2011. **26**(1): p. 46-50.

70. Chapman, N., et al., *Effect of spironolactone on blood pressure in subjects with resistant hypertension.* Hypertension, 2007. **49**(4): p. 839-45.

71. Dolan, P., et al., *The time trade-off method: results from a general population study.* Health Econ, 1996. **5**(2): p. 141-54.

72. Dolan, P., *Modeling valuations for EuroQol health states.* Med Care, 1997. **35**(11): p. 1095-108.

© Copyright: The University of Oxford and Oxford University Hospitals NHS Foundation Trust 2019

73. Briggs, A., et al., *Missing... presumed at random: cost-analysis of incomplete data.* Health Econ, 2003. **12**(5): p. 377-92.

74. Latimer, N.R., *NICE DSU Technical support Document 14: Survival analysis for economic evaluations alongside clinical trials-extrapolation with patient level data.*, 2011, School of Health and Related Research, University of Sheffield, UK.

75. Committee, *British National Formulary*. Vol. 63. 2012.

76. Mahmoodi, B.K., et al., *Associations of kidney disease measures with mortality and end-stage renal disease in individuals with and without hypertension: a meta-analysis.* Lancet, 2012. **380**(9854): p. 1649-61.

© Copyright: The University of Oxford and Oxford University Hospitals NHS Foundation Trust 2019

23. APPENDIX A: TRIAL FLOW CHART

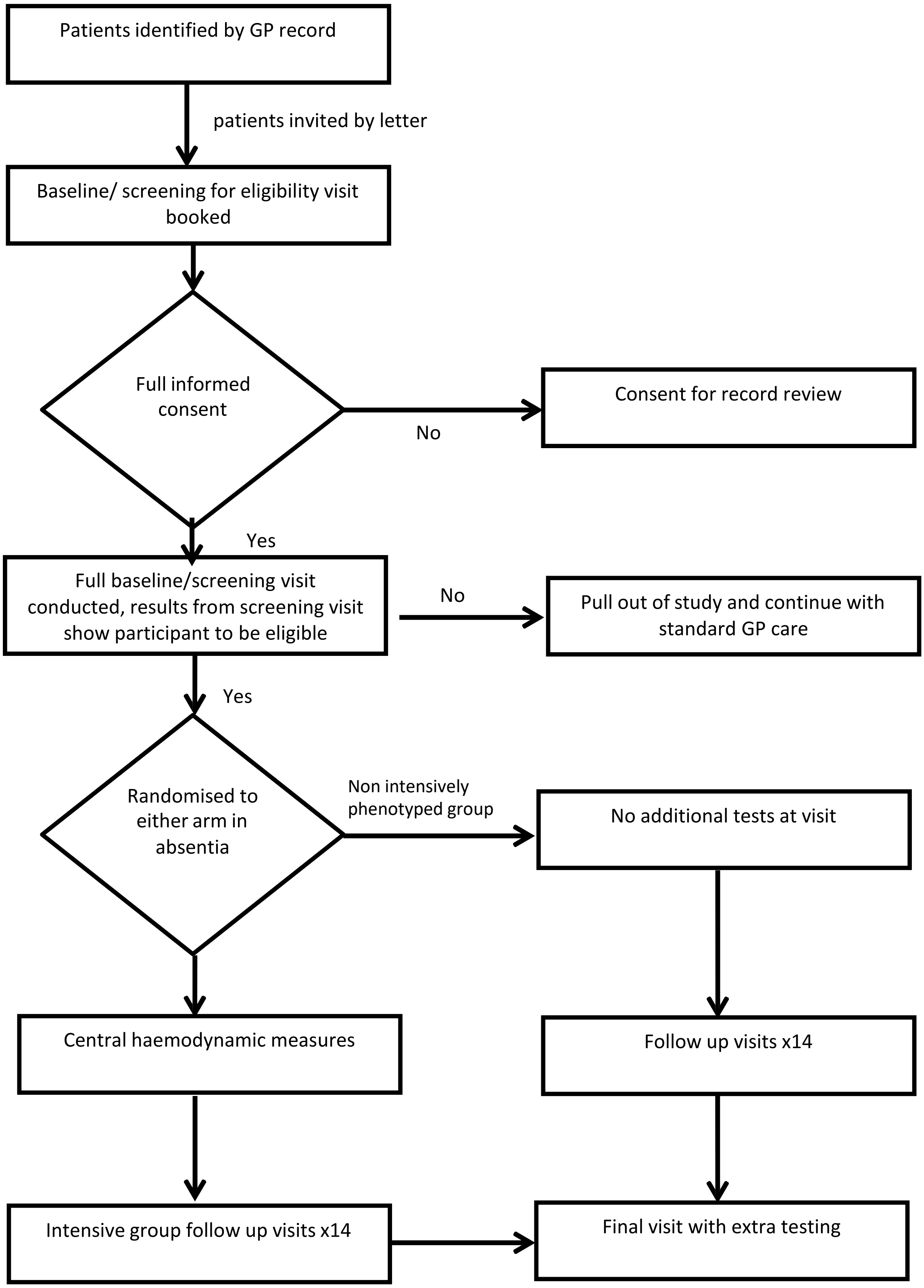

© Copyright: The University of Oxford and Oxford University Hospitals NHS Foundation Trust 2019

24. APPENDIX B: SCHEDULE OF PROCEDURES

		Treatment and Follow-up																
**Week**	**S**	**B**	**0**	**1**	**2**	**4**	**12**	**26**	**39**	**52**	**65**	**78**	**91**	**104**	**117**	**130**	**143**	**156**
**Visit**		**V**		**V1**	**V2**	**V3**	**V4**	**V5**	**V6**	**V7**	**V8**	**V9**	**V10**	**V11**	**V12**	**V13**	**V14**	**V15**
Valid informed consent	Renal profile screening visits where applicable + Informed consent	x	Randomisation *in absentia* and prescription produced once blood results received															
Full demographic details		x																
Medical history		x																x
Clinical history		x																
Concomitant medications		x					x	x		x		x		x		x		x
Weight, Height, Waist/Hip		x																x
Physical examination		x																
Office BP measurement		x		x	x	x	x	x	x	x	x	x	x	x	x	x	x	x
Home BP measurement							x			x		x		x		x		x
KDQOL-SF questionnaire		x						x		x				x				x
QoL EQ-5D-5L questionnaire		x						x		x				x				x
ICECAP-A questionnaire		x						x		x				x				x
QoL VAS		x						x		x				x				X
Diary card (medication monitoring)		x					x	x		x		x		x		x		x
Diary card (Health Economics)		x					x	x	x	x	x	x	x	x	x	x	x	x
Adverse event monitoring		x		x	x	x	x	x	x	x	x	x	x	x	x	x	x	x
Urine ACR		x																x
12 lead ECG		x																x
**Blood Tests for:**																		
Full blood count		x																x
Renal profile		x		x	x	x	x	x	x	x	x	x	x	x	x	x	x	x
Liver function test and bone profile		x						x		x				x				x
Lipids		x						x		x				x				x

© Copyright: The University of Oxford and Oxford University Hospitals NHS Foundation Trust 2019

HbA1c		x						x		x				x				x
Fasting Blood sugar		x						x		x				x				x
BNP (where local labs allow)		x						x		x				x				x
Future analysis (where applicable)		x								x				x				x
**Intensively Phenotyped Group Only**																		
Pulse Wave Velocity		x						x		x				x				x
24h ambulatory BP estimation		x						x		x				x				x

© Copyright: The University of Oxford and Oxford University Hospitals NHS Foundation Trust 2019

25. APPENDIX C: SAE REPORTING FLOW CHART

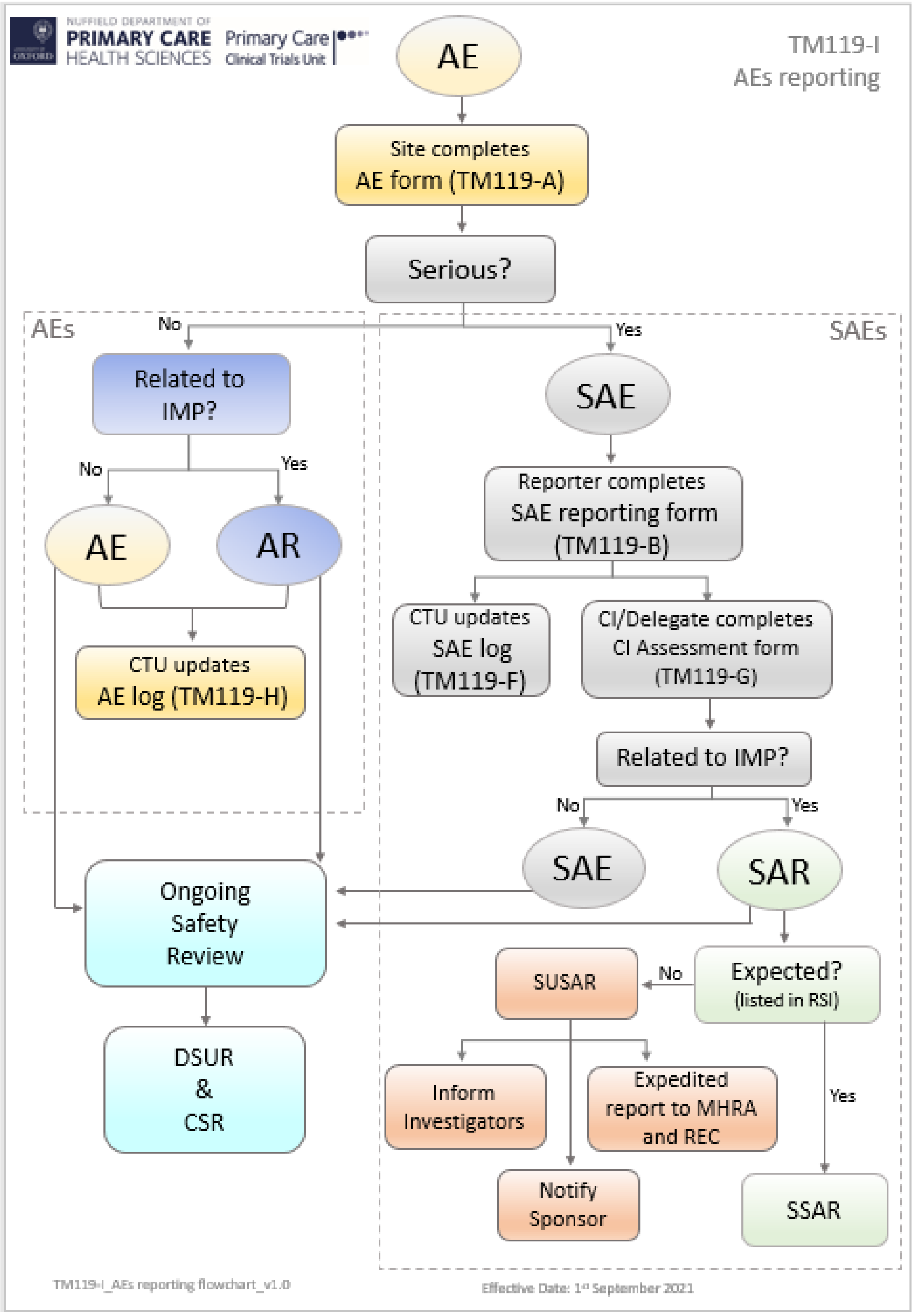

© Copyright: The University of Oxford and Oxford University Hospitals NHS Foundation Trust 2019

26. APPENDIX D: AMENDMENT HISTORY

Amendment No.	Protocol Version No.	Date issued	Author(s) of changes	Details of Changes made
Ethics meeting response	2.0	21st March 2013	Ben Thompson	Removal of gift voucher and addition of travel expenses at request of Ethics Committee and addition of "Lay Title".
Substantial Amendment number 1	3.0	7th March 2013	Ben Thompson	Synopsis – trial phase corrected.<br>Synopsis and section 5.2 – primary endpoint clarified.<br>Section 5.5 and Schedule – Blood sample detail updated.<br>Section 5.6 – Home BP Measurement section updated following further expert clinical input.<br>Section 6.3 – correction of Study Treatment Compliance monitoring detail.<br>Section 8 – Statistics section updated and clarified.<br>Schedule – Concomitant Medications monitoring schedule updated.<br>Throughout – minor changes to correct typos and provide clarification.
Minor Amendment number 1	3.1	19th September 2013	Ben Thompson	Change of funder from NIHR School for Primary Care Research to NIHR HTA (confirmed as minor amendment by Ethics Committee co-ordinator).
Substantial Amendment number 2	4.0	26th November, 2013	Ben Thompson	Addition of sites
Substantial Amendment number 3	4.0	2nd Dec 2013	Ben Thompson	Addition of sites
Substantial Amendment number 4	4.0	12th December 2013	Ben Thompson	Add two questionnaires to measure patient's overall quality of life
Substantial Amendment number 5	4.0	24th January 2013	Ben Thompson	Clarification of process at Derby
Substantial Amendment number 6	4.0	26th Feb 2014	Ben Thompson	Addition of GP and reminder letters
Substantial Amendment number 7	4.0	2nd April 2014	Ben Thompson	Addition of sites
Substantial Amendment number 8	4.0	29th Apr 2014	Ben Thompson	Change of PI Wiltshire

© Copyright: The University of Oxford and Oxford University Hospitals NHS Foundation Trust 2019

Substantial Amendment number 9	5.0	10th June, 2014	Ben Thompson	Alteration of search strategy andeGFR inclusion criterion to improvepatient identification.<br>Introduction of additional screening visit to improve patient identification.<br>Improved patient invitation strategy.<br>Minor clarifications throughout following feedback now trial is recruiting.
Substantial Amendment number 10	5.0	4th Aug 2014	Ben Thompson	Addition of sites
Substantial Amendment number 11	5.0	29th Oct 2014	Ben Thompson	Addition of sites
Substantial Amendment number 12	6.0	27th January, 2015	Ben Thompson	Alteration of inclusion criterion eGFR range to 30 – 50 ml/min/1.73m^2 to encompass larger than anticipated measurement error/fluctuations following initial recruitment.<br>Change in sample size to reflect alteration to eGFR range.<br>Update to causality assessment definitions in “Safety Reporting” section.<br>Minor clarifications throughout.
Substantial Amendment number 13	6.0	26th May 2015	Ben Thompson	Addition of patient facing poster
Substantial Amendment number 14	6.0	21st Jul 2015	Ben Thompson	Addition of sites
Substantial Amendment number 15	6.0	19th Nov 2015	Louise Jones	Change of PI Nottingham
Substantial Amendment number 16	6.0	21st Dec 2015	Louise Jones	Addition of Wales
Substantial Amendment number 17	6.0	18th Apr 2017	Louise Jones	Addition of sites
Substantial Amendment number 18	6.0	8th Aug 2017	Louise Jones	Addition of sites
Substantial Amendment number 19	6.0	21st Nov 2017	Louise Jones	Addition of Northern Ireland
Substantial Amendment number 20	6.0	5th Apr 2018	Louise Jones	Change of PI at BCUHB

© Copyright: The University of Oxford and Oxford University Hospitals NHS Foundation Trust 2019

Substantial Amendment number 21	6.0	2nd May 2018	Louise Jones	Addition of sites
Substantial Amendment number 22	6.0	20th June 2018	Louise Jones	Addition of sites & change of PI at Ash Trees
Substantial Amendment number 23	6.0	1st Aug 2018	Louise Jones	Addition of a CRN, addition of sites and change in PI
Substantial Amendment number 24	7.0	23rd Jul 2020	Louise Jones	Update to statistical section to reflect extension of recruitment phase. Clarification of long term follow up. Minor clarifications throughout.
Substantial Amendment 25	8.0	11th Aug 2022	Joy Rahman	As per details of the letter to Sponsor dated 21/07/2022 and the following: The changes to the protocol are needed to re-add PAD in the summary list of primary outcome components, after its mistaken deletion in an earlier amendment was noted, to correct the inconsistencies in the protocol and regularise this with the other study documents. We have also added analysis of the individual components of the combined primary as a principal secondary outcome. We have also removed left ventricular dysfunction as an outcome in its own right, though it remains if it is part of heart failure outcome. Secondary long-term outcomes have also been removed as it is not certain at the current time if we will be able to address these. In addition, earlier changes to the recruitment strategy prevented our collecting the 24 hour BP data and we can't therefore report on these so they have been removed. Finally, we have made minor word changes to remove minor editing inconsistencies in different sections of the protocol.

Protocol amendments must be submitted to the Sponsor for approval prior to submission to the REC committee, HRA (where required) or MHRA.

© Copyright: The University of Oxford and Oxford University Hospitals NHS Foundation Trust 2019

27. APPENDIX E: TRANSPARENCY STATEMENT FROM THE SPONSOR

The investigators applied a late amendment of the protocol (version 8) prior to study data-lock and analysis to re-add peripheral arterial disease (PAD) back into the synopsis and outcomes tables. PAD had been accidentally deleted in the tables in Protocol version 3 when the outcomes were amended to include independent adjudication of events and new onset events. PAD remained elsewhere in the protocol as documented below. This error was not detected until the adjudication panel met in late 2021. By correcting this earlier error in the synopsis table, the amendment regularised the trial documentation which correctly included PAD in the individual components of the combined primary endpoint in all other sections of the protocol, such as background, prior studies, and the powering of the trial, plus being individually listed in the trial endpoint form from the start, and the detailed statistical analysis plan. This late amendment therefore brings the protocol in line with the study as commissioned by the funders (NIHR HTA Programme). The Sponsor is satisfied that, at the point of this amendment the investigators remained blind to allocation and the numbers of primary endpoints by type.

© Copyright: The University of Oxford and Oxford University Hospitals NHS Foundation Trust 2019

BARACK-D

Statistical Analysis Plan

Benefits of aldosterone receptor antagonism in chronic kidney disease (BARACK D) trial: a prospective randomised open blinded endpoint trial to determine the effect of aldosterone receptor antagonism on mortality and cardiovascular outcomes in patients with stage 3b chronic kidney disease.

Version 2.0 19th December2022

	NAME	TITLE	SIGNATURE	DATE
Written by:	Victoria Harris	Senior Trial Statistician		19/12/2022
Reviewed by:	Nicola Williams	Senior Trial Statistician		20/12/2022
Approved by:	Richard Hobbs	Chief Investigator		19/12/2022

Version History

Version:	Version Date:	Changes:
V0.1	06-Apr-2017	First draft created
V0.14	20-March-2020	Updated following discussion with the trial team
V0.15	17-April-2020	Updated following comments from Meena Patil
V0.16	20-April-2020	Updated with regard to most recent version of the endpoint form.
V0.17	12-Aug-2021	Updated following CI comments
V0.18	10-Sept-2021	Updated following comments from RM
V1.0	20-Sept-2021	Final version

V1.1	12-Jan-2022	Updated derivation of primary outcome.
V1.2	18-Jan-2022	Updated objectives and outcome measures. Add all cause/cardiovascular mortality to secondary analysis of primary outcome.
V1.3	19-Jan-2022	Removed references to intensively phenotyped group, as no data was collected.
V1.4	15-June-2022	Added note about inclusion of Peripheral arterial disease
V1.5	01-November-2022	Added Peripheral arterial disease in line with updated protocol
V1.6	02-December-2022	Updated secondary outcomes.
V1.7	16-December-2022	Updated primary endpoint
V2.0	19-December-2022	Final version

TABLE OF CONTENTS

Table of Contents ... 3
1 Introduction ... 5
1.1 Preface ... 5
1.2 Purpose and scope of the plan ... 5
1.3 Trial overview ... 6
1.4 Objectives ... 7
2 Trial design ... 10
2.1 Visit Structure ... 10
2.2 Outcomes measures ... 11
2.2.1 Primary outcome ... 11
2.2.2 Primary Long-Term outcomes ... 11
2.2.3 Secondary outcomes ... 12
2.2.3.1 To determine the effect of ARA in patients on measures of cardiovascular haemodynamics ... 12
2.2.3.2 To determine the effect of ARA in patients on decline in renal function ... 12
2.2.3.3 To determine the treatment costs and benefits. ... 13
2.2.3.4 To determine the safety of ARA in patients with stage 3b CKD ... 13
2.2.4 Secondary outcomes- Intensively phenotyped subgroup ... 13
2.2.5 Safety outcomes ... 13
2.3 Target population ... 13
2.3.1 Inclusion Criteria ... 14
2.3.2 Exclusion Criteria ... 14
2.4 Sample size ... 15
2.5 Randomisation and blinding in the analysis stage ... 15
3 Analysis – General considerations ... 16
3.1 Descriptive statistics ... 16
3.2 Characteristics of participants ... 16
3.3 FLOW OF PARTICIPANTS THROUGH THE TRIAL ... 18
3.4 Definition of population for analysis ... 18
3.5 Pooling of investigational sites ... 18
3.6 Data Monitoring Committee ... 18
4 PRIMARY ANALYSIS ... 19
4.1 Primary outcome ... 19
4.1.1 Definition of the primary outcome ... 20
4.2 Secondary outcomes – all patients ... 22
4.2.1 Population for Analysis ... 23
4.2.2 The effect of ARA in patients on measures of cardiovascular haemodynamics ... 23
4.2.3 The effect of ARA in patients on decline in renal function ... 24
4.2.4 Treatment costs and benefits ... 24
4.3 Covariate adjustment and population ... 25
4.4 Handling missing data ... 25
4.5 Handling outliers ... 26
4.6 Handling multi-centre/clustered data ... 26
4.7 Multiple comparisons and multiplicity ... 26
4.8 Model assumptions ... 26
5 SECONDARY ANALYSIS ... 27
5.1 Primary outcome ... 27
6 SENSITIVITY ANALYSIS ... 28
6.1 Covariate adjustment ... 28
7 SUBGROUP ANALYSES ... 29
8 SAFETY ANALYSIS ... 30
8.1 All adverse events ... 32

8.2 AEs leading to withdrawal 33
8.3 Serious adverse events 33
8.4 Definitions 33
8.4.1 Adverse Event (AE) 33
8.4.2 Adverse Reaction (AR) 34
8.4.3 Serious Adverse Event (SAE) 34
8.4.4 Serious Adverse Reaction (SAR) 34
8.4.5 Suspected Unexpected Serious Adverse Reaction (SUSAR) 35
8.4.6 Causality and Expectedness 35
8.5 Procedures for Recording Adverse Events 35
9 VALIDATION 36
10 CHANGES TO THE PROTOCOL OR PREVIOUS VERSIONS OF SAP 36
11 Differences from the Protocol 36
12 References 36
13 Appendices 38
Appendix I. Abbreviations 38
Appendix II. Schedule of procedures 39
Appendix III. Outcome assessment schedule 40
Appendix IV. Flow diagram of trial participants 41

1 INTRODUCTION

1.1 PREFACE

The individuals involved in the "Benefits of Aldosterone Receptor Antagonism in Chronic Kidney Disease (BARACK D)" trial are;

Trial Statistician: Ly-Mee Yu

Chief investigator: Richard Hobbs

Senior Trial Manager: Joy Rahman

Data Manager: Meena Patil

The Statistical Analysis Plan was written based on version 8.0 of the protocol dated 11th Aug 2022.

1.2 PURPOSE AND SCOPE OF THE PLAN

This document details the proposed analysis of the primary objective and some secondary objectives for the study "Benefits of Aldosterone Receptor Antagonism in Chronic Kidney Disease (BARACK D)".

The statistical analysis plan will be available on request when the principal papers are submitted for publication in a journal. Suggestions for subsequent analyses by journal editors or referees, will be considered carefully, and carried out as far as possible in line with the principles of this analysis strategy; if reported, the source of the suggestion will be acknowledged. The health economic analysis and analysis of the pilot study are not covered here.

It is not intended that the strategy set out in the plan should prohibit sensible practices. However, the procedures established in the plan will be followed as closely as possible when analysing and reporting the trial. Any modifications and the reasons for those will be fully noted.

Any analysis-related decisions may need to be made based on the pilot study data and observed data from the main trial, such as the distribution of the follow-up data. These decisions will be made prior to the proposed statistical analyses. Any post-hoc exploratory analyses completed to support planned analyses which were not stated in this SAP will be documented and reported in the appendices to the clinical study report. Some flexibility in the desire for pre-planning is anticipated because, for example, further analyses will be expected to be undertaken on the basis of the results of planned analyses.

The plan draws on statistical guidance ICH Harmonised Tripartite Guideline: Statistical Principles for Clinical Trials, and PSI Guidelines for Standard Operating Procedures for Good Statistical Practice in Clinical Research, the CONSORT statement for reporting trials and PC-CTU statistical SOPs.

Any deviations from the statistical analysis plan will be described and justified in the final report of the trial.

The analysis to address the objective looking at treatment costs and benefits is outside the scope of this statistical analysis plan. Analysis to address the costs and benefits will be detailed in a separate health economic analysis plan.

1.3 Trial overview

Chronic kidney disease (CKD) is a major cause of increased mortality and morbidity through increased vascular events and progression to end stage renal failure (ESRF) [1]. These increased events result in CKD having high cost to healthcare systems, with the dialysis required in ESRF benchmarked as at the maximum acceptable cost effectiveness threshold for an intervention by most healthcare systems. However, the most important component of CKD in terms of mortality and morbidity is cardiovascular disease (CVD) [2]. There is a graded inverse relationship between cardiovascular risk and estimated glomerular filtration rate (eGFR), independent of age, sex and other risk factors [3-6] or for creatinine [7]. While the cardiovascular risk of end-stage CKD is extreme, in public health terms the burden resides in early stage (CKD stages 1-3) disease, which is more prevalent, affecting around 40% of those over 70 years. When added to conventional risk factors, renal markers substantially improve risk stratification and CKD is therefore an important and under-recognised risk factor for CVD in the general population [8]. Although the risks of myocardial infarction and other manifestations of coronary artery disease are increased in CKD, the pattern of CVD is atypical, with a much greater incidence of heart failure and sudden cardiac death than in the general CVD population [9-11].

Currently, few therapies have proved effective in modifying the increased CVD risk or the rate of renal decline in CKD. There are accumulating data that aldosterone receptor antagonists (ARAs) may offer cardio-protection and delay renal impairment in patients with the CV phenotype in CKD. The use of ARA in CKD has therefore been increasingly advocated and even termed the 'renal aspirin' [12]. To date, however, no large study of ARAs with renal or CVD outcomes is underway.

This trial evaluates the benefits of an ARA, spironolactone, in patients with stage 3b CKD.

1.4 OBJECTIVES

	Objectives	Outcome Measures	Timepoint(s)
Primary	To determine the effect of aldosterone receptor antagonism on mortality and cardiovascular outcomes (onset or progression of cardiovascular disease) in patients with stage 3b CKD.	Time from randomisation until the first occurring of:<br>• Death or<br>• Hospitalisation for heart disease (coronary heart disease, arrhythmia, atrial fibrillation, sudden death, resuscitated sudden death), stroke, transient ischaemic attack, peripheral arterial disease or heart failure or<br>• First onset of any condition listed above not present at baseline.<br>Primary endpoints will be adjudicated by an independent endpoints committee blinded to treatment arm.	Time from randomisation to first occurrence
Primary Long term	To determine the effect of aldosterone receptor antagonism (even short-term use) on long term mortality and cardiovascular outcome in patients with stage 3b CKD.	Annual rates of:<br>• Death,<br>• Hospitalisation for heart disease (coronary heart disease, arrhythmia, atrial fibrillation, sudden death, resuscitated sudden death), stroke, transient ischaemic attack, peripheral arterial disease or heart failure.<br>• First onset of any condition listed above not present at baseline.	Annual rates, collected via medical notes review

Secondary	To determine the effect of adding an aldosterone receptor antagonism in patients on 1-5 below:<br><br>1. The individual components of the composite primary outcome	• Hospitalisation or new onset heart disease (coronary heart disease, arrhythmia, atrial fibrillation, suddendeath, resuscitated sudden death), stroke, transient ischaemic attack, peripheral arterial disease or heart failure.	• Total occurrences
	2. Measures of cardiovascular haemodynamics	• Change in blood pressure annually and at final visit.	• Annually and at final visit.
	3. Measures of renal function	• Changes in NP<br>• Change in ACR<br>• Changes in eGFR	• Change from baseline, annually and to final visit for NP, ACR and eGFR
	4. Healthcare cost evaluation	• Change in health status on EQ-5D-5L, KDQol, (ICECAP-A and Qol VAS – Oxford only) and NHS resource use (records).	• Change from baseline, annually and to final visit
	5. Safety	• Rates of hypotension (<100mmHg systolic or >20 mmHg systolic drop on standing)<br>• Rates of adverse events<br>• Rates of hyperkalaemia	• Total occurrences<br><br>• Total occurrences

**Secondary Objectives**	**Secondary Endpoints**	**Timepoint(s) for assessment of this outcome measure (if applicable)**
To determine the effect of adding an aldosterone receptor antagonism in patients on 1-5 below:<br><br>1. The individual components of the composite primary outcome	• Hospitalisation or new onset heart disease (coronary heart disease, arrhythmia, atrial fibrillation, suddendeath, resuscitated sudden death), stroke, transient ischaemic attack, peripheral arterial disease or heart failure.	• Total occurrences
2. Measures of cardiovascular haemodynamics	• Change in blood pressure annually and at final visit.	• Annually and at finalvisit
3. Measures of renal function	• Changes in NP<br>• Change in ACR<br>• Changes in eGFR	• Change from baseline, annually and to final visit for NP, ACR and eGFR
4. Healthcare cost evaluation	• Change in health status on EQ-5D-5L, KDQol, (ICECAP-A and Qol VAS – Oxford only) and NHS resource use (records).	• Change from baseline, annually and to final visit
5. Safety	• Rates of hypotension (<100mmHg systolic or >20 mmHg systolic drop on standing)<br>• Rates of adverse events<br>• Rates of hyperkalaemia	• Total occurrences<br>• Total occurrences

2 TRIAL DESIGN

BARACK D is a prospective randomised open blinded endpoint (PROBE) trial where neither the patients, GPs nor statisticians are blinded to the trial treatment. However, the primary endpoints will be assessed by an independent endpoint committee who are blinded to the treatment arm.

Eligible patients, from a minimum of 120 practices recruited by 6 NIHR School for Primary Care Research departments, with previously recorded blood test results suggesting CKD stage 3b will be invited to take part in the study and randomised between (i) ARA spironolactone 25mg OD on top of routine care and (ii) routine care.

Blood pressure in both groups will be titrated (monitored and adjusted accordingly) by the physicians against NICE guideline standards and routine checks of electrolytes undertaken. Primary outcome will be time to changes in cardiovascular events.

Appendix II displays the time schedule of trial procedures.

2.1 VISIT STRUCTURE

Potentially eligible patients will be invited by their practice to attend a baseline assessment and eligibility visit where the trial will be explained. Informed consent will be obtained and baseline assessments performed. Subsequent assessment will continue for both treatment arms for a further 36 months with follow up visits at weeks 1, 2, 4, 12, 26, and then every 13 weeks to 156 weeks. Windows either side of the visits will be two days for V1 and V2, 4 days at V3 and V4, 7 days for V5 and two weeks thereafter (all calculated from date of randomisation).

Patients will also be supplied with a validated home blood pressure monitoring machine along with an additional diary card, to record their self-assessed blood pressure for one week every 6 months. They will take two readings tice daily (e.g. 2 each morning and 2 each evening) over the week.

2.2 OUTCOMES MEASURES

The outcomes assessment schedule is shown in Appendix III.

2.2.1 PRIMARY OUTCOME

Time from randomisation until the first occurring of death or hospitalisation for heart disease (coronary heart disease, arrhythmia, atrial fibrillation, sudden death, resuscitated sudden death), stroke, transient ischaemic attack, peripheral arterial disease or heart failure or first onset of any condition listed above not present at baseline. Primary endpoints will be adjudicated by an independent endpoints committee blinded to treatment arm.

2.2.2 PRIMARY LONG-TERM OUTCOMES

Annual rates collected via medical notes review of death, hospitalisation for heart disease (coronary heart disease, arrhythmia, atrial fibrillation, suddendeath, resuscitated sudden death), stroke, transient ischaemic attack, peripheral arterial disease or heart failure. First onset of any condition listed above not present at baseline.

The following variables will be used to record the occurrence of an event HOSPYN (hospitalised), FSONSETCVD (first onset of cardiovascular disease) and DEATHYN (death). In addition the endpoint will be adjudicated by the endpoint committee (DECISION). If the event is not judged to be an endpoint according to the protocol OR it is not the first onset of hospitalisation for CVD, or death OR there is insufficient information to determine it is an endpoint according to the protocol OR the endpoint committee determines it does not constitute as an endpoint for any other reason then the external adjudication will be coded "No". The reason that the event does not meet the endpoint condition will be captured in the variables REASONIFNO and OTHREASONIFNO. If any of the previously mentioned variables are coded "Yes" and DECISION is coded "Yes" then a primary outcome event will be recorded as having occurred.

If more than one primary outcome event has occurred, time to event will be computed using the date of the earliest occurring event minus RANDDAT (date of randomisation).

If a participant does not experience a primary outcome event they will be censored either at the date of last follow-up, the date of last contact or at the date of withdrawal from the study. Based on the intention to treat (ITT) principle, participants who withdraw from treatment but consent to further follow-up will be censored at the date of last contact rather than the date of withdrawal from treatment.

2.2.3 SECONDARY OUTCOMES

2.2.3.1 TO DETERMINE THE EFFECT OF ARA IN PATIENTS ON MEASURES OF CARDIOVASCULAR HAEMODYNAMICS

Endpoints:

(a) Change from baseline in office recorded systolic blood pressure annually (visit 7, 11) and at final visit. (variables SYSBPR and SYSBPL). Change in systolic BP is collected for right arm and left arm separately. Means of all measurements of BP (in particular measurements taken from different arms) will be calculated at each time point in accordance with NICE guidelines and these mean BP measurements will be used in the analysis. These variables are collected at each visit (see schedule of assessments in appendix III).

Rates of hypotension (<100mmHg systolic recorded in both arms or postural hypotension recorded) at any point during the study. The rates of hypotension across the study period, defined as <100mmHg systolic or >20mmHg systolic drop on standing. The outcome will be hypotension (a binary coded variable defined as hypotension at any point during the study period, coded 1, or no hypotension, coded 0).

(b) This can be reported as two different outcomes:

i. Hypotension (with or without symptoms) when systolic BP is <100mmHg. Specifically this variable is coded "yes" if systolic blood pressure in both arms drops below 100mmHg at any time point and "no" otherwise, i.e. {HYPO_BP=yes IF (SYSBPR AND SYSBPL)<100mmHg} {HYPO_BP=no IF (SYSBPR OR SYSBPL)≥100mmHg}. If this variable is coded yes at any follow up visit, then the outcome will be coded "yes".

ii. Postural / Orthostatic hypotension –postural or symptomatic hypotension leading to study discontinuation. If it is unclear whether the participant has symptoms it will be assumed that the participant has postural hypotension. This information regarding postural drop is recorded in the discontinuation CRF under EWDETAIL as free text and in the adverse event CRF. If postural hypotension is recorded at any time point this outcome will be coded "yes"

The variable HYPOTENSION will be derived as follows:

HYPOTENSION=1 if {(SYSBPR AND SYSBPL) < 100mmHg at ANY Visit} OR {EWDETAIL="postural/symptomatic hypotension" at study discontinuation}

HYPOTENSION=0 if {(SYSBPR OR SYSBPL)>100mmHg at ALL Visits} AND {EWDETAIL postural/symptomatic hypotension"}

2.2.3.2 TO DETERMINE THE EFFECT OF ARA IN PATIENTS ON DECLINE IN RENAL FUNCTION

Endpoint:

Change in Albumin Creatinine Ratio (ACR) (variable ALBCREAT). The percentage change in albumin creatinine ratio from baseline will be computed at 3 years. An increase in ACR level indicates worsening of renal disease. Numbers and percentages with a ≥30% increase in creatinine will be reported.

Change in Estimated Glomerular Filtration Rate (eGFR) (variable GFR). The percentage change from baseline in eGFR from baseline will be computed at 6 months, 1, 2 and 3 years. A reduction in eGFR indicates worsening of renal disease. Numbers and percentages with a drop of ≥25% in eGFR from baseline and ≥20% drop in eGFR from previously reported will also be presented.

2.2.3.3 To determine the treatment costs and benefits.

This objective will form part of the health economic analysis and will be described in a separate health economic analysis plan.

2.2.3.4 To determine the safety of ARA in patients with stage 3b CKD

Endpoints:

(a) Rates of adverse events. All adverse events are recorded in the AE report CRF. The number of participants experiencing at least one AE and the number of AEs per participant will be reported. Number of SAEs will also be reported. Rates of hyperkalaemia:

(b) Hyperkalaemia is defined as a potassium level ≥5.5mmol/l. Potassium [K] is recorded at each visit. A binary variable will be derived as experiencing hyperkalaemia at any time point. Hyperkalaemia will be recorded as "yes" if the participant has potassium levels ≥5.5mmol/l at any point during the study and "no" if at least one potassium measure is recorded and it does not meet the definition of hyperkalaemia. If a participant has hyperkalaemia at any point during the study, the maximum value of Hyperkalaemia for that patients will be used to derive a second variable to indicate whether the maximum hyperkalaemia was mild (5.5-5.9mmol/l), moderate (6.0-6.4mmol/l) or severe (>6.5mmol/l).

2.2.4 Secondary outcomes- Intensively phenotyped subgroup

No patients were recruited to the intensively phenotyped group therefore objective 7 cannot be addressed.

2.2.5 Safety outcomes

All adverse events during the first 6 months of follow-up including rates of hyperkalaemia will be recorded for the analysis of the safety profile of spironolactone in CKD. Following this initial 6 month period, only the following AEs will be monitored by the member of the Research Team performing that visit. Safety concerns (i.e. significant decrease in eGFR (20% between visits, or 25% from baseline), rise in potassium levels (to above 5.5), and drop in BP (to below 100 in both arms)).

2.3 Target population

Eligible patients, from 120 practices recruited by 6 NIHR School for Primary Care Research departments, with previously recorded blood test results suggesting CKD stage 3b will be invited to take part in the trial.

2.3.1 Inclusion Criteria

Participants must fulfil either of the **Search 1** or **Search 2** criteria and all of the following:

Search 1

Evidence of stage 3b CKD using the MDRD equation. This includes patients on the CKD register undergoing annual monitoring who have had two or more recent blood samples in the 30-50 ml/min/1.73m^2 range in the preceding 24 months, with a minimum of 6 weeks between tests.

- Where only one test has been performed in the preceding 24 months and is in the 3b range, the patient will be invited to attend the baseline visit at least 6 weeks from the initial test, the eGFR result from this can be taken as the second confirmatory test. Physicians will also be reminded that standard care suggests a second confirmatory test.

Search 2

- Patients with eGFR results in the preceding 24 months with a reading of25-29 ml/min/1.73m^2
- Participant is willing and able to give informed consent for participation in the study.
- Male or Female, aged 18 years or above.
- Able (in the recruiting physician's opinion) and willing to comply with all study requirements.
- Willing to allow his or her General Practitioner and consultant, if appropriate, to be notified of participation in the study.
- Willing to provide contact details to the Research Team (encompassing recruitment centre and practice staff), for use at any time should the need arise, on trial related matters.
- If the participant is a female of child-bearing potential, they are willing to ensure effective contraception during the trial period.

2.3.2 Exclusion Criteria

The participant may not enter the study if ANY of the following apply:

- Female participants who is pregnant, lactating or planning pregnancy during the course of the study.
- Type 1 diabetes mellitus
- Terminal disease or felt otherwise unsuitable by their physician.
- Chronic heart failure clinical diagnosis or known LVSD with EF<40%.
- Recent myocardial infarction (within 6 months).
- Active cancer with less than 1 year life expectancy or in palliative care.
- Alcohol or drug abuse.
    - Suspected or known current hazardous or harmful drinking, as defined by an alcohol intake of greater than 42 units every week.
    - Suspected or known current substance misuse.
- Most recent potassium result >5.5 mmol/L, where not thought to be spurious, or previous raised potassium needing a reduced dose of ACEI/ARB or intolerance to spironolactone.
- eGFR >60 ml/min/1.73m^2 in the last 6 months and no identifiable reason for a temporary reduction in eGFR.

- Serum potassium at baseline over 5 mmol/L.
- Documented Addisonian crisis and/or on fludrocortisone.
- Documented symptomatic hypotension or baseline systolic blood pressure under 100mmHg.
- Recent acute kidney injury or admission for renal failure.
- ACR > 70 mg/mmol.
- Prescription of medications with known harmful interactions with spironolactone as documented in the British National Formulary including tacrolimus, lithium and cyclosporine.
- Any other significant disease or disorder which, in the opinion of the recruiting physician, may either put the participants at risk because of participation in the study, or may influence the result of the study, or the participant's ability to participate in the study.

2.4 Sample size

The estimate for the cardiovascular (CV) event rate (defined by hospitalisation for coronary heart disease, heart failure, ischemic stroke and peripheral arterial disease) and total mortality rate in patients with CKD 3b (eGFR 30-44 ml/min/1.73m^2) being 11.29 and 4.76 per 100 person years respectively gives a combined event rate of 16.05 per 100 person years. In those with eGFR in the range 45-50 ml/min/1.73m^2, the event rate is conservatively estimated to be 0.667 times as high (10.7 events per 100 person years) and we assume half the participants will fall in this range giving an overall event rate of 13.4 events per 100 person years. To detect a 20% relative risk reduction in death or cardiovascular events within 3 years in the intervention group as compared with the control group (i.e. hazard ratio=0.8) with a two sided significance of 0.05, 1455 participants per arm are required seeking 90% power and assuming 10% drop out rate per year.

We have decided to power the trial conservatively on a 20% risk reduction since this proposed treatment effect is around half the risk reduction observed in the ARA mild heart failure trial (EMPHASIS). The estimated hazard ratio in the EMPHASIS eplerenone versus placebo mild heart failure trial (only mildly symptomatic patients were included) were 0.63 (CI 0.54-0.74, p<0.001) for the composite endpoint of death from CV causes or hospitalisation for heart failure at the median follow up of 21 months. The conservative upper CI for the treatment effect was 26% reduction. The placebo CV event rate in EMPHASIS trial was similar to observational data on CV events in CKD 3b patients.

2.5 Randomisation and blinding in the analysis stage

Randomisation was carried out using Sortition with block randomisation with randomly varying block size. The randomisation was stratified by practice to ensure balance of the two arms within each practice. .

BARACK D is a PROBE trial where neither the patients nor physicians are blinded to the trial treatment but the primary endpoints will be assessed by an independent endpoint committee who are blinded to the treatment arm. The trial statisticians will not be blinded to the treatment allocation.

3 ANALYSIS – GENERAL CONSIDERATIONS

3.1 DESCRIPTIVE STATISTICS

Clinical and demographic variables measured at baseline will be summarised both overall and by trial arm to assess the comparability of the randomised treatment groups. Baseline comparisons will be descriptive only and no formal significance testing will be carried out on baseline variables. Binary or categorical variables will be summarised using sample size (n), number of missing observations (n missing) and proportions within each group. Continuous variables that are approximately normally distributed will be summarised by n, n missing, mean and standard deviation. Continuous variables that are not normally distributed will be summarised using n, n missing, median, minimum and maximum.

Visit windows will be summarised graphically by creating a histogram using the days from baseline to each visit including lines denoting the window for each visit.

3.2 CHARACTERISTICS OF PARTICIPANTS

The patient population at baseline will be reported by treatment group and overall and characterised in terms of:

- Age (computed from age at base line visit: VISDAT-BRTHDAT)
- The number of participants in each of the age groups according to EudraCT guidelines will be reported. These age groups are (adults (18-64 years), from 65-84 years, 85 years and over). Additionally we will report numbers by additional age groups: 18-54 years, 55-64 years, 65-74 years, 75 to 84 years and 85 years and over.
- Gender (SEX)
- Ethnicity (ETHNIC)
- Past medical history (including type II diabetes and coronary artery disease). Presence of: hypertension (HYPYN), diabetes (DIAYN), if no diabetes impaired fasting glucose and/or impaired glucose tolerance (IMGYN), Ischaemic heart disease (IHDYN), heart failure (HFYN), Atrial fibrillation (AFYN), Cerebrovascular disease (CVDYN), peripheral vascular disease (PVDYN), renal disease (RENYN), childhood urinary tract infection (CUTIYN), adulthood urinary tract infection (AUTIYN), Thyroid disease (THYYN), Anaemia (ANAYN), Osteopenia (OPEYN) and Osteoporosis (OPOYN).
- Current medication (CMTRT)
- Smoking status (SMOKSTAT), categorised as never smoker, current smoker or former smoker.
- Weight (WEIGHT)
- Height (HEIGHT)
- Waist circumference (WSTCIR)
- Hip circumference (HIPCIR)

- Office measurement of diastolic BP right arm (DIABPR)
- Office measurement of systolic BP right arm (SYSBPR)
- Office measurement of diastolic BP left arm (DIABPL)
- Office measurement of systolic BP left arm (SYSBPL)

Laboratory and ECG (normal/abnormal(NCS)/abnormal(CS)) test results

- ACR (ALBCREAT)
- eGFR (GFR)
- Potassium (K)
- Creatinine (CREAT)
- Electrocardiogram (ECGCLSIG), categorised as normal, abnormal but not clinically significant or abnormal and clinically significant.

The following test results are recorded on the baseline CRF (text field) and also in the additional laboratory test results. In the analysis the additional laboratory test result CRF will be used to populate the data.

- BNP (BNP) recorded using pg/mL. Where BNP is recorded as ng/L or pmol/L this will be converted to pg/mL.
- HbA1c (IFCC) [HBA1C] [HBA1C]. HBA1C can be recorded using mmol/mol and mmol/molHb. Units will be reported as mmol/mol. Where both IFCC and DCCT measures are recorded, the IFCC will be solely reported. Where only DCCT has been collected it will be converted to IFCC. IFCC can be calculated from DCCT using the following formula: IFCC= (DCCT-2.15)x10.929.
- Lipid profile (serum total cholesterol, serum HDL cholesterol, serum LDL cholesterol, triglyceride and cholesterol to HDL cholesterol ration measurement) (CHOL, HDL, LDL, TRIG and CHOLHDL)
- Practice ID (from Sortition) will be presented by randomised group and overall.

The following test results are recorded on the baseline CRF (coded as normal/abnormal but not clinically significant/abnormal and clinically significant. Where an abnormal result is recorded the actual value should be recorded as a free text field. The values recorded in the free text will be presented using summary statistics, either mean and standard deviations or median and interquartile range as appropriate.

- Full blood count (FBCCLSIG).
- Liver Function Tests (LIVCLSIG)
- Bone Profile Tests (BONCLSIG)
- Fasting Blood sugar (FBSCLSIG)

3.3 Flow of participants through the trial

Recruitment, randomisation and follow-up of participants will be summarised using a CONSORT flow-diagram. An example is shown in Appendix IV. Reasons for all post randomisation discontinuations will be included.

3.4 Definition of population for analysis

The primary analysis will be carried out on all randomised participants, on an Intention to treat (ITT) basis, assuming non-informative censoring for those withdrawn or lost to follow-up. Based in the ITT principle, participants who are withdrawn from treatment (e.g. for safety reasons) but consent to continue follow-up will be included in the ITT analysis. Participants who are withdrawn or lost to follow-up will be censored at the date of withdrawal or date of last follow-up, respectively. All participants will be analysed in the groups to which they were allocated, regardless of treatment compliance.

The safety population will include all participants who took at least one tablet of the study medication. The diary card records the response to the question: "Please confirm you are taking the spironolactone as prescribed?". If the participant records they are taking the medication at the first post-randomisation visit (week 12) they will be included in the safety population. If they record they are not taking the medication, the study discontinuation form will be reviewed to determine whether they took at least one dose. If there is no information available on use of medication it will be assumed they took at least one dose.

3.5 Pooling of investigational sites

Data will be collected across all GP practices that randomised patients in the study. These data will be analysed together and GP practice will be included as a random effect in the statistical analysis.

3.6 Data Monitoring Committee

The trial will have a Data Monitoring and Ethics Committee (DMEC), who will report to and advise the Trial Steering Committee (TSC) who, in turn, will report to and advise the Trial Management group. Both the DMEC and TSC will have independent chairs and 'stop rule' authority to advise early termination of the trial in the event of safety concerns or futility wither through poor recruitment, lack of events, or lack of any treatment effect ('stop rules' to be defined by DMEC). All committees will convene regularly prior to, during, and following the trial. Together, the responsibilities of the committees are to: (a) safeguard the safety, rights and well-being of the trial participants; (b) systematically monitor the trial data and review any analysis as outlined in the Statistical Analysis Plan or as requested by the TSC; (c) make recommendations to the TSC as to whether the trial is operating as expected or if there are any ethical or safety reasons why the trial should not continue; (d) consider data emerging from other related studies and its potential impact on the trial, if requested by the TSC; (e) pick up any trends, such as increases in un/expected events, and take appropriate action; (f) seek additional advice or information from investigators where required; and (g) act or advise, through the Chairman or other consultant, on incidents occurring between meetings that require rapid assessment.

4 PRIMARY ANALYSIS

4.1 PRIMARY OUTCOME

The primary analyses will be conducted on all randomised participants, applying the principle of intention-to-treat (ITT), as far as is practically possible, given any missing data. Specifically, the participants will be analysed in the groups to which they were allocated regardless of compliance with the allocated treatment. The primary outcome will be analysed using a mixed effect Cox proportional-hazards method, with a fixed effect for randomised group and practice included as a random effect, with unstructured variance covariance matrix. Results will be presented as a hazard ratio with 95% confidence interval and associated two-sided P-value. A p-value of 0.05 will be used to assess statistical significance. Time to event will be summarised by randomised group using medians and interquartile range.

TABLE 1 DUMMY TABLE FOR PRIMARY OUTCOME

	TREATMENT (N=)			CONTROL (N=)			MODEL ESTIMATES			
	AVAILABLE N	NUMBER REACHED ENDPOINT, N(%)	TIME TO EVENT	AVAILABLE N	NUMBER REACHED ENDPOINT, N(%)	TIME TO EVENT	HAZARD RATIO	95% LOWER C.I.	95% UPPER C.I.	P-VALUE
**Primary outcome**			**Median (IQR)**			**Median (IQ)**				

4.1.1 DEFINITION OF THE PRIMARY OUTCOME

If an event (as defined in section 2.2.1) occurs the primary outcome will be defined as time from randomisation (RANDDAT) to time of any of the following: time of first onset of or hospitalisation (HOSPDAT), time of first onset of cardiovascular disease (CVDSTDAT), or time of death (DEATHDATE). If multiple events occur then the primary outcome will be defined using the earliest occurring event. In addition, the adjudication by the endpoint committee will be used to determine if the event constitutes an endpoint as defined in the protocol (DECISION).

For those participants not experiencing a relevant event by the end of the study the primary outcome will be calculated as time from randomisation (RANDDAT) to censoring: either date of withdrawal if the participant has withdrawn completely from the study and further follow-up (LVISDAT) or date of last follow-up (VISDAT) if continuing follow-up. For participants who are withdrawn from treatment but consent to further follow-up, censoring will be based on date of last follow-up rather than date of withdrawal based on the ITT principle. Some participants may have multiple withdrawal dates, for example if a participant was withdrawn from medication for safety reasons but consented to further follow-up and then withdrew consent for follow-up at a later date. In these cases the date of final follow-up should be used as the date of censoring. A binary variable will code whether an event as defined in section 2.2.1 has occurred or the participant has been censored.

Two variables will be derived, PEVENT = 1, if the participant experiences a primary outcome event and 0 otherwise, DATEPEVENT = first occurring date of primary outcome event. Derived from:

PEVENT=1 if {{HOSPYN=Yes OR FSONSETCVDYN=Yes OR DEATHYN=Yes} AND {DECISION=Yes}

PEVENT=0 if {HOSPYN=No AND FSONSETCVD=No AND DEATHYN=No} OR {ANY OF HOSPYN, FSONSETCVD, DEATHYN =Missing AND ALL NON MISSING HOSPYN, FSONSETCVD, DEATHYN =No} OR { ANY OF HOSPYN, FSONSETCVD, DEATHYN =Yes AND DECISION=No AND ALL NON MISSING HOSPYN, FSONSETCVD, DEATHYN =No}

IF PEVENT=1 THEN DATEPEVENT=Date first occurance of ANY of HOSPYN, FSONSETCVD, DEATHYN, HDYN, STROKEYN, PADYN, HFYN

IF PEVENT=0 THEN DATEPEVENT={Date of last follow up OR date of last contact OR date of withdrawal IF FOLLOWUP=2}

4.2 SECONDARY OUTCOMES – ALL PATIENTS

Table 4 shows an example of how the secondary outcomes should be presented and table 5 shows an example of the reporting of the results of the secondary analysis models. Models for the secondary analysis are described in more detail in the relevant subsections. For all models the main visits for comparison will are defined in section 2.2.2, see appendix III for the schedule of assessments.

TABLE 2 DUMMY TABLE FOR THE SECONDARY OUTCOMES

	TREATMENT (N=)		CONTROL (N=)		OVERALL	MODEL ESTIMATES			
	AVAILABLE N	SUMMARY	AVAILABLE N	SUMMARY	SUMMARY	EFFECT ESTIMATE[1]	95% LOWER C.I.	95% UPPER C.I.	P-VALUE
**Outcome:**		**Mean (SD)**		**Mean (SD)**	**Mean (SD)**				
Baseline									
6 months									
1 year									
2 year									
3 year									

1. Relative risk for binary outcomes and adjusted mean difference for continuous outcomes.

For continuous variables this is estimated from the linear mixed effects model, adjusted for baseline measures of the outcome and with random effects for participant and GP practice, and for binary variables this is estimated from a log-binomial regression model, adjusted for GP practice.

4.2.1 Population for Analysis

Secondary analysis will also address the secondary endpoints in the ITT population as stated in the protocol. Although the main time points for comparisons are as specified for each outcome, all time points at which an outcome is measured will be incorporated in the model as this will aid with the estimation of treatment effects in the presence of missing data.

4.2.2 The effect of ARA in patients on measures of cardiovascular haemodynamics

Office measurements of systolic blood pressure (SYSBPR) at baseline, 6 months, 1 year, 2 years and 3 years will be summarised across treatment groups using means and standard deviations. Rates of hypotension (defined in section 2.2.2.1) across the whole study period will be summarised across treatment groups using numbers and proportions of those randomised to each arm. The rates of hypotension across the study period will be analysed by log-binomial regression models and predictors will be treatment allocation and GP practice. The treatment effect will be represented as a relative risk with 95% confidence interval and associated P-value.

The mean change in systolic blood pressure each year from baseline will be analysed with a linear mixed effects model. The model will include systolic blood pressure measurement at each time point and will adjust for baseline measurement and clustering within GP practice will be accounted for as a random effect within the model. An interaction between time and randomised group will be fitted to allow estimation of treatment effect at 6 months and 1, 2 and 3 years. Although 6 months and 1, 2 and 3 years are the main time points of interest, all visits at which BP is recorded will be included in the model to aid estimation of treatment effects in the presence of missing data. The model will also include a patient specific random intercept nested within a practice specific random intercept. Results will be presented as mean difference in change from baseline in systolic blood pressure between the randomised groups at 6 months and 1, 2 and 3 years, with 95% confidence interval and associated 2-sided p value.

The model will include the following fixed effects:

- Time
- Treatment allocation
- Time×treatment allocation
- Baseline systolic blood pressure

The model will include the following random effects:

- GP practice
- Participant ID

The distribution of the change from baseline will be formally assessed for evidence of departure from normality. If necessary, data will either be transformed or analysed using a non-parametric equivalent.

4.2.3 The effect of ARA in patients on decline in renal function

The decline in renal function will be measured by eGFR and ACR.

eGFR (GFR) at baseline, 6 months and annually will be summarised across treatment arms using means and standard deviations. Mean change in eGFR from baseline to each visit will be assessed using linear mixed models in the same way as for blood pressure.
The model will include the following fixed effects:

- Time
- Treatment allocation
- Time×treatment allocation
- Baseline eGFR

The model will include the following random effects:

- GP practice
- Participant ID

Results will be presented as mean difference in change from baseline in eGFR between the randomised groups at each visit, with 95% confidence interval and associated 2-sided p value.

ACR (ALBCREAT) at baseline and the final visit will be summarised across treatment arm using means and standard deviations. The change in ACR from randomisation to the final visit (visit 15, 3 years) will be assessed using a linear mixed effects model. The model will include baseline ACR and treatment allocation as fixed predictors. Random effects will be participants nested within GP practices. Results will be presented as the difference in mean change in ACR, with a 95% confidence interval and corresponding P-value.

The distribution of the change from baseline will be formally assessed for evidence of departure from normality. If necessary, data will either be transformed or analysed using a non-parametric equivalent.

4.2.4 Treatment costs and benefits

A health economic analysis will be integrated into the trial. Details of the health economic analysis will be detailed in a separate analysis plan. Mixed effects models will be used to analyse the health economic measures in the same way as the clinical measures.

4.3 COVARIATE ADJUSTMENT AND POPULATION

All secondary analyses will account for GP practice as per the primary outcome. These analyses will be carried out on the ITT population.

4.4 HANDLING MISSING DATA

Data will be analysed using an intention to treat analysis. All randomised patients will be included in the primary analysis up to the last follow-up or primary outcome event as appropriate, assuming non-informative censoring for those withdrawn from the study and not consenting to continued follow-up or those lost to follow-up.

All baseline covariates are expected to be observed. Baseline values will be summarised for those who did and did not complete follow up measurements, separately for those who withdrew completely from the study or were lost to follow-up and those who were withdrawn from medication but consented to further follow-up, in order to assess which observed characteristics are related to missingness. In the unlikely event that there is missing data at baseline then these will be imputed using a suitable method (for example a simple method such as mean imputation if the proportion of missing data is small, i.e. <10%, or the more sophisticated method of multiple imputation if there are substantial missing data at baseline).

For the secondary outcomes mixed models can account for missing data in the outcome at different time points so long as the outcome is observed at least one time point. Although time points for the main comparisons are as specified, all time points at which an outcome is measured will be included in the analysis model in order to aid with estimation in the presence of missing data. This analysis is valid under the missing at random (MAR) assumption, i.e. that the probability of a value being missing depends only on variables included in the model. The missing at random assumption will be tested for each secondary outcome as far as is possible by analysing each baseline covariate in a logistic regression model to determine which if any are associated with missingness (where the outcome is presence/absence of the outcome at the primary endpoint). Should any baseline clinical or demographic variable be predictive of later dropout this variable will be included as an additional covariate in the analysis, in order to ensure the MAR assumption is valid.

4.5 HANDLING OUTLIERS

A possible outlier is defined here as a data-point being at least three standard deviations from the mean of its distribution in the variable at that time-point and in relation to previous readings of that measure if appropriate. Potential bivariate or multivariate outliers, such as those identified through differences between baseline and follow-up values, will be flagged for checking and by plotting correlated measures.

Residual outliers (these are observations with residuals of large magnitude i.e. observation's outcome value is unusual conditional on its explanatory variable values) will also be flagged for checking and by plotting the residuals after fitting the regression models. Cook's Distance, leverage and other measures of influential outliers in linear regression models will be examined.

Analysis will proceed by retaining identified outliers including data-point outliers, so long as these values are considered to be plausible. Sensitivity analysis will involve a robust approach in which outliers are excluded by being set to missing. If there is no material difference in conclusions, the analysis retaining outliers will be solely reported.

4.6 HANDLING MULTI-CENTRE/CLUSTERED DATA

Practice will be adjusted for in the final analysis by being included as a fixed effect in the cox proportional hazards model in the analysis of the primary outcome. For the secondary analysis linear mixed models will include a random effect for practice to account for the clustering of patients within practices.

4.7 MULTIPLE COMPARISONS AND MULTIPLICITY

Adjustments for multiple comparisons and multiplicity are not necessary given that only one pre-defined hypothesis will be tested involving a single test and a single primary outcome. Several secondary outcomes are specified and analysed at multiple time points. These will be considered as exploratory in nature and so no correction for multiple testing will be carried out. All secondary outcomes will be reported in the analysis report and any resulting publications to avoid bias arising from selective reporting.

4.8 MODEL ASSUMPTIONS

A key assumption of the Cox model is that of proportional hazards - the survival curves for the two strata must have hazard functions that are proportional over time (i.e. constant relative hazard). The assumption of proportional hazards for the primary outcome will be examined using the log-log plot of survival (-ln(-ln(survival)) verses ln(time), examination of the Schoenfeld residuals and formally with a proportional hazards test. If the proportional hazards assumption is violated a suitable alternative survival method will be considered, for example time varying covariates may be included in the analysis model.

Similarly, alternative methods will be considered if any violation of assumptions is detected in any of the methods for the secondary outcomes. In particular linear mixed effects models assume normality of the residuals. The distribution of continuous secondary outcomes will be explored graphically, e.g. using a normal qq-plots. If the outcome is considered to be non-normally distributed a data transform will be considered, e.g. a logarithmic transform for positively skewed data, and analysis will instead be carried out on the transformed data. Otherwise bootstrapping may be used to generate standard errors and confidence intervals that are valid under departures from normality in the dataset.

5 SECONDARY ANALYSIS

5.1 PRIMARY OUTCOME

Secondary analysis will consider the individual components of the primary composite outcome in the ITT population, i.e. cardiovascular disease and onset of or hospitalisation, separately, and also all-cause mortality. Time to event will be defined as time from randomisation (RANDDAT) to time of each of the following: time of first onset of or hospitalisation (HOSPDAT), time of first onset of cardiovascular disease (CVDSTDAT), or time of death (DEATHDAT). Additionally time to death from cardiovascular disease will be analysed separately. Death from cardiovascular disease will be coded from the derived from DEATHTYPE (death from cardiovascular disease DEATHTYPE=1).

If a participant experiences more than one endpoint then time to event will be computed from the time of the first occurrence of the relevant event i.e. time to each component will be computed the time to that event even if another component of the primary endpoint occurs earlier). Where relevant competing risks (e.g. mortality, where a participant dies before the end of the study without experiencing another relevant endpoint) will be treated as a form of non-informative censoring, with censoring taken as the time point at which the competing risk occurred. Censoring for those participants who complete the study or withdraw from follow-up will be treated in the same way as for the primary outcome, i.e. censoring will occur at the date of last follow-up for all participants on an ITT basis.

The cox proportional hazards approach will be repeated for individual components of the primary composite endpoint and all-cause mortality as secondary analyses, adjusting for GP practice. Time to event will be summarised using median and range for each endpoint and model estimates will be presented as hazard ratios with associated p-values (see table 7).

TABLE 3 DUMMY TABLE FOR COMPONENTS OF THE PRIMARY OUTCOME

Summaries of data:

	TREATMENT (N%)		CONTROL (N%)		OVERALL
	AVAILABLE N	SUMMARY	AVAILABLE N	SUMMARY	SUMMARY
**Time from randomisation to cardiovascular disease**	N	Median (Range)	N	Median (Range)	Median (Range)
**Treatment effect[1], [95% Confidence interval], p-value**					
**Time from randomisation to hospitalisation**					
**Treatment effect[1], [95% Confidence interval], p-value**					
**Time from randomisation to all-cause mortality**					
**Treatment effect[1], [95% Confidence interval], p-value**					
**Time from randomisation to mortality from cardiovascular disease**					
**Treatment effect[1], [95% Confidence interval], p-value**					

1. Hazard ratio as estimated from the cox-proportional hazards model, adjusted for GP practice.

6 SENSITIVITY ANALYSIS

6.1 COVARIATE ADJUSTMENT

To test the robustness of the result for the primary outcome, a sensitivity analysis will be carried out, using the cox proportional hazards model, adjusting the following pre-specified baseline prognostic factors: diastolic (DIABPR/DIABPL) and/or systolic blood pressure (SYSBPR/SYSBPL) above or below NICE target, type II diabetes (DIAYN) and coronary artery disease (IHDYN) BP targets will be based on the following measures of clinical blood pressure:

- People aged under 80 years: lower than 140/90 mm Hg (systolic/diastolic)
- People aged 80 years or over: lower than 150/90 mm Hg (systolic/diastolic)

As blood pressure is recorded in both arms at each visit, we will consider the average of the two measures as meeting the criteria.

TABLE 4 DUMMY TABLE FOR THE ANALYSIS OF THE PRIMARY OUTCOME

HAZARD RATIO[1]	95% LOWER C.I.	95% UPPER C.I.	P-VALUE

1. Adjusted for the following covariates: systolic or diastolic blood pressure above NICE target, type II diabetes, coronary artery disease and GP practice.

7 SUBGROUP ANALYSES

TABLE 5 DUMMY TABLE FOR THE SUBGROUP ANALYSIS OF THE PRIMARY OUTCOME

VARIABLE	HAZARD RATIO	95% LOWER C.I.	95% UPPER C.I.
**Treatment**			
**Practice:**			
**Subgroup**			
**Subgroup×Treatment**			

All subgroup analyses are exploratory and will be conducted on the primary outcome only and on the ITT population.

The efficacy of the intervention by each subgroup will be examined separately. Subgroups to be tested include:

1. Presence/absence of type II diabetes at baseline
2. Presence/absence of coronary artery disease at baseline
3. Diastolic and/or systolic blood pressure above/below the NICE targets at baseline (Defined as- People aged under 80 years: lower than 140/90 mm Hg (systolic/diastolic), People aged over 80 years: lower than 150/90 mm Hg (systolic/diastolic)

We will include appropriate subgroup by treatment interaction terms and add these terms to the Cox regression model used in the primary outcome analysis.

8 SAFETY ANALYSIS

TABLE 6 DUMMY TABLE FOR ADVERSE EVENT RECORDING

	TREATMENT (N=)	CONTROL (N=)	OVERALL
	SUMMARY	SUMMARY	SUMMARY
Number of adverse events			
Number of participants experiencing an adverse event	N(%)	N(%)	N(%)
Fisher's exact test (P-value)			
Number of serious adverse events			
Number of participants experiencing a serious adverse event	N(%)	N(%)	N(%)
Fisher's exact test (P-value)			
Number experiencing Hyperkalaemia	N(%)	N(%)	N(%)
Fisher's exact test (P-value)			
Maximum potassium level:<br>• <5.5mmol/l<br>• 5.5-5.9mmol/l<br>• 6.0-6.4mmol/l<br>• >6.5mmol/l	N(%)	N(%)	N(%)
Fisher's exact test (P-value)			

Occurrence of adverse events and type of adverse events will be recorded in the variable AETERM. Number of adverse effects will be tabulated according to randomised group assignments and the proportions will be compared using Fisher's exact test. Instances of hyperkalaemia will be tabulated by treatment arm and proportions experiencing hyperkalaemia will be compared using Fisher's exact test.

TABLE 7 TYPE OF ADVERSE EVENTS BY GROUP

	TREATMENT (N=)	CONTROL (N=)	OVERALL (N=)
Number of events			
Hyperkalaemia	N	N	N
Deterioration of renal disease	N	N	N
Hypotension[1]	N	N	N
Other adverse event:<br>- Details	N	N	N
Number of participants			
Hyperkalaemia	N(%)	N(%)	N(%)

	**TREATMENT (N=)**	**CONTROL (N=)**	**OVERALL (N=)**
**Deterioration of renal disease**	**N(%)**	**N(%)**	**N(%)**
**Hypotension**	**N(%)**	**N(%)**	**N(%)**
**Other adverse event:**<br>- **Details**	**N(%)**	**N(%)**	**N(%)**

[1]Defined as <100mmHg systolic or >20 mmHg systolic drop on standing

The safety analysis will focus on the adverse events. The safety population will include all participants who took at least one tablet of the study medication. Safety monitoring will invoke discontinuation rules for hyperkalaemia, deterioration of renal disease and hypotension. These will be recorded in the adverse event report form in the variable AETERM. These will be summarised by treatment arm as shown in table 11.

All adverse events (AEs) will be recorded at trial visits on the adverse event report form for the initial 6 months of follow-up by the member of the Research Team conducting that visit for the previous inter-visit period. Following this initial 6 month period, only the following AEs will be monitored by the member of the Research Team performing that visit in accordance with PC-CTU SOP TM19 “Pharmacovigilance”: enlargement of breasts in men and women; erectile dysfunction; irregular periods; vaginal bleeding after the menopause; deepening of the voice in women, change in the tone of voice in men; excessive hair growth; tiredness; palpitations; and numbness and tingling.

All serious adverse events (SAEs) occurring during the study, either observed by the recruiting GP or reported by the participant, whether or not attributed to study medication, will be recorded on the CRF. The severity of adverse events will be assessed on the following scale: 1=mild; 2=moderate; 3=severe.

8.1 ALL ADVERSE EVENTS

TABLE 8 ADVERSE EVENTS BY TREATMENT ARM

	TREATMENT (N%)				CONTROL (N%)				OVERALL			
**SEVERITY**	NONE	MILD	MODERATE	SEVERE	NONE	MILD	MODERATE	SEVERE	NONE	MILD	MODERATE	SEVERE
**Adverse event, N(%)**												
**Comparison, χ²(df) (P-value)**												

	TREATMENT (N%)				CONTROL (N%)				OVERALL			
**RELATEDNESS TO TREATMENT**	NOT [illegible]	POSSIBLY	PROBABLY	DEFINITELY	NOT [illegible]	POSSIBLY	PROBABLY	DEFINITELY	NOT [illegible]	POSSIBLY	PROBABLY	DEFINITELY
**Adverse event, N(%)**												

PARTICIPANT ID	DESCRIPTION	SYSTEM ORGAN CLASS	DATE OF ONSET	DATE OF RESOLUTION	SEVERITY	RELATEDNESS TO STUDY MEDICATION

Safety will be evaluated by tabulations of percentages, severity and relationship to study drug of individual AEs. Severity of AEs will be compared between the two treatment arms using a chi-squared test or Fisher's exact test if any cell frequency in the cross tabulation is less than 5. SAEs will be tabulated by treatment arm and severity rating as shown in table 10. The variable AESER records whether an adverse event is serious and the variable AESEV records the severity rating. Relatedness of adverse events to treatment will be tabulated across treatment arms (variable AEREL).

All information pertaining to AEs will be listed by participant, narrative statements given by the investigator, system organ class, date of onset, date of resolution, severity, and relationship to the study medication.

8.2 AEs LEADING TO WITHDRAWAL

TABLE 9 TABLE OF ADVERSE EVENTS LEADING TO WITHDRAWAL BY TREATMENT GROUP AND SYSTEM ORGAN CLASS

	TREATMENT (N%)	CONTROL (N%)	OVERALL
SYSTEM ORGAN CLASS:			

TABLE 10 BASELINE CHARACTERISTICS OF PARTICIPANTS WITH AES LEADING TO WITHDRAWAL

PARTICIPANT ID	AGE	GENDER	HEIGHT	WEIGHT	MEDICAL HISTORY	OFFICE BP MEASUREMENT	ABNORMAL MEASUREMENTS	MEDICATION

A summary of percentages of AEs leading to withdrawal, by treatment group, system organ class will be prepared. No statistical tests will be conducted. A data listing of AEs leading to withdrawal will also be provided, displaying details of patient baseline characteristics and the event(s) captured on the CRF.

8.3 SERIOUS ADVERSE EVENTS

TABLE 11 TABLE OF SERIOUS ADVERSE EVENTS BY TREATMENT GROUP AND SYSTEM ORGAN CLASS

	TREATMENT (N%)	CONTROL (N%)	OVERALL
SYSTEM ORGAN CLASS:			

A summary of percentages of SAEs by treatment group, system organ class will be prepared for the safety population. No statistical tests will be conducted. A data listing of SAEs will also be prepared, displaying details of patient baseline characteristics and details of the event(s) captured on the CRF.

8.4 DEFINITIONS

8.4.1 ADVERSE EVENT (AE)

An AE or adverse reaction is any untoward medical occurrence in the participant administered the study medication which does not necessarily have to have a causal relationship with the study medication. An AE can therefore be any unfavourable and unintended sign (including an abnormal laboratory finding), symptom or disease temporally associated with the use of the study medication, whether or not considered related to the study medication.

8.4.2 Adverse Reaction (AR)

An adverse reaction is defined as an untoward and unintended response to the study medication. The phrase "responses to a medicinal product" means that a causal relationship between the study medication and an AE is at least a reasonable possibility, i.e. the relationship cannot be ruled out. Causality of all cases will be judged by the site physician.

8.4.3 Serious Adverse Event (SAE)

A serious adverse event is any untoward medical occurrence that at any dose:

- Results in death,
- Is life-threatening, NOTE: The term "life-threatening" in the definition of "serious" refers to an event in which the participant was at risk of death at the time of the event; it does not refer to an event which hypothetically might have caused death if it were more severe.
- Requires inpatient hospitalisation or prolongation of existing hospitalisation,
- Results in persistent or significant disability/incapacity, or
- Is a congenital anomaly/birth defect.
- Other important medical events. NOTE: Other events that may not result in death, are not life threatening, or do not require hospitalisation, may be considered a serious adverse event when, based upon appropriate medical judgement, the event may jeopardise the patient and may require medical or surgical intervention to prevent one of the outcomes listed above.

To ensure no confusion or misunderstanding of the difference between the terms "serious" and "severe", which are not synonymous, the following note of clarification is provided:

> "The term "severe" is often used to describe the intensity (severity) of a specific event (as in mild, moderate, or severe myocardial infarction); the event itself, however, may be of relatively minor medical significance (such as severe headache). This is not the same as "serious," which is based on patient/event outcome or action criteria usually associated with events that pose a threat to a participant's life or functioning as defined in the bullet points above. Seriousness (not severity) serves as a guide for defining regulatory reporting obligations."

8.4.4 Serious Adverse Reaction (SAR)

An adverse event that is both serious and, in the opinion of the reporting recruiting physician, believed with reasonable probability to be due to one of the study treatments, based on the information provided.

8.4.5 Suspected Unexpected Serious Adverse Reaction (SUSAR)

A serious adverse reaction, the nature or severity of which is not consistent with the applicable product information (e.g. Investigator's Brochure for an unapproved investigational product or summary of product characteristics for an approved product).

8.4.6 Causality and Expectedness

The relationship of each adverse event to the trial medication must be determined by a medically qualified individual according to the following definitions:

Definitely related: the known effects of the IMP, its therapeutic class or based on challenge testing suggest that the IMP is the most likely cause.

Probably related: the temporal relationship and absence of a more likely explanation suggest the event could be related to the IMP.

Possibly related: although a relationship to the IMP cannot be completely ruled out, the natur of the event, the underlying disease, concomitant medication or temporal relationship make other explanations possible.

Unrelated: where an event is not considered to be related to the IMP.

All AEs (SAEs) labelled possibly, probably or definitely will be considered as related to the IMP.

8.5 Procedures for Recording Adverse Events

All site staff are appropriately trained in the procedures to follow and the forms to use by the PC-CTU prior to study initiation. Regular central monitoring for all studies and site monitoring, as determined by the trial specific risk assessment, will be used to ensure that all adverse events are identified and acted on appropriately.

All adverse events will be recorded at trial visits for the initial 6 months of follow-up by the member of the Research Team conducting that visit for the previous inter-visit period. Following this initial 6 month period, only the following AEs will be monitored by the member of the Research Team performing that visit in accordance with PC-CTU SOP TM19 "Pharmacovigilance":

- Enlargement of breasts in men and women
- Erectile dysfunction
- Irregular periods
- Vaginal bleeding after the menopause
- Deepening of the voice in women, change in the tone of voice in men
- Excessive hair growth
- Tiredness
- Palpitations

- Numbness and tingling

AEs considered related to the study medication as judged by a medically qualified member of the Research Team or the Sponsor will be followed until resolution or the event is considered stable, clinically insignificant or asymptomatic. All related AEs that result in a participant's withdrawal from the study or are present at the end of the study, should be followed up until a satisfactory resolution occurs.

It will be left to the recruiting physician's clinical judgment whether or not an AE is of sufficient severity to require the participant's removal from treatment and, if treatment is withdrawn, the reason will be recorded. A participant may also voluntarily withdraw from treatment due to what he or she perceives as an intolerable AE. If either of these occurs, the participant must undergo an end of study assessment and be given appropriate care under medical supervision until symptoms cease or the condition becomes stable.

- The severity of events will be assessed on the following scale: 1 = mild, 2 = moderate, 3 = severe.
- The relationship of AEs to the study medication will be assessed by a medically qualified member of the Research Team.

9 VALIDATION

The primary analysis will be validated by a senior trial statistician or a delegate.

10 CHANGES TO THE PROTOCOL OR PREVIOUS VERSIONS OF SAP

11 DIFFERENCES FROM THE PROTOCOL

Peripheral artery disease was included in the definition of the primary outcome in the sample size calculation in the protocol but not in the description of the outcome. To be consistent with the endpoint form it is included in the definition of the outcome in the SAP.

12 REFERENCES

1. Keith, D.S., et al., Longitudinal follow-up and outcomes among a population with chronic kidney disease in a large managed care organization. Arch Intern Med, 2004. **164**(6): p. 659-63.

2. Go, A.S., et al., Chronic kidney disease and the risks of death, cardiovascular events, and hospitalization. N Engl J Med, 2004. **351**(13): p. 1296-305.

3. Matsushita, K., et al., Association of estimated glomerular filtration rate and albuminuria with all-cause and cardiovascular mortality in general population cohorts: a collaborative meta-analysis. Lancet, 2010. **375**(9731): p. 2073-81.

4. Coresh, J., et al., Prevalence of chronic kidney disease in the United States. JAMA, 2007. **298**(17): p. 2038-47.

5. Abramson, J.L., et al., Chronic kidney disease, anemia, and incident stroke in a middle-aged, community-based population: the ARIC Study. Kidney Int, 2003. **64**(2): p. 610-5.

6. Van Biesen, W., et al., The glomerular filtration rate in an apparently healthy population and its relation with cardiovascular mortality during 10 years. Eur Heart J, 2007. **28**(4): p. 478-83.

7. Shlipak, M.G., et al., Cystatin C and the risk of death and cardiovascular events among elderly persons. N Engl J Med, 2005. **352**(20): p. 2049-60.

8. Tonelli, M., et al., Chronic Kidney Disease and Mortality Risk: A Systematic Review. Journal of the American Society of Nephrology, 2006. 17(7): p. 2034-2047.

9. Foley, R.N., et al., Left ventricular hypertrophy in new hemodialysis patients without symptomatic cardiac disease. Clin J Am Soc Nephrol, 2010. 5(5): p. 805-13.

10. Foley, R.N., et al., Chronic kidney disease and the risk for cardiovascular disease, renal replacement, and death in the United States Medicare population, 1998 to 1999. J Am Soc Nephrol, 2005. 16(2): p. 489-95.

11. Collins, A.J., et al., Chronic kidney disease and cardiovascular disease in the Medicare population. Kidney Int Suppl, 2003(87): p. S24-31.

12. Bomback, A.S., A.V. Kshirsagar, and P.J. Klemmer, Renal aspirin: will all patients with chronic kidney disease one day take spironolactone? Nat Clin Pract Nephrol, 2009. **5**(2): p. 74-5.

13. Kenward, M. G. and J. Carpenter (2007). Multiple imputation: current perspectives. Stat Methods Med Res 16(3): 199-218.

14. Chronic kidney disease: early identification and management of chronic kidney disease in adults in primary and secondary care. NICE clinical guideline 73, 2008.

15. Hypertension: management of hypertension in adults in primary care. NICE clinical guideline 34 (partial update of NICE guideline 18), 2006.

16. Group E. EuroQoL: a new facility for the measurement of health related quality of life. Health Pol. 2001;16:199–208.

17. http://www.euroqol.org/about-eq-5d/valuation-of-eq-5d/eq-5d-5l-value-sets.html

18. Hays, R. D., Amin, N., Leplege, A., Carter, W. B., Mapes, D. L., Kamberg, C., ... & Coons, S. J. (1997). Kidney Disease Quality of Life Short Form (KDQOL-SFtm), Version 1.2: A Manual for Use and Scoring (French Questionnaire, France). *Santa Monica (CA): RAND*.

19. Hays, R. D., Leplege, A., Carter, W. B., Kamberg, C., Kallich, J., & Coons, S. J. (1997). Kidney Disease Quality of Life Short Form (KDQOL—Sth), Version 1.2: A Manual for Use and Scoring (French Questionnaire, France).

13 APPENDICES

APPENDIX I. ABBREVIATIONS

ACR	Albumin creatinine ratio
AE	Adverse event
ARA	Aldosterone receptor antagonist
BMI	Body mass index
BNP	B-type natriuretic peptide
CKD	Chronic kidney disease
CV, CVD	Cardiovascular, cardiovascular disease
eGFR	Estimated glomerular filtration rate
ESRF	End stage renal failure
QoL	Quality of life
SAP	Statistical analysis plan
SAE	Serious adverse event
SOP	Standard operating procedure
TIA	Transient ischaemic attack
VAS	Visual analogue scale

APPENDIX II. SCHEDULE OF PROCEDURES

	Treatment and Follow-up																
**Week**	**B**	**0**	**1**	**2**	**4**	**12**	**26**	**39**	**52**	**65**	**78**	**91**	**104**	**117**	**130**	**143**	**156**
**Visit**	**V**		**V1**	**V2**	**V3**	**V4**	**V5**	**V6**	**V7**	**V8**	**V9**	**V10**	**V11**	**V12**	**V13**	**V14**	**V15**
Valid informed consent	x	**Randomisation *in absentia* and prescription produced once blood results received**															
Full demographic details	x																
Medical history	x																x
Clinical history	x																
Concomitant medications	x					x	x		x		x		x		x		x
Weight, Height, Waist/Hip	x																x
Physical examination	x																
Office BP measurement	x		x	x	x	x	x	x	x	x	x	x	x	x	x	x	x
Home BP measurement						x			x		x		x		x		x
KDQOL-SF questionnaire	x						x		x				x				x
QoL EQ-5D-5L questionnaire	x						x		x				x				x
ICECAP-A questionnaire	x						x		x				x				x
QoL VAS	x						x		x				x				x
Diary card (medication monitoring)	x					x	x		x		x		x		x		x
Diary card (Health Economics)	x					x	x	x	x	x	x	x	x	x	x	x	x
Adverse event monitoring	x		x	x	x	x	x	x	x	x	x	x	x	x	x	x	x
Urine ACR	x																x
12 lead ECG	x																x
**Blood Tests for:**																	
Full blood count	x																x
Renal profile	x		x	x	x	x	x	x	x	x	x	x	x	x	x	x	x
Liver function test and bone profile	x						x		x				x				x
Lipids	x						x		x				x				x
HbA1c	x						x		x				x				x
Fasting Blood sugar	x						x		x				x				x
BNP	x						x		x				x				x
Future analysis (where applicable)	x								x				x				x
**Intensively Phenotyped Group Only**																	
Pulse Wave Velocity	x						x		x				x				x
24h ambulatory BP estimation	x						x		x				x				x

APPENDIX III. OUTCOME ASSESSMENT SCHEDULE

	V*	V 1	V 2	V 3	….	V15
Primary outcome						
First occurring onset or hospitalisation for heart disease, stroke, or heart failure§<br>(time from randomisation)		x	x	x	x	x
Secondary outcomes						
For all patients:						
Systolic blood pressure	x	x (annually)				x
Diastolic blood pressure	x	x (annually)				x
Hypotension		x	x	x	x	x
BNP†	x	x	x	x	x	x
ACR †	x	x	x	x	x	x
eGFR†	x	x	x	x	x	x
EQ-5D-5L†	x	x	x	x	x	x
TIA		x	x	x	x	x
For intensively phenotyped group:						
Ambulatory blood pressure†	x					x
Carotid-femoral pulse wave velocity	x					x

* Baseline visit; § time from randomisation; †change from randomisation

APPENDIX III. FLOW DIAGRAM OF TRIAL PARTICIPANTS

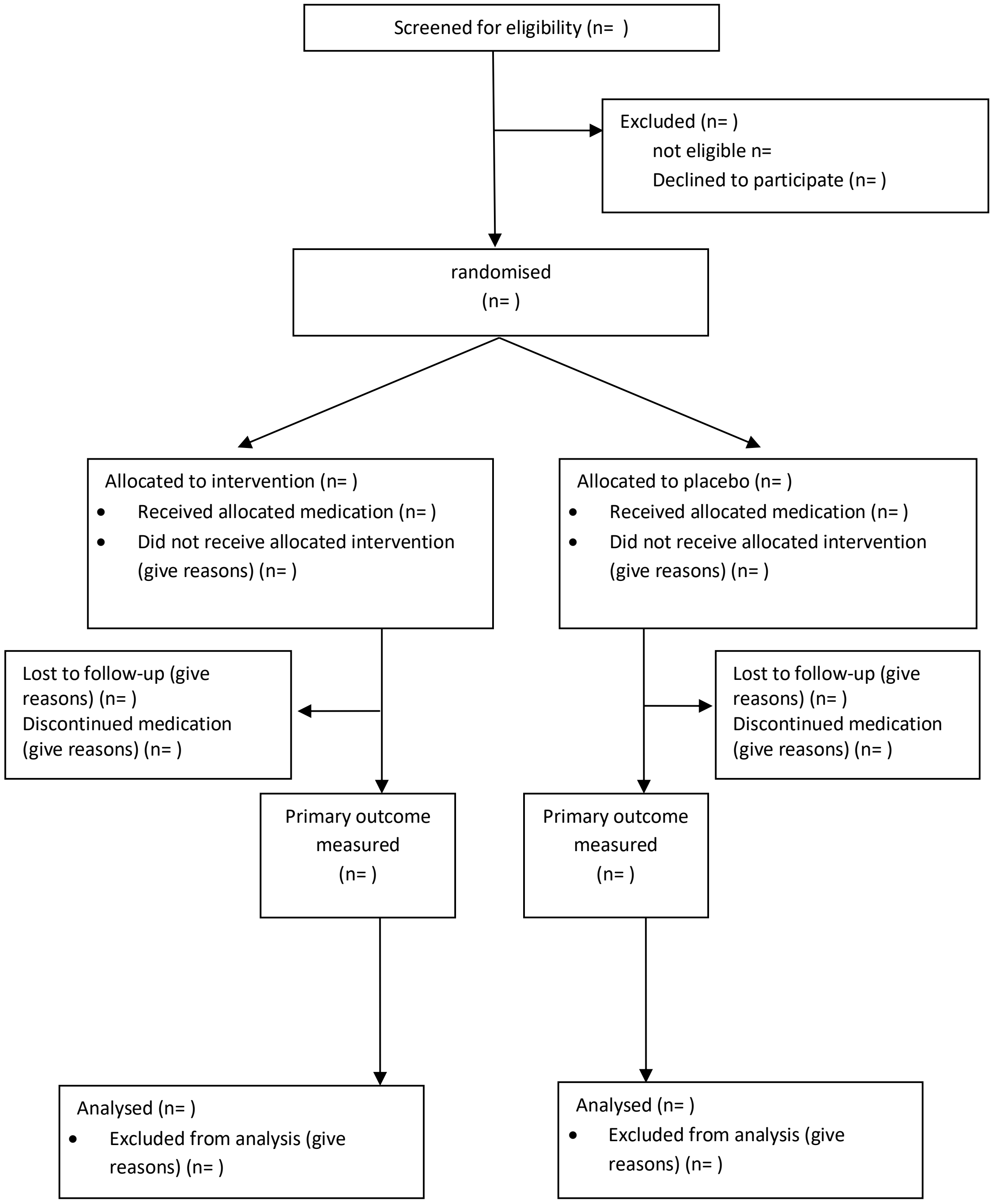

Supplement: Supplementary file 1 — Supplementary Tables 1 and 2, study protocol and statistical analysis plan. [file 41591_2024_3263_MOESM1_ESM.pdf]
