# Supplementary material for: Low-dose spironolactone and cardiovascular outcomes in moderate stage chronic kidney disease: a randomized controlled trial
Source: Nat Med. 2024 Sep 30;30(12):3634–45. doi: 10.1038/s41591-024-03263-5 (PMC11753262; doi:10.1038/s41591-024-03263-5)
Supplement: Supplementary file 2 — Reporting Summary [file 41591_2024_3263_MOESM2_ESM.pdf]

nature portfolio

Corresponding author(s): Professor F.D. Richard Hobbs and Dr Nicholas R. Jones

Last updated by author(s): Aug 5, 2024

Reporting Summary

Nature Portfolio wishes to improve the reproducibility of the work that we publish. This form provides structure for consistency and transparency in reporting. For further information on Nature Portfolio policies, see our Editorial Policies and the Editorial Policy Checklist.

Statistics

For all statistical analyses, confirm that the following items are present in the figure legend, table legend, main text, or Methods section.

n/a	Confirmed	
☐	☒	The exact sample size (*n*) for each experimental group/condition, given as a discrete number and unit of measurement
☐	☒	A statement on whether measurements were taken from distinct samples or whether the same sample was measured repeatedly
☐	☒	The statistical test(s) used AND whether they are one- or two-sided<br>*Only common tests should be described solely by name; describe more complex techniques in the Methods section.*
☐	☒	A description of all covariates tested
☐	☒	A description of any assumptions or corrections, such as tests of normality and adjustment for multiple comparisons
☐	☒	A full description of the statistical parameters including central tendency (e.g. means) or other basic estimates (e.g. regression coefficient) AND variation (e.g. standard deviation) or associated estimates of uncertainty (e.g. confidence intervals)
☐	☒	For null hypothesis testing, the test statistic (e.g. *F*, *t*, *r*) with confidence intervals, effect sizes, degrees of freedom and *P* value noted<br>*Give P values as exact values whenever suitable.*
☒	☐	For Bayesian analysis, information on the choice of priors and Markov chain Monte Carlo settings
☒	☐	For hierarchical and complex designs, identification of the appropriate level for tests and full reporting of outcomes
☒	☐	Estimates of effect sizes (e.g. Cohen's *d*, Pearson's *r*), indicating how they were calculated

Our web collection on statistics for biologists contains articles on many of the points above.

Software and code

Policy information about availability of computer code

Data collection	Clinical trial data is collected by the Clinical Trials Unit both electronically and in paper format. All Study Data Documents (SDDs) in paper format are date stamped upon receipt and tracked within a trial management database. A full pre-entry review ensures that all pages have been received, IDs are consistent and obvious errors/ missing data are appropriately addressed prior to entry. All SDDs are double entered by two independent staff into the clinical database.
Data analysis	Analysis was conducted using existing software, including SPSS 25 and Stata version 16.1 (SE). The scripts for the statistical analysis are freely available from: https://github.com/samjamesmort/BARACK-D_Statistical_Analysis. We did not develop novel algorithms or analysis within this study.

For manuscripts utilizing custom algorithms or software that are central to the research but not yet described in published literature, software must be made available to editors and reviewers. We strongly encourage code deposition in a community repository (e.g. GitHub). See the Nature Portfolio guidelines for submitting code & software for further information.

Data

Policy information about availability of data

All manuscripts must include a data availability statement. This statement should provide the following information, where applicable:

- Accession codes, unique identifiers, or web links for publicly available datasets
- A description of any restrictions on data availability
- For clinical datasets or third party data, please ensure that the statement adheres to our policy

All data are securely stored under the Data Protection Act 2004 and adhere to the Primary Care Clinical Trials Unit data sharing standard operating procedure in which data sharing agreements have to be approved by both the Trial Management Group and the sponsor. All available data can be obtained by contacting the Chief Investigator (Professor Richard Hobbs, richard.hobbs@phc.ox.ac.uk). Individual patient data will be shared in data sets in a deidentified and anonymized format, following our data sharing process. We will aim to make data available within six to nine weeks. We are working towards having a fully de-identified dataset available to publicly share, but this has not been completed at the time of publication.

Research involving human participants, their data, or biological material

Policy information about studies with human participants or human data. See also policy information about sex, gender (identity/presentation), and sexual orientation and race, ethnicity and racism.

Reporting on sex and gender	Fairly equal numbers of men and women were recruited into the study. We refer to gender in the manuscript and report on how this was determined in the Methods. The study findings apply to both genders and we have reported the primary outcome and safety data separately for men and women to show that the results were similar, although this was a post-hoc analysis.
Reporting on race, ethnicity, or other socially relevant groupings	We report on ethnicity, using grouping consistent with the approach taken by the UK government 2021 Census. In total 96.6% of the participants in the study were of white ethnicity and it was not appropriate to conduct sub-group analysis within ethnic groups given the very low numbers of participants in any other ethnic group.
Population characteristics	In total, 1,985 participants attended a screening visit between 6th December 2013 and 31st August 2018 with 1,434 participants randomised into the study. We report on the participant characteristics in the 'participant disposition' section of the manuscript, as well as providing data in Table 1. The text is as follows: "At the time of randomisation, the mean age of participants was 74.8 years (standard deviation (SD) 8.1) with 54.5% women (Table 1). There was a high burden of comorbid cardiovascular disease among the population, including hypertension (76.7%), type 2 diabetes (24.3%), ischaemic heart disease (17.4%) and atrial fibrillation (12.2%). The mean eGFR at baseline was 43.5 ml/min/1.73m2 (SD 6.9) and ACR 1.5mg/mmol (interquartile range (IQR) 0.6 to 4.3). The majority of participants were prescribed either an ACEi (40.0%) or ARB (36.6%). Only 4 participants were prescribed a sodium-glucose co-transporter-2 (SGLT2) inhibitor. The study population was well balanced between groups."
Recruitment	Recruitment into the study was described in the previously published protocol paper (https://trialsjournal.biomedcentral.com/articles/10.1186/1745-6215-15-160) as follows:<br>"The study is set in primary care where patients will primarily be identified opportunistically by their primary care clinician or systematically from existing CKD lists. They will be invited if they have been formally diagnosed with CKD stage 3b or there is evidence of stage 3b CKD from blood results (eGFR 30–44 mL/min/1.73 m2[2]) and fulfil the other inclusion/exclusion criteria. Potentially eligible patients will be invited to attend a baseline clinic at their own practice where the trial will be explained. Informed consent will be obtained and baseline assessments performed."
Ethics oversight	Trial steering and data monitoring committees supervised the trial. The study was approved by a National Health Service (NHS) Research Ethics Committee (REC-13/SC/0114) as well as the Medicines and Healthcare Regulatory Authority (MHRA), relevant NHS Research and Development departments and the host institutions.

Note that full information on the approval of the study protocol must also be provided in the manuscript.

Field-specific reporting

Please select the one below that is the best fit for your research. If you are not sure, read the appropriate sections before making your selection.

☒ Life sciences ☐ Behavioural & social sciences ☐ Ecological, evolutionary & environmental sciences

For a reference copy of the document with all sections, see nature.com/documents/nr-reporting-summary-flat.pdf

Life sciences study design

All studies must disclose on these points even when the disclosure is negative.

Sample size	Based on prior literature, we estimated that the CVD event rate and total mortality rate in people with CKD stage 3b would be 11.29 and 4.76 per 100 person years respectively, giving a combined event rate of 16.05 per 100 person years. To detect a 20% relative risk reduction in death or CVD events within three years in the intervention group as compared with the control group (i.e a hazard ratio of 0.8) with a two-sided significance of 0.05, 1,308 participants per arm would be required, seeking 90% power and assuming 10% drop out rate per year. The

	trial failed to recruit to target achieving 45% of the planned sample size.
Data exclusions	In total, 1,985 participants attended a screening visit between 6th December 2013 and 31st August 2018 with 1,434 participants randomised into the study. Of those randomised, 62 participants were identified as ineligible for the study by the supervising trials unit, including 33 participants who had been randomised to spironolactone and 29 randomised to usual care, leaving 1,372 participants who were included in the statistical analyses.
Replication	A study protocol and statistical analysis plan were published in advance of the study. The analysis was conducted by two statisticians to check reproducibility. Outcomes were adjudicated by an independent committee who were blinded to treatment allocation. All attempts at reproducibility were successful.
Randomization	Eligible participants were randomised in a 1:1 ratio to usual care or usual care plus treatment with spironolactone 25mg once daily by their usual doctor. Randomisation was carried out using Sortition, a validated randomisation system, developed within our Primary Care Clinical Trials Unit. Participants were enrolled from 329 general practice sites across the UK. We stratified by GP practice to ensure a balance of the two arms within each practice.
Blinding	BARACK-D was a prospective open blinded end-point (PROBE) trial, in which neither the participants nor their treating healthcare professional were blinded to their treatment allocation. The investigators and independent endpoint committee were blinded to the particpants' treatment arm until the completion of the trial.

Reporting for specific materials, systems and methods

We require information from authors about some types of materials, experimental systems and methods used in many studies. Here, indicate whether each material, system or method listed is relevant to your study. If you are not sure if a list item applies to your research, read the appropriate section before selecting a response.

Materials & experimental systems

n/a	Involved in the study
☒	☐ Antibodies
☒	☐ Eukaryotic cell lines
☒	☐ Palaeontology and archaeology
☒	☐ Animals and other organisms
☐	☒ Clinical data
☒	☐ Dual use research of concern
☒	☐ Plants

Methods

n/a	Involved in the study
☒	☐ ChIP-seq
☒	☐ Flow cytometry
☒	☐ MRI-based neuroimaging

Clinical data

Policy information about clinical studies

All manuscripts should comply with the ICMJE guidelines for publication of clinical research and a completed CONSORT checklist must be included with all submissions.

Clinical trial registration	The trial was registered prospectively on the ISRCTN registry 2013: ISRCTN44522369. 'Benefits of Aldosterone Receptor Antagonism in Chronic Kidney Disease (BARACK-D) Trial: A potential new treatment for kidney disease', available from https://www.isrctn.com/ISRCTN44522369
Study protocol	The study protocol has been submitted to Nature Medicine along with the main manuscript. A protocol paper was published in 2014: Hill, N.R., Lasserson, D., Thompson, B. et al. Benefits of Aldosterone Receptor Antagonism in Chronic Kidney Disease (BARACK D) trial–a multi-centre, prospective, randomised, open, blinded end-point, 36-month study of 2,616 patients within primary care with stage 3b chronic kidney disease to compare the efficacy of spironolactone 25 mg once daily in addition to routine care on mortality and cardiovascular outcomes versus routine care alone: study protocol for a randomized controlled trial. Trials 15, 160 (2014). https://doi.org/10.1186/1745-6215-15-160
Data collection	Study screening visits took place between 6th December 2013 and 31st August 2018 at participating general practice sites within the UK. All participant site visits were also conducted at their own general practice, with oversight provide by regional coordinating study site centres. Follow-up continued until all active participants reached the end of follow-up as per protocol.<br><br>We described the approach to Data collection in our protocol paper as follows:<br>"Source documents will include:<br>- Primary care electronic and paper records/outputs<br>- Reports from laboratory investigations<br>- Hospital correspondence<br>- Records of 24-h ambulatory and home BP measurements<br>- Patient questionnaires<br>- Patient diary cards<br>- The case report form (CRF) itself where there is no other written or electronic record of data<br><br>Clinical trial data is collected by the PC-CTU both electronically and in paper format, with a paper back-up for the data captured electronically.

All documents will be stored safely in confidential conditions according to PC-CTU policies and SOPs. On all study-specific documents, other than the signed consent, the participant will be referred to by the study participant number/code, not by name. Study documentation will be archived for a period of 5 years according to PC-CTU SOPs.

Source data will be verified as appropriate by the PC-CTU Quality Manager or delegate using a risk-based approach and will be defined in the monitoring plan."

Outcomes

The primary outcome was the time from randomisation until the first occurrence of any of the following events; death, hospitalisation for heart disease (coronary heart disease, arrhythmia, atrial fibrillation, sudden death or failed sudden death, defined as a cardiac arrest where the participant was successfully resuscitated), stroke, heart failure, transient ischaemic attack or PAD, or first onset of any condition listed not present at baseline.

Pre-specified secondary outcomes included changes in blood pressure, natriuretic peptides, ACR and eGFR recorded in the primary care record across follow-up as well as rates of adverse events, including hyperkalaemia and hypotension. Hyperkalaemia was considered a serum potassium of 5.5mmol/L or greater, sub-categorised as 5.5 to 5.9mmol/L, 6.0 to 6.4mmol/L and ≥6.5mmol/L. Hypotension was defined as systolic blood pressure <100mmHg or a drop in the systolic pressure of >20 mmHg on standing. Progression of kidney disease was defined as a ≥30% increase in creatinine from baseline, a drop of ≥25% in eGFR from baseline or a ≥20% drop in eGFR from the previous result.

An independent panel of three senior clinicians adjudicated each of the endpoints, using all the available clinical information independently of each other, with discussion determining the endpoint where there was initial disagreement.

Plants

Seed stocks

Not applicable

Novel plant genotypes

Not applicable

Authentication

Not applicable
